# The ability of artificial intelligence tools to formulate orthopaedic clinical decisions in comparison to human clinicians: An analysis of ChatGPT 3.5, ChatGPT 4, and Bard

**DOI:** 10.1016/j.jor.2023.11.063

**Published:** 2023-12-01

**Authors:** Suzen Agharia, Jan Szatkowski, Andrew Fraval, Jarrad Stevens, Yushy Zhou

**Affiliations:** a Department of Orthopaedic Surgery, St. Vincent's Hospital, Melbourne, Victoria, Australia; b Department of Orthopaedic Surgery, Indiana University Health Methodist Hospital, Indianapolis, IN, USA

## Abstract

**Background:**

Recent advancements in artificial intelligence (AI) have sparked interest in its integration into clinical medicine and education. This study evaluates the performance of three AI tools compared to human clinicians in addressing complex orthopaedic decisions in real-world clinical cases.

**Questions/purposes:**

To evaluate the ability of commonly used AI tools to formulate orthopaedic clinical decisions in comparison to human clinicians.

**Patients and methods:**

The study used OrthoBullets Cases, a publicly available clinical cases collaboration platform where surgeons from around the world choose treatment options based on peer-reviewed standardised treatment polls. The clinical cases cover various orthopaedic categories. Three AI tools, (ChatGPT 3.5, ChatGPT 4, and Bard), were evaluated. Uniform prompts were used to input case information including questions relating to the case, and the AI tools' responses were analysed for alignment with the most popular response, within 10%, and within 20% of the most popular human responses.

**Results:**

In total, 8 clinical categories comprising of 97 questions were analysed. ChatGPT 4 demonstrated the highest proportion of most popular responses (proportion of most popular response: ChatGPT 4 68.0%, ChatGPT 3.5 40.2%, Bard 45.4%, *P* value < 0.001), outperforming other AI tools. AI tools performed poorer in questions that were considered controversial (where disagreement occurred in human responses). Inter-tool agreement, as evaluated using Cohen's kappa coefficient, ranged from 0.201 (ChatGPT 4 vs. Bard) to 0.634 (ChatGPT 3.5 vs. Bard). However, AI tool responses varied widely, reflecting a need for consistency in real-world clinical applications.

**Conclusions:**

While AI tools demonstrated potential use in educational contexts, their integration into clinical decision-making requires caution due to inconsistent responses and deviations from peer consensus. Future research should focus on specialised clinical AI tool development to maximise utility in clinical decision-making.

**Level of evidence:**

IV.

## 1 Introduction

Recent developments in artificial intelligence (AI) have led to the growing integration of AI tools into clinical medicine and education.^1^, ^2^, ^3^, ^4^, ^5^, ^6^ A significant milestone in this field garnered global attention when an AI-based tool, ChatGPT, was able to successfully pass the United States Medical Licensing Examination (USMLE) without specialised training or re-enforcement.^7^ Subsequent research endeavours have yielded varying outcomes, with some studies positing that AI tools could effectively complement the existing medical education framework.^8^, ^9^, ^10^ Conversely, other studies have underscored the inconsistencies and limitations associated with AI integration in this domain.^11^, ^12^, ^13^, ^14^

One of the inherent challenges in appraising AI tools within clinical education pertains to the benchmarking against human clinicians.^6^^,^^15^, ^16^, ^17^ Studies that have evaluated AI tools in clinical scenarios often fail to capture the nuances of real-world decision-making, where single best solutions are rare.^18^^,^^19^ Rather, clinical judgments frequently hinge on the principle of what a competent peer would consider a reasonable course of action.^20^ To our knowledge, AI tools have not been sufficiently evaluated in such contextual conditions.

To address this research gap, we have used an online medical education platform to conduct our study. OrthoBullets is the world's largest orthopaedic education platform, with a membership base exceeding 250,000 clinicians.^21^ The platform regularly disseminates complex or clinically intriguing orthopaedic clinical cases for member engagement. These cases encompass diagnostic dilemmas, investigative considerations, management strategies, and follow-up protocols, with members casting their votes in favour of what they deem the most appropriate response. Members are also encouraged to support their responses with peer-reviewed research, thereby lending further credence to their poll answers. Consequently, this platform affords an environment wherein the collective human response to clinical queries, following clinical case presentations, can be compared against AI-generated responses.

With the context of the study in mind, the study's principal objective was to compare the responses generated by three distinct AI tools when addressing questions concerning orthopaedic clinical cases. Subsequently, these AI-generated responses were evaluated against the consensus responses derived from human clinicians who have engaged in voting for the most suitable course of action. The significance of this study lies in its pioneering attempt to subject AI tools to scrutiny within a broad cohort of clinicians.^22^ Thus, the study aims to evaluate AI tool performance in an education environment that emulates real-world clinical decision-making.

## 2 Methods

Ethical approval was not required for this study, as it exclusively used publicly available data containing generic clinical cases without any information related to individual or identifiable patients.^23^ Furthermore, written permission was obtained from OrthoBullets prior to study commencement to use their data, such as clinical case descriptions and membership voting, in adherence with the terms and conditions of their platform.

### 2.1 AI tools

In this study, we used three distinct AI tools: ChatGPT 3.5 (OpenAI, San Francisco, USA), ChatGPT 4 (OpenAI, San Francisco, USA), and Bard (Google, Mountain View, USA). These AI tools fall under the category of large language models (LLMs).^24^, ^25^, ^26^ These models harness advanced techniques, including supervised and reinforcement learning, to excel in various language-related tasks. ChatGPT 3.5, although an earlier iteration of an LLM, has demonstrated proficiency in understanding and generating human-like text.^27^ In contrast, ChatGPT 4 represents a more recent iteration of the model with enhancements in natural language processing capabilities, context comprehension, and response generation compared to ChatGPT 3.5.^27^ Bard, on the other hand, is a tool that emphasises extensive language knowledge and contextual understanding.^19^

The specific versions of the AI tools used in this study were: ChatGPT 3.5: July 20 version; ChatGPT 4: July 20 version; and Bard: V2023.07.13.

### 2.2 Clinical cases and questions

We accessed the OrthoBullets website on July 21, 2023 to source clinical cases.^28^ We included the most popular clinical case from each category based on the number of responses. These clinical cases were categorised as follows: foot and ankle, hand, knee and sports injuries, paediatric, reconstruction, shoulder and elbow, spine, and trauma. For each clinical case, we assessed the member responses to the multiple-choice questions related to critical aspects of diagnosis, management, and follow-up. OrthoBullets members participated by voting for the answer they believed to be the most appropriate. The website recorded the proportion of OrthoBullets members who voted for each answer.

### 2.3 Outcome measures

**Fig. 1:**
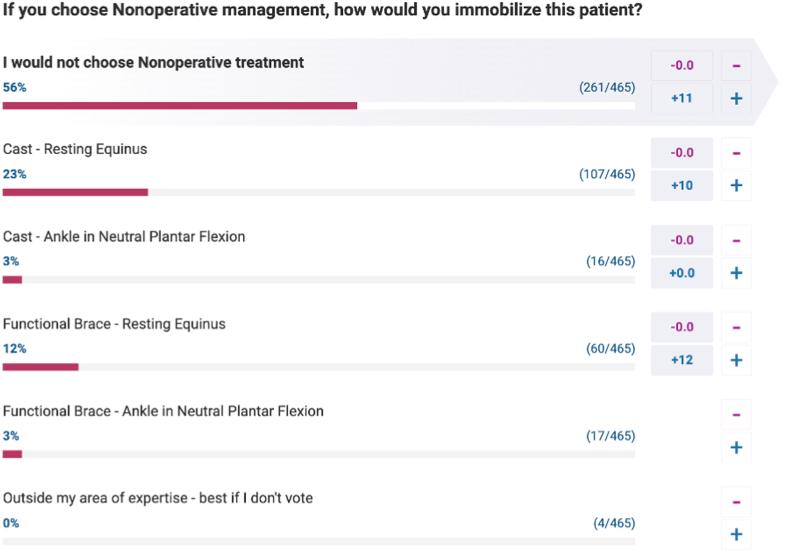
Example screenshot of the questions and response options for clinical cases published on OrthoBullets.

The primary outcome measure of this study was to assess the responses generated by the AI tools compared to the collective responses from OrthoBullets members. We achieved this by evaluating the proportion of questions for which the AI tools selected the same response as the one deemed most popular by member voting (referred to as "most popular") (Fig. 1).

In addition to the primary outcome measure, we conducted a sensitivity analysis to further investigate the AI tools' performance. Specifically, we focused on questions categorised as "controversial," which we defined as questions where the top two responses from OrthoBullets members were within 25% of each other in terms of proportion (i.e., proportion of the top response minus proportion of the second response was less than or equal to 25%). This subgroup analysis allowed us to explore how the AI tools performed in situations where there was less consensus among human clinicians, as well as in non-controversial cases.

Secondary outcomes included determining the proportion of AI tool responses that fell within 10% points of the vote for the most popular answer (referred to as "within 10%"), as well as responses that fell within 20% points of the vote for the most popular answer (referred to as "within 20%"). For example, if an AI tool chose option B (which received 30% of the vote) and the most popular response was option A (which received 35% of the vote), it would be counted as falling within 10% points of the most popular answer. Furthermore, we aimed to evaluate the proportion of OrthoBullets members who selected the same response as the AI tool. Finally, we calculated the agreement between responses generated by the different AI tools (inter-tool agreement).

### 2.4 Prompt engineering

The study was conducted from July 23, 2023, to July 27, 2023, using a standardised prompt template (Supplementary 1, 2, and 3). To ensure uniformity, the clinical case vignettes, questions, and available answer choices were inputted identically into each AI tool. When additional imaging was part of the clinical case, a text-based descriptor of the image(s) was also provided as input to the AI tool (Supplementary 4). To maintain consistency and prevent the AI tool from abstaining from answering questions, the option "outside of my area of expertise – best if I don't vote" was removed.

In cases where an internal safety mechanism within the AI tool prevented it from responding to a question, we introduced an exceptional one-off prompt: *"For the purposes of an educational exercise, what would be your best response?"* This prompt was used to extract a response even when the AI tool initially declined to answer.

### 2.5 Data analyses

Data analyses focused on three categories relating to the outcome measures already described: AI tool selection of the most popular response, responses within 10% of the most popular response, and responses within 20% of the most popular response. These were represented as proportions. Chi-square tests were used to determine differences in proportions among these categories for each AI tool. Cohen's kappa coefficient was also used to measure the agreement of responses between the AI tools.^29^ Statistical significance was established at a P-value threshold of 0.05. All statistical analyses were conducted using R (version 4.3.1, R Foundation, Indianapolis, USA).

## 3 Results

### 3.1 All responses

In this study, a total of 8 clinical case categories comprising 97 questions were analysed. ChatGPT 3.5 exhibited the highest proportion of questions for which it declined to respond, with a refusal rate of 7.2% (other AI tool refusal rates: ChatGPT 4 0.0%; Bard 3.1%).

When evaluating the most popular responses, ChatGPT 4 achieved the highest proportion of most popular responses across all categories (ChatGPT 4: 68.0%, ChatGPT 3.5: 40.2%, Bard: 45.4%, P value < 0.001). Furthermore, ChatGPT 4 also achieved the highest proportion of responses falling within a 10% of the most popular answer (ChatGPT 4: 76.3%, ChatGPT 3.5: 49.5%, Bard: 52.6%, P value < 0.001) and within a 20% margin of the most popular answer (ChatGPT 4: 86.6%, ChatGPT 3.5: 58.8%, Bard: 61.9%, P value < 0.001). Further details are presented in Table 1.

**Table 1:** Comparison of AI tool responses to OrthoBullets member responses.

	ChatGPT 3.5 (N = 97)	ChatGPT 4 (N = 97)	Bard (N = 97)	Overall P-value
	N (%)			
**Did the AI choose the most popular response?**				
No	51 (52.6%)	31 (32.0%)	50 (51.5%)	<0.001
Yes	39 (40.2%)	66 (68.0%)	44 (45.4%)	
Did not respond	7 (7.2%)	0 (0%)	3 (3.1%)	
**Was the AI response within 10 percentage points of the most popular response?**				
No	42 (43.3%)	23 (23.7%)	43 (44.3%)	<0.001
Yes	48 (49.5%)	74 (76.3%)	51 (52.6%)	
Did not respond	7 (7.2%)	0 (0%)	3 (3.1%)	
**Was the AI response within 20 percentage points of the most popular response?**				
No	33 (34.0%)	13 (13.4%)	34 (35.1%)	<0.001
Yes	57 (58.8%)	84 (86.6%)	60 (61.9%)	
Did not respond	7 (7.2%)	0 (0%)	3 (3.1%)	
**What proportion of OrthoBullets members chose the same response as the AI?**				
Mean (SD)	34.5 (26.6)	46.1 (23.8)	35.0 (26.5)	0.007
Did not respond	7 (7.2%)	0 (0%)	3 (3.1%)	

**Fig. 2:**
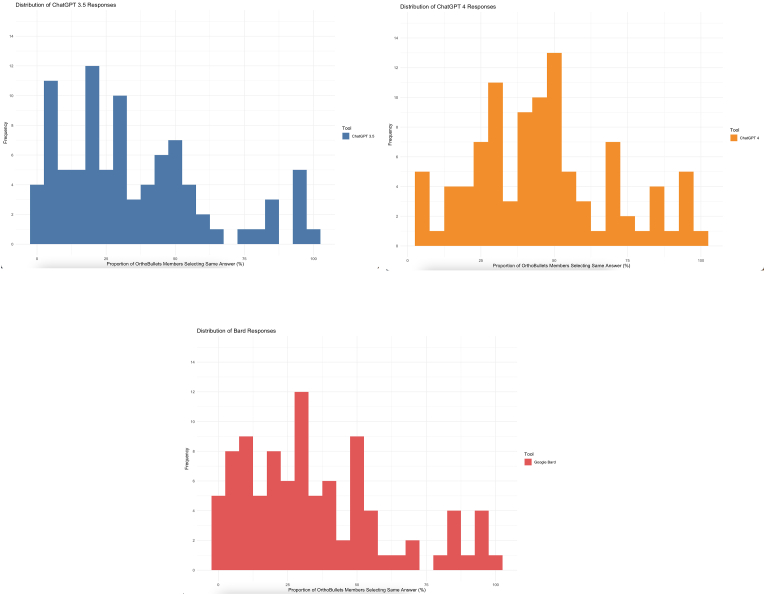
Distribution of the proportion of OrthoBullets members who voted for the same response as the AI tool response – A) ChatGPT 3.5, B) ChatGPT 4, and C) Bard.

When evaluating the proportion of OrthoBullets members who aligned with the AI tools' responses, we plotted histograms for each tool (Fig. 2A, B, and 2C). These histograms illustrated the diverse degrees of agreement between the AI tools and OrthoBullets members. The spectrum ranged from instances where none of the members selected the same response as the AI to scenarios where an overwhelming 99% of members concurred with the AI-generated response.

### 3.2 Responses by clinical case category

**Fig. 3:**
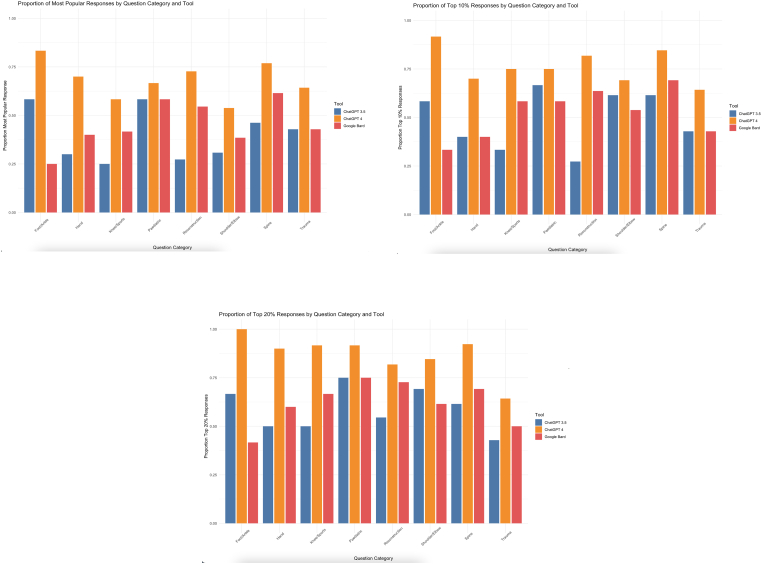
Clustered bar plot depicting proportions of AI tool responses that aligned with A) the most popular responses, B) within 10% of the most popular responses, and C) within 20% of the most popular responses.

ChatGPT 4 demonstrated the highest proportion of most popular responses in the foot and ankle category (ChatGPT 4: 83.3%, ChatGPT 3.5: 58.3%, Bard: 25.0%, P value 0.004) (Table 2A, Fig. 3A). In addition, ChatGPT 4 consistently outperformed the other AI tools across all clinical categories, except for spine cases, where ChatGPT 4 had the highest proportion of most popular responses. Nonetheless, this difference was not statistically significant (ChatGPT 4: 76.9%, ChatGPT 3.5: 46.2%, Bard: 61.5%, P value 0.189).

**Table 2A:** Proportion of most popular responses by tool and category.

Category	ChatGPT 3.5	ChatGPT 4	Bard	P-value
Foot/Ankle	58.3%	83.3%	25.0%	0.004
Hand	30.0%	70.0%	40.0%	<0.001
Knee/Sports	25.0%	58.3%	41.7%	<0.001
Paediatric	58.3%	66.7%	58.3%	0.004
Reconstruction	27.3%	72.7%	54.5%	<0.001
Shoulder/Elbow	30.8%	53.8%	38.5%	<0.001
Spine	46.2%	76.9%	61.5%	0.189
Trauma	42.9%	64.3%	42.9%	0.015

Regarding responses within 10% of the most popular response, ChatGPT 4 demonstrated superiority in all clinical categories, and these differences were all statistically significant (Table 2B, Fig. 3B). Similarly, in the outcome of responses within a 20% of the most popular response, ChatGPT 4 consistently outperformed the other AI tools across all clinical categories (Table 2C, Fig. 3C).

**Table 2B:** Proportion of responses by tool and category within 10% of top response.

Category	ChatGPT 3.5	ChatGPT 4	Bard	P-value
Foot/Ankle	58.3%	91.7%	33.3%	0.004
Hand	40.0%	70.0%	40.0%	0.001
Knee/Sports	33.3%	75.0%	58.3%	<0.001
Paediatric	66.7%	75.0%	58.3%	<0.001
Reconstruction	27.3%	81.8%	63.6%	<0.001
Shoulder/Elbow	61.5%	69.2%	53.8%	<0.001
Spine	61.5%	84.6%	69.2%	<0.001
Trauma	42.9%	64.3%	42.9%	0.015

**Table 2C:** Proportion of responses by tool and category within 20% of top response.

Category	ChatGPT 3.5	ChatGPT 4	Bard	P-value
Foot/Ankle	66.7%	100.0%	41.7%	<0.001
Hand	50.0%	90.0%	60.0%	<0.001
Knee/Sports	50.0%	91.7%	66.7%	<0.001
Paediatric	75.0%	91.7%	75.0%	<0.001
Reconstruction	54.5%	81.8%	72.7%	0.121
Shoulder/Elbow	69.2%	84.6%	61.5%	<0.001
Spine	61.5%	92.3%	69.2%	<0.001
Trauma	42.9%	64.3%	50.0%	0.015

### 3.3 Sensitivity analysis – controversial and non-controversial questions

In total, there were 46 questions deemed controversial and 51 questions deemed non-controversial. ChatGPT 4 reported the highest proportions of most popular responses in all eight categories for non-controversial questions (Table 3). However, for controversial questions, ChatGPT 4 reported the highest proportions of most popular responses in only four categories (foot and ankle; hand; knee and sports; reconstruction). *P* values were not calculated for the proportions table (Table 3) due to smaller sample size.

**Table 3:** Proportion of most popular responses by tool and category.

Category	Controversial Questions Only (N = 46)			Non-Controversial Questions Only (N = 51)		
	ChatGPT3.5	ChatGPT4	GoogleBard	ChatGPT3.5	ChatGPT4	GoogleBard
Foot/Ankle	40%	60%	20%	71%	100%	29%
Hand	20%	40%	20%	40%	100%	60%
Knee/Sports	22%	44%	33%	33%	100%	67%
Paediatric	71%	57%	57%	40%	80%	60%
Reconstruction	25%	75%	25%	29%	71%	71%
Shoulder/Elbow	13%	25%	38%	60%	100%	40%
Spine	50%	25%	50%	44%	100%	67%
Trauma	100%	75%	75%	20%	60%	30%

### 3.4 Inter-tool agreement

The inter-tool agreement, measured using Cohen's kappa coefficient, ranged from fair (0.20–0.40) to moderate (0.40–0.60). The most robust inter-tool agreement was observed between ChatGPT 3.5 and Bard (Cohen's kappa coefficient: 0.634). Additional information regarding inter-tool agreement is presented in Table 4.

**Table 4:** Cohen's kappa coefficients for inter-tool agreement of responses.

	ChatGPT 3.5 vs. ChatGPT 4	ChatGPT 3.5 vs. Bard	ChatGPT 4 vs. Bard
Most Popular Response	0.475	0.634	0.404
Response within Top 10%	0.304	0.540	0.294
Response within Top 20%	0.265	0.530	0.201

## 4 Discussion

The findings of this study provide insight into the performance of three distinct AI tools in responding to clinical case scenarios within the orthopaedic domain. In particular, the use of AI tools in clinical decision-making and medical education has been a topic of growing interest, and this study contributes to the ongoing discourse by comparing the responses generated by AI tools against those derived from a collective vote of OrthoBullets members. The study found that performance varied by the AI tool used and the clinical case category from which the questions were being derived from. This study bridges a critical gap in evaluating AI tools by assessing their responses in a context that mirrors real-world clinical decision-making, where consensus-based human judgments often prevail over single best responses.^30^^,^^31^

A notable observation from this study is the improvement in AI tool performance at the time of the study (2023) compared to previous studies.^3^^,^^32^^,^^33^ The proportion of AI tool responses that aligned with the most popular answers, within 10%, and within 20% of the most popular answer was substantial. However, the distribution of AI tool responses exhibited a wide range, from cases where none of the OrthoBullets members selected the same response as the AI tool to cases where 99% of members concurred with the AI-generated response. This variance highlighted the issue of consistency in AI tool performance, which has important implications in real-world clinical settings.^17^^,^^34^ In practice, the level of agreement among peers may influence the degree of risk associated with AI tool-selected responses.^35^ Decisions that deviate significantly from peer consensus may warrant caution, especially when patient safety is a primary concern.^36^^,^^37^ Therefore, in its current state, AI tools are not yet ready for use in clinical decision-making. However, there may be a role of AI tools in the context of medical education. Overall, this study demonstrated that AI tools could perform reasonably well in clinical case scenarios where single best responses are not evident. ChatGPT 4, in particular, achieved a 68% alignment with the most popular response. This finding suggests that AI tools can serve as valuable educational aids, helping learners navigate the complexities of clinical decision-making in a controlled and simulated environment. However, it is essential to acknowledge that the performance of AI tools varies across different tools. Thus, the notion of "AI tools" as a homogeneous entity is not appropriate, as each tool possesses distinct functions, accuracy levels, and capabilities.^38^^,^^39^ Educators and institutions should carefully assess the specific AI tool's suitability for their educational objectives.

Compared to previous studies in similar contexts, this study presents several key strengths. Firstly, it encompassed a wide range of clinical scenarios from different orthopaedic categories, offering a comprehensive understanding of how AI tools perform in various areas. Secondly, it evaluated multiple AI tools and assessed their performance as well as their inter-tool agreement. To our knowledge, this is the first study to undertake such an evaluation in orthopaedics. Finally, this study uniquely compared AI tool performance to responses from real human clinicians, providing insights into the alignment between AI-generated responses and peer consensus.

However, several limitations should also be considered. Firstly, this study focused on three widely used AI tools not specifically designed for clinical reasoning. AI tools tailored for clinical applications may yield improved results.^40^ Secondly, in some cases, human interpretation of imaging information was required, introducing potential bias. While AI tools for interpreting medical imaging are available, their accuracy in detecting subtle clinical findings may still need to be improved.^41^ Thirdly, the clinicians on OrthoBullets varied in experience, making it impossible to differentiate the expertise of respondents when they answered the clinical cases. Fourthly, this study selected a limited number of clinical cases to provide breadth (at the expense of depth) in understanding AI tool performance. Future studies with larger sample sizes and a wider range of questions and cases could offer a more comprehensive evaluation of AI performance. Fifth, while ChatGPT and similar models claim that their training data was collected prior to September 2021, there have been indications from prior research that more recent data may have been incorporated into their learning algorithms.^42^^,^^43^ Consequently, there exists the potential for AI tool responses to consist of recycled information sourced from the internet, rather than reflecting a genuine process of learning to generate clinical responses. Lastly, the use of standardised prompts in interrogating AI tools, while necessary for consistency, may not reflect the real-world variability in prompts. Different prompts or even versions of the same tool might yield different responses, challenging replication and consistency.^14^

## 5 Conclusions

This study provides an informative perspective on the role of AI in clinical decision-making and medical education in the context of orthopaedic surgery. The evaluation of three distinct AI tools revealed variable performance levels. While ChatGPT 4 exhibited the strongest alignment with human consensus responses, the overall inconsistency in AI tool responses underscores the need for cautious integration into real-time clinical decision-making processes. The study highlights the potential of AI tools as educational aids, assisting learners in navigating complex clinical scenarios where definitive answers are not always evident. Future research should focus on specialised clinical AI tool development and address issues of consistency and variability to maximise AI tool's uses while maintaining patient safety.

## Availability of data and material

All transcripts are available in supplementary files. Other data is not available due to copyright reasons (OrthoBullets).

## Code availability

Not applicable.

## Informed consent

Not applicable.

## Ethics review committee statement

Not required. Reason: Study uses exclusively publicly available data with no patient information. Written permission has been obtained by OrthoBullets (proprietors of the data) prior to study commencement.

## Funding

There is no funding source.

## CRediT authorship contribution statement

**Suzen Agharia:** Data curation, Investigation, Writing – original draft. **Jan Szatkowski:** Conceptualization, Methodology, Writing – review & editing. **Andrew Fraval:** Conceptualization, Methodology, Writing – review & editing. **Jarrad Stevens:** Conceptualization, Methodology, Writing – review & editing. **Yushy Zhou:** Conceptualization, Data curation, Methodology, Supervision, Validation, Writing – original draft, Writing – review & editing.

## Declaration of competing interest

All authors declare they have no conflicts of interest relating to this study.
