# Supplementary material for: The ability of artificial intelligence tools to formulate orthopaedic clinical decisions in comparison to human clinicians: An analysis of ChatGPT 3.5, ChatGPT 4, and Bard
Source: J Orthop. 2023 Dec 1;50:1–7. doi: 10.1016/j.jor.2023.11.063 (PMC10749221; doi:10.1016/j.jor.2023.11.063)
Supplement: Multimedia component 1 [file mmc1.zip › Supplementary Files/Supplementary 1 - ChatGPT 3.5 Transcripts.docx]

Supplementary 2 – ChatGPT 3.5 Transcripts

# Title: Achilles tendon rupture in 63F

Category: Foot and Ankle

Popularity: 1

Date: 20230725

Link: <https://www.orthobullets.com/Site/Cases/View/f4b5c84b-ccf8-4497-b6b7-4ea2c8dbb62d>

Images:
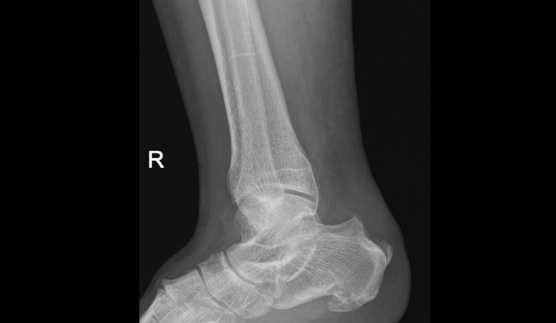

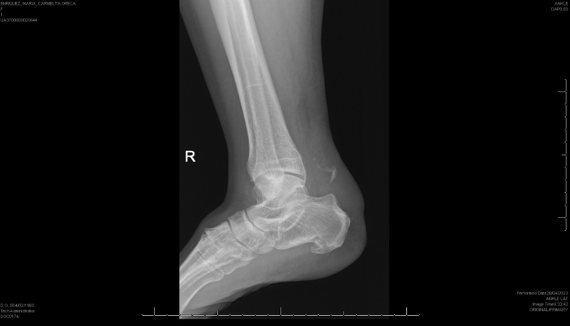

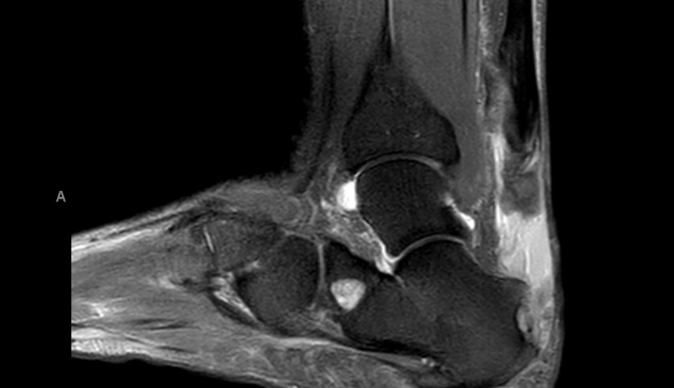


ChatGPT-3.5 dialogue:


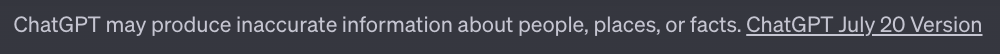


I am going to provide you a clinical vignette. There will be a series of 12 questions to follow. Please choose the single best response for each question.

History of presenting incident:

The patient presents following a fall on the stairs with severe posterior ankle pain, ankle weakness, and inability to walk normally. The patient reports one month prior to the fall she developed mild posterior ankle pain for which she was evaluated, xrays taken (shown), and treated with nonoperative modalities.

Past medical history:

No other morbidities, non-smoker.

Physical exam:

The skin is intact. There is tenderness to palpation over the Achilles and proximal calcaneus. There is a palpable defect/gap at the insertion of the Achilles tendon to the calcaneus. There is weakness to ankle plantar flexion. There is increased passive ankle dorsiflexion. When squeezing the calf, there is no ankle plantar flexion (positive Thompson test).

Imaging findings:

Plain lateral x-ray radiograph of the right foot taken 1 month before the injury shows a calcaneal spur but no acute injury. Plain lateral x-ray radiograph of the right foot taken 1 week before the procedure shows the calcaneal spur has now avulsed, no other acute injury. MRI (single slice, sagittal) of the right foot taken 1 week before the procedure shows the achilles tendon rupture at the insertion of the calcaneus.

Q1: In addition to standard AP and LAT (2-views) radiographs, what additional imaging would you order to guide management?

- None, AP and LAT radiographs are sufficient
- Additional Radiographic Views (aXR) Alone
- Ultrasound (US) Alone
- CT scan (CT) Alone
- MRI Alone
- aXR + US
- aXR + CT
- aXR + MRI
- US + CT
- US + MRI
- CT + MRI
- Other Combination of Above


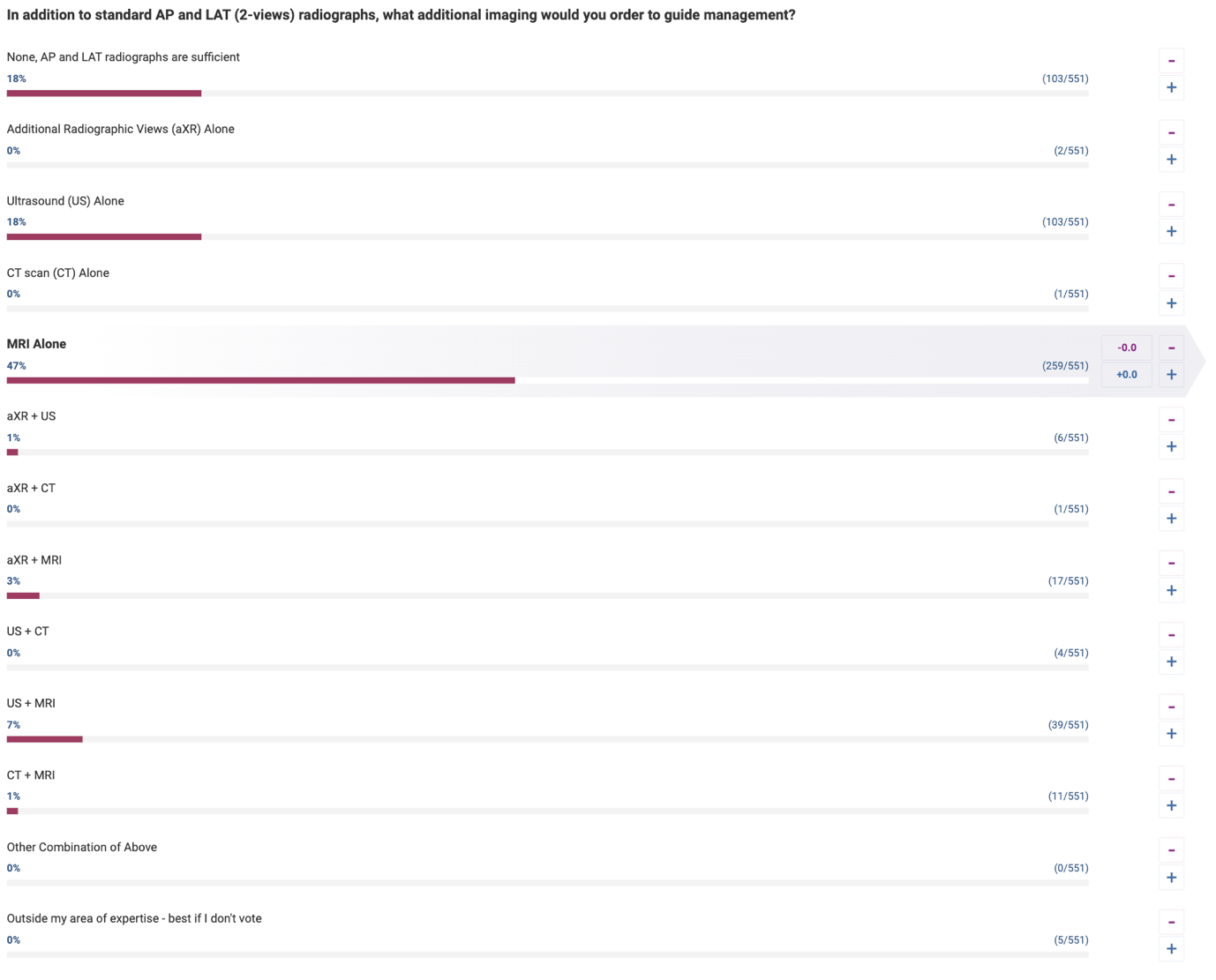


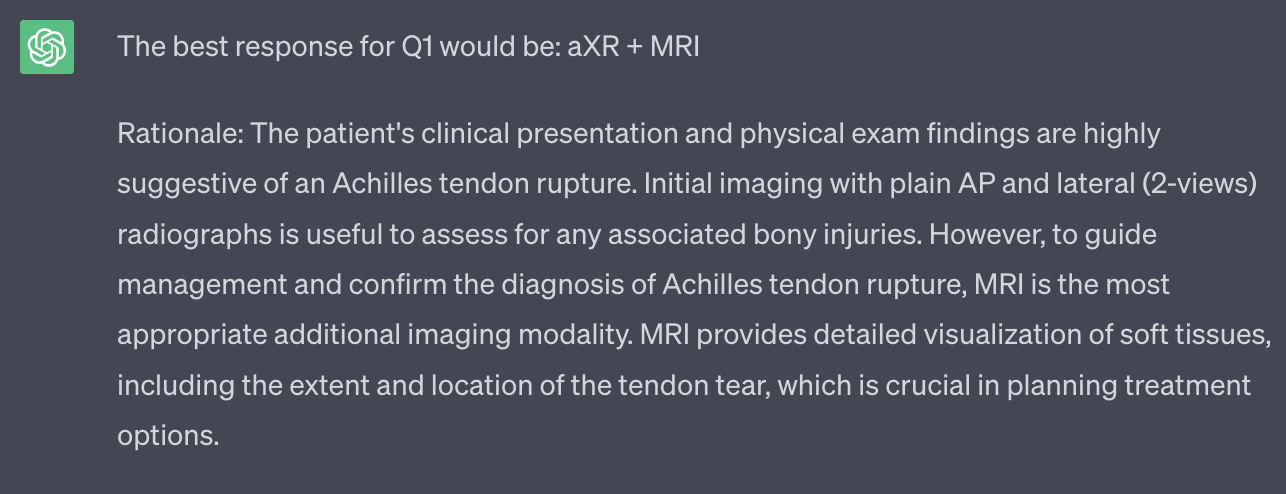


Q2: How would you anatomically classify this Achilles Tendon Tear?

- Muscle-Tendon Junction Rupture
- Midsubstance Tendon Tear
- Tendon Insertion Rupture
- Bony Avulsion Rupture
- Other


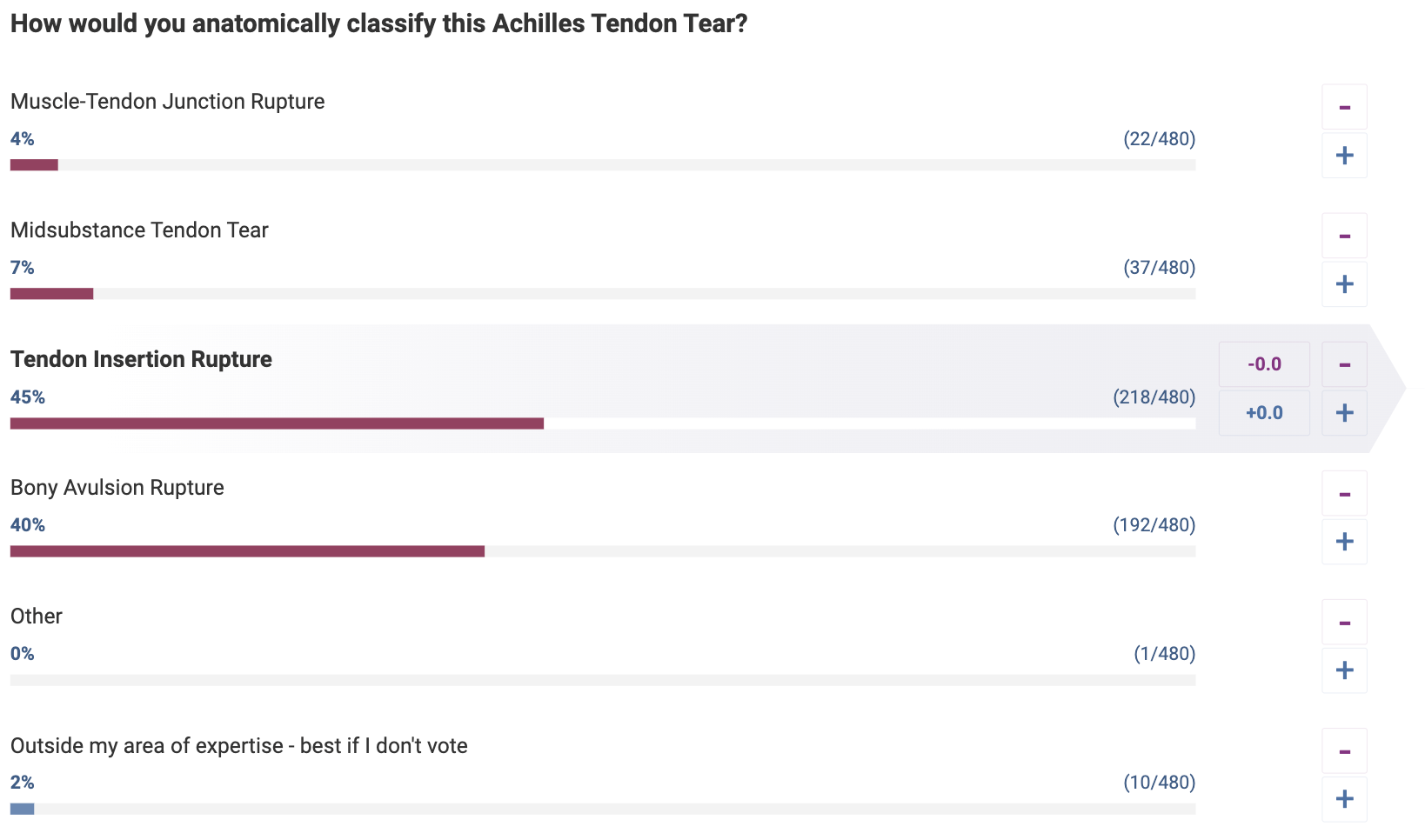


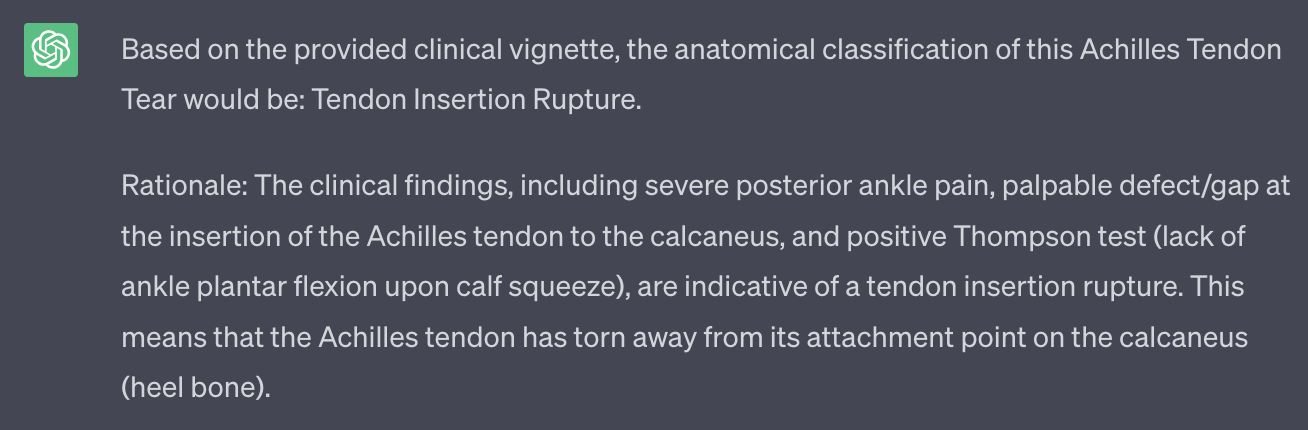


Q3: How would you manage this patient?

- Nonoperative
- Operative
- I need more clinical information to decide


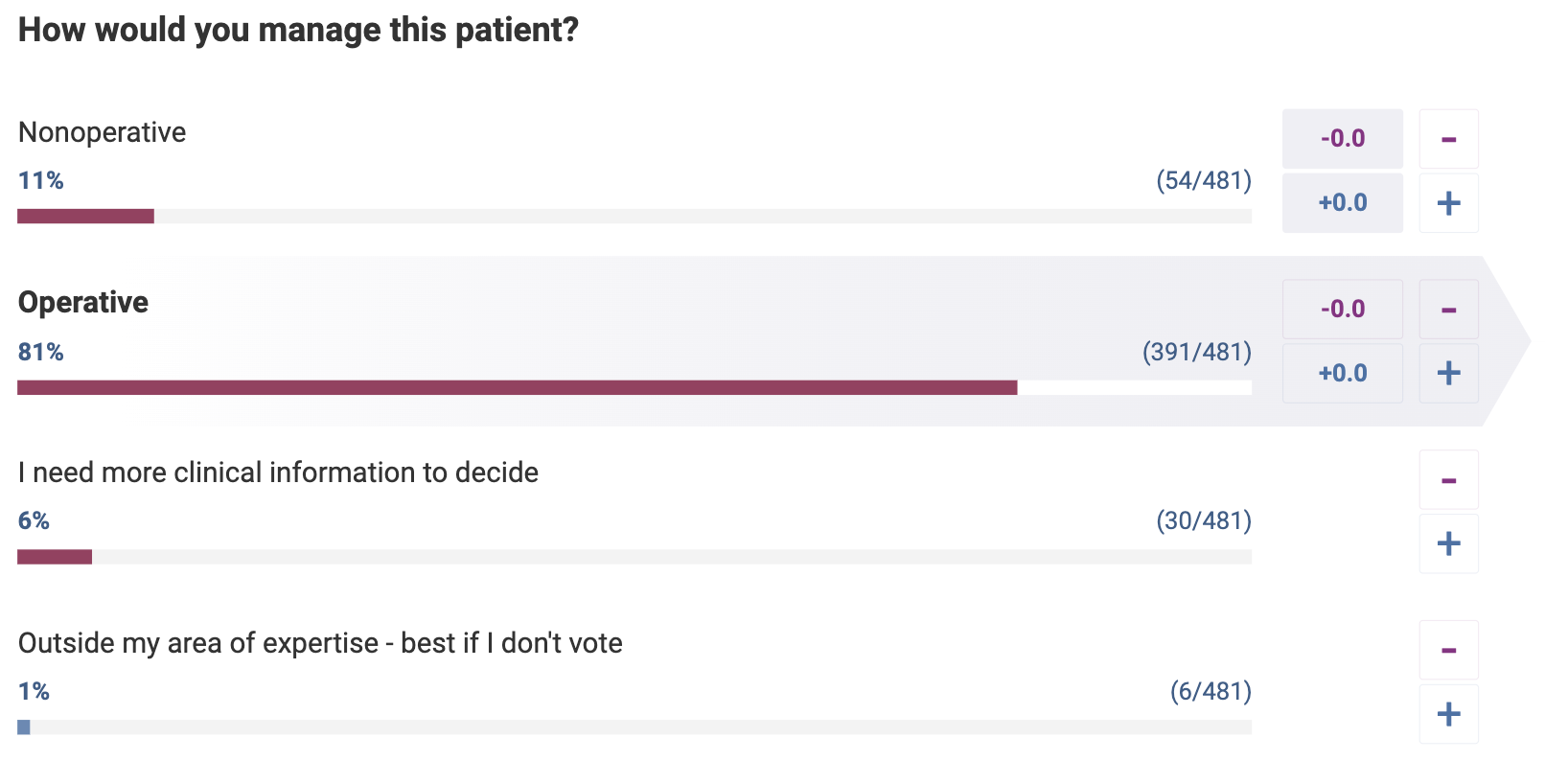


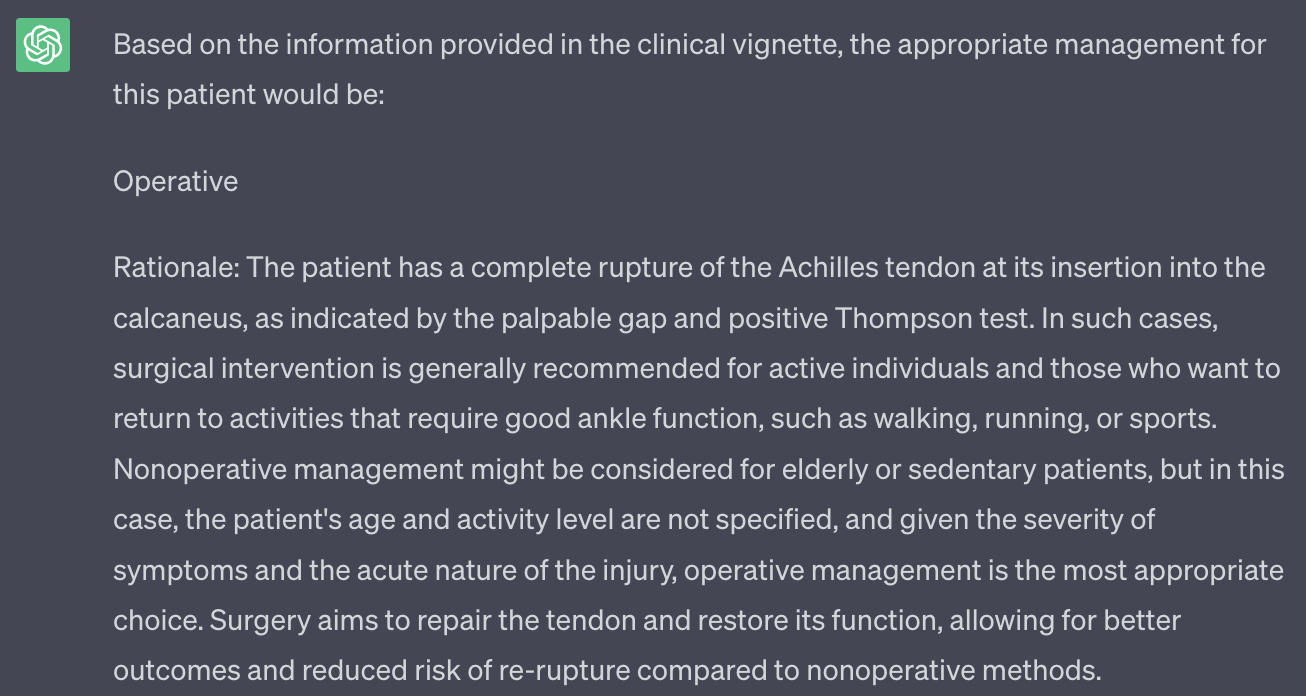


Q4: If you choose Nonoperative management, how would you immobilize this patient?

- I would not choose Nonoperative treatment
- Cast - Resting Equinus
- Cast - Ankle in Neutral Plantar Flexion
- Functional Brace - Resting Equinus
- Functional Brace - Ankle in Neutral Plantar Flexion


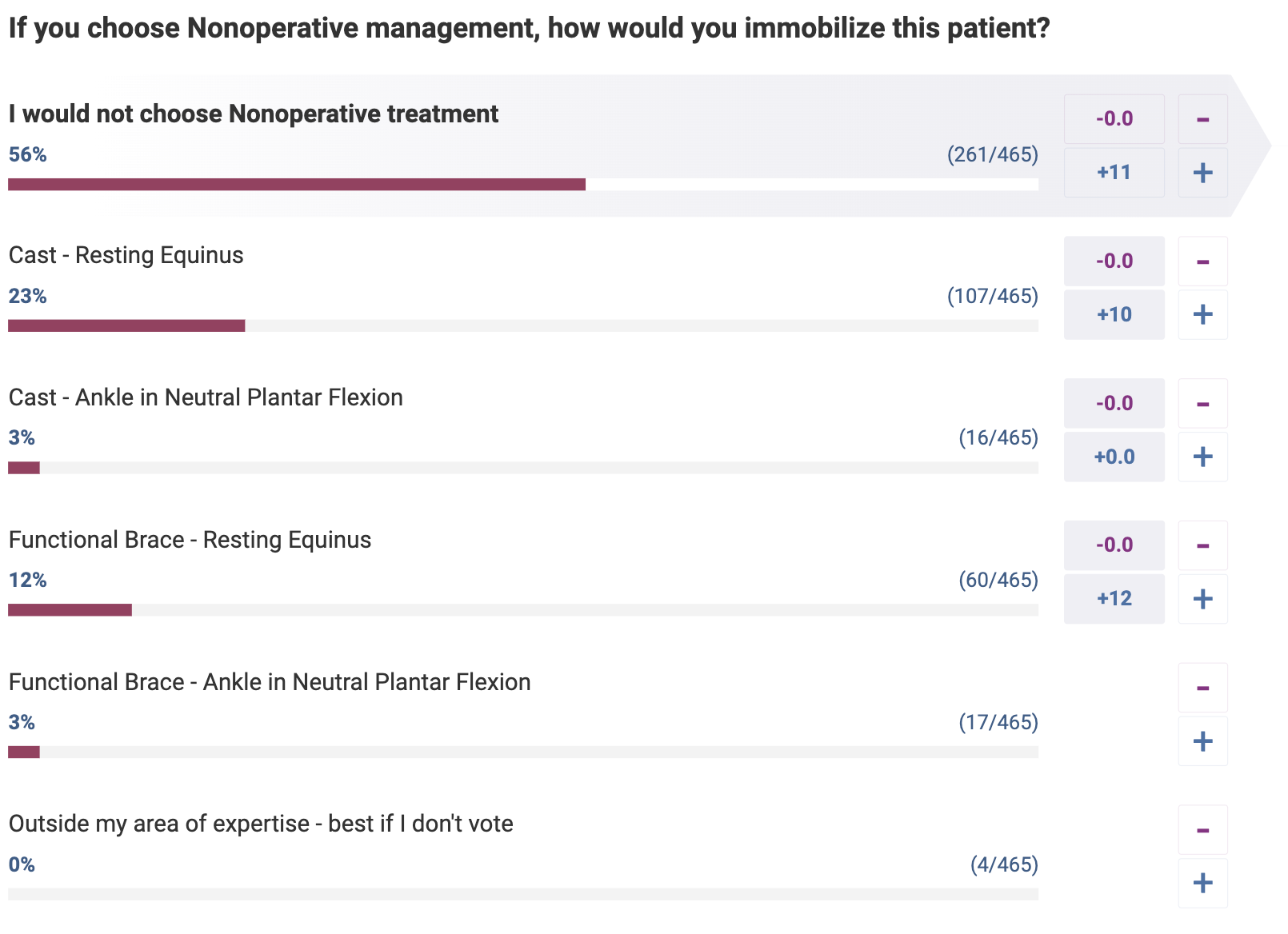


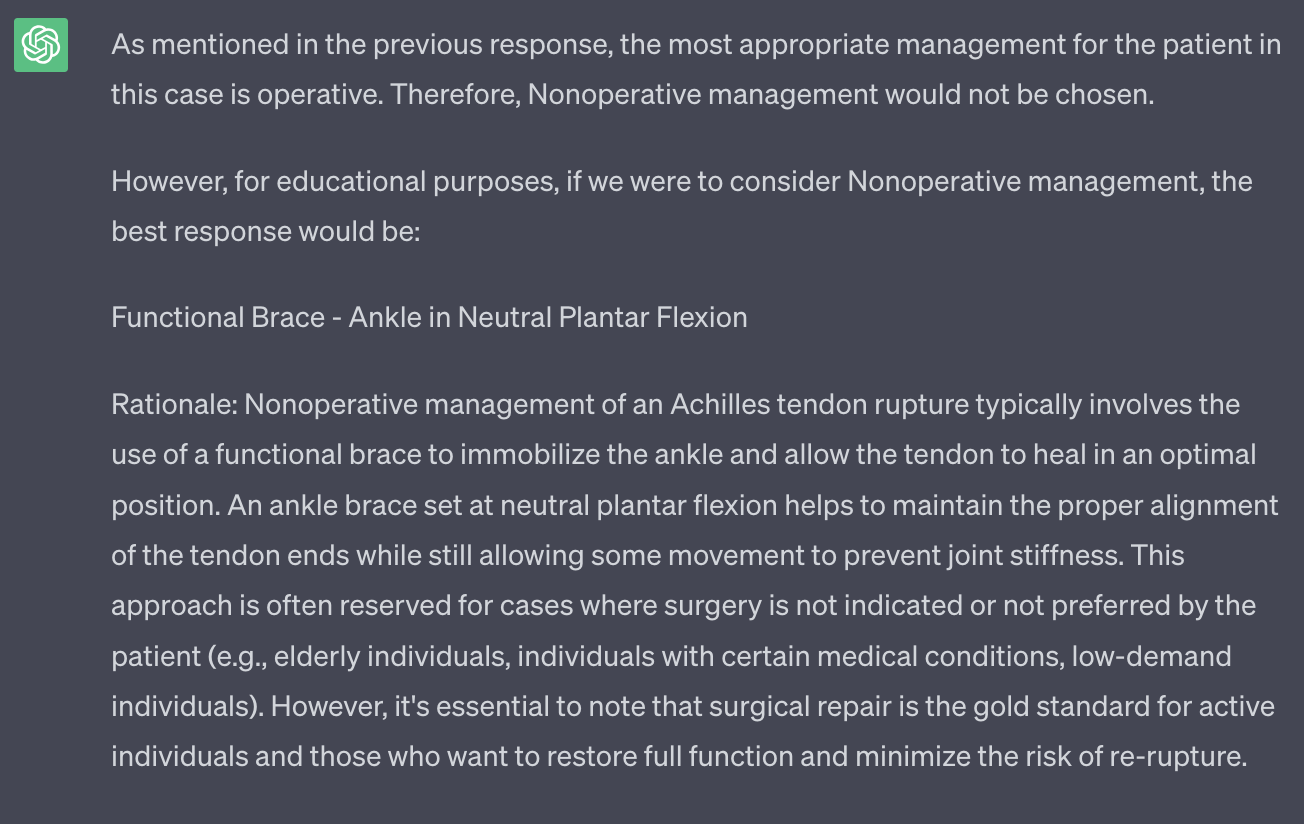


Q5: If you choose Nonoperative Management with a brace or cast, what would be your immediate weight-bearing protocol?

- I would not choose Nonoperative Management
- Full weight bearing
- TTWB
- PWB
- NWB


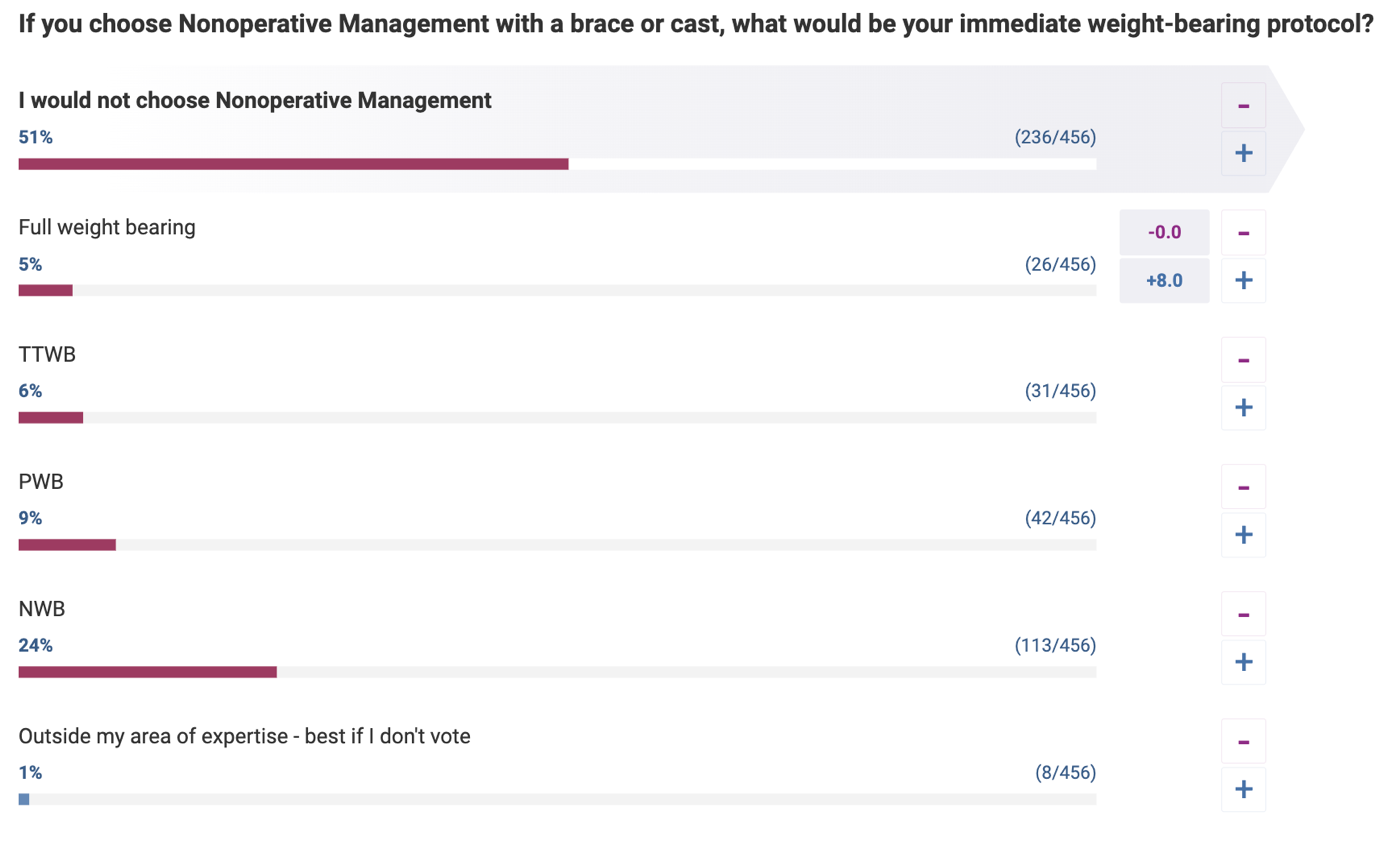


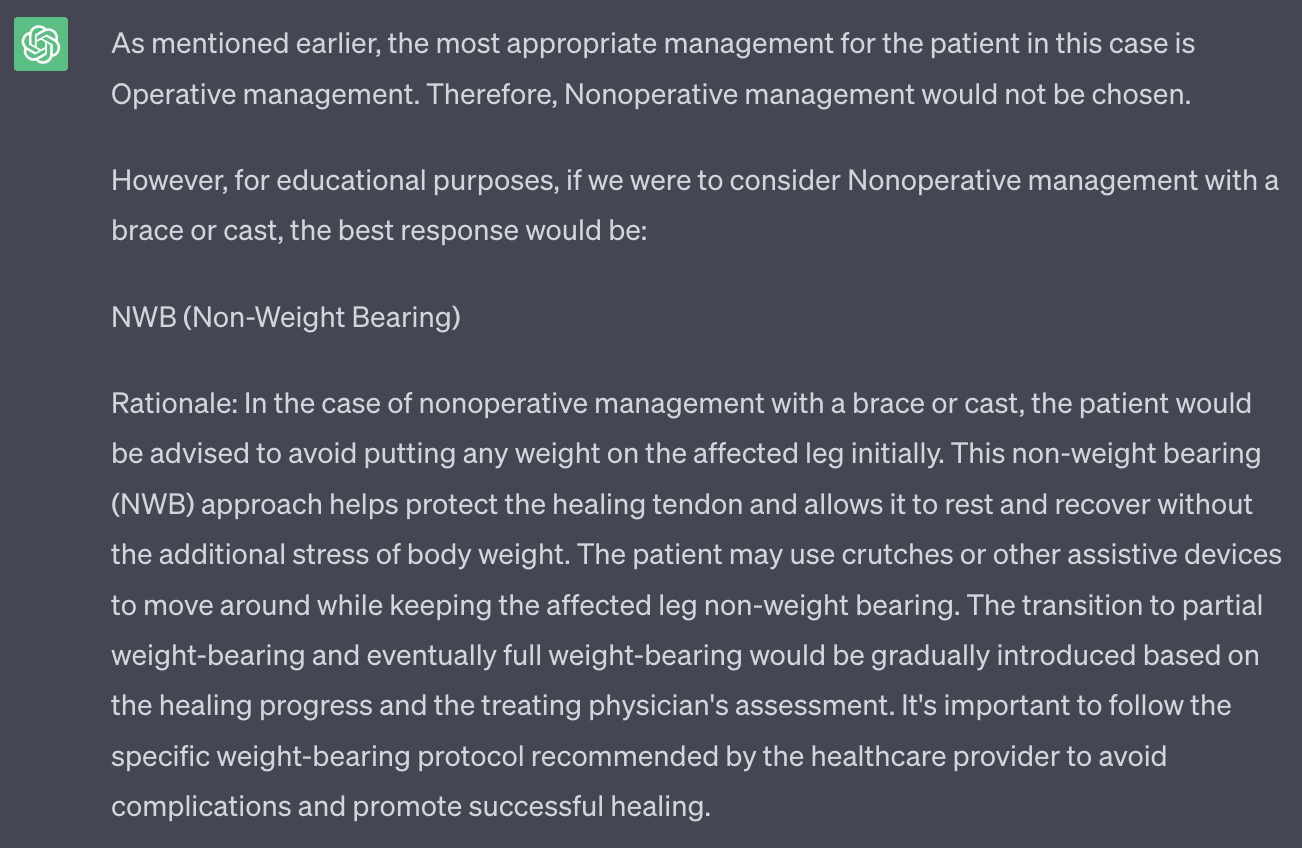


Q6: If you choose Nonoperative management, when would you begin range of motion exercises / physical therapy?

- I would not choose Nonoperative Management
- Immediately
- 1 week
- 2 weeks
- 3 weeks
- 4 weeks
- 5 weeks
- 6 weeks
- 7 weeks
- 8 weeks or more


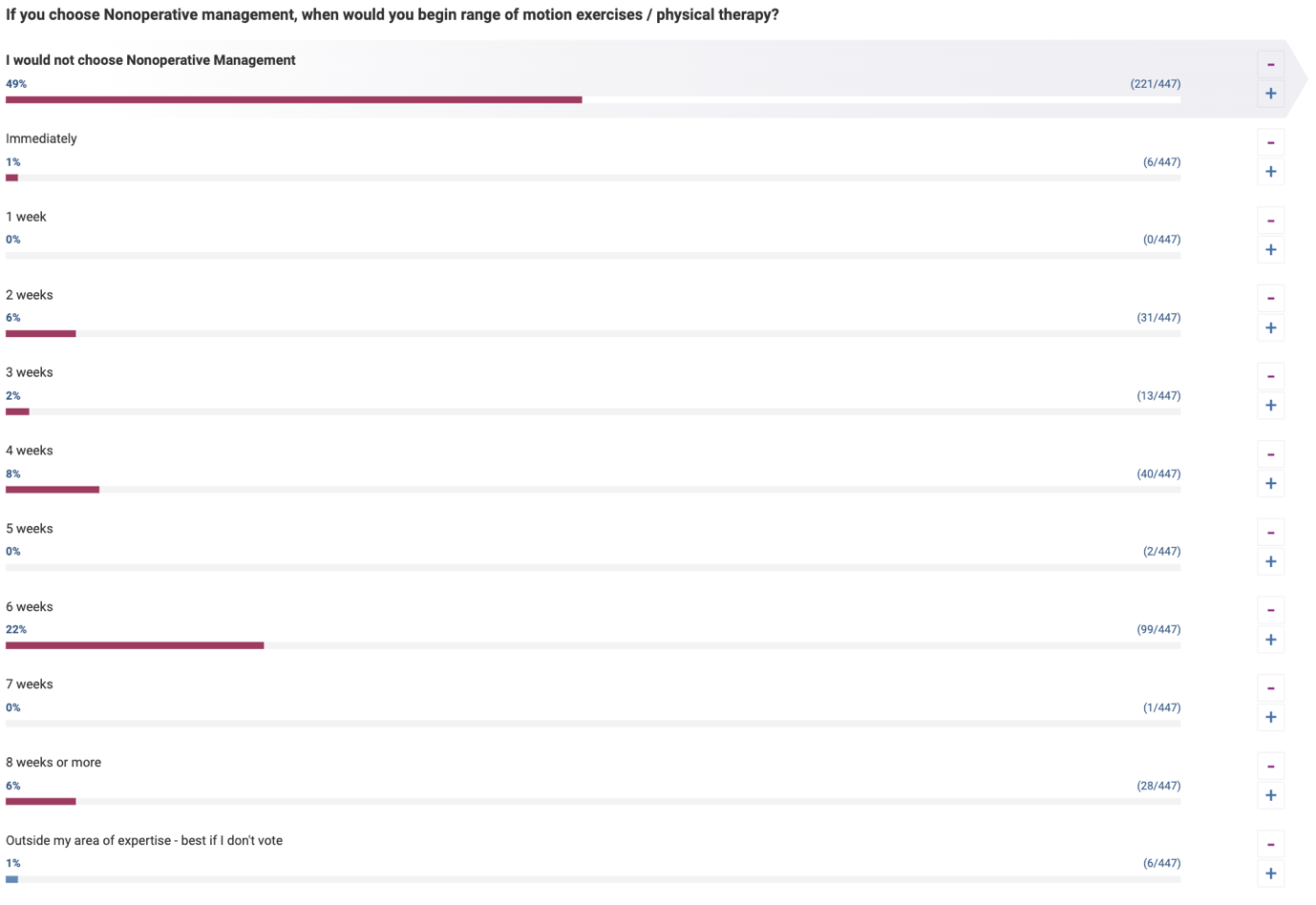


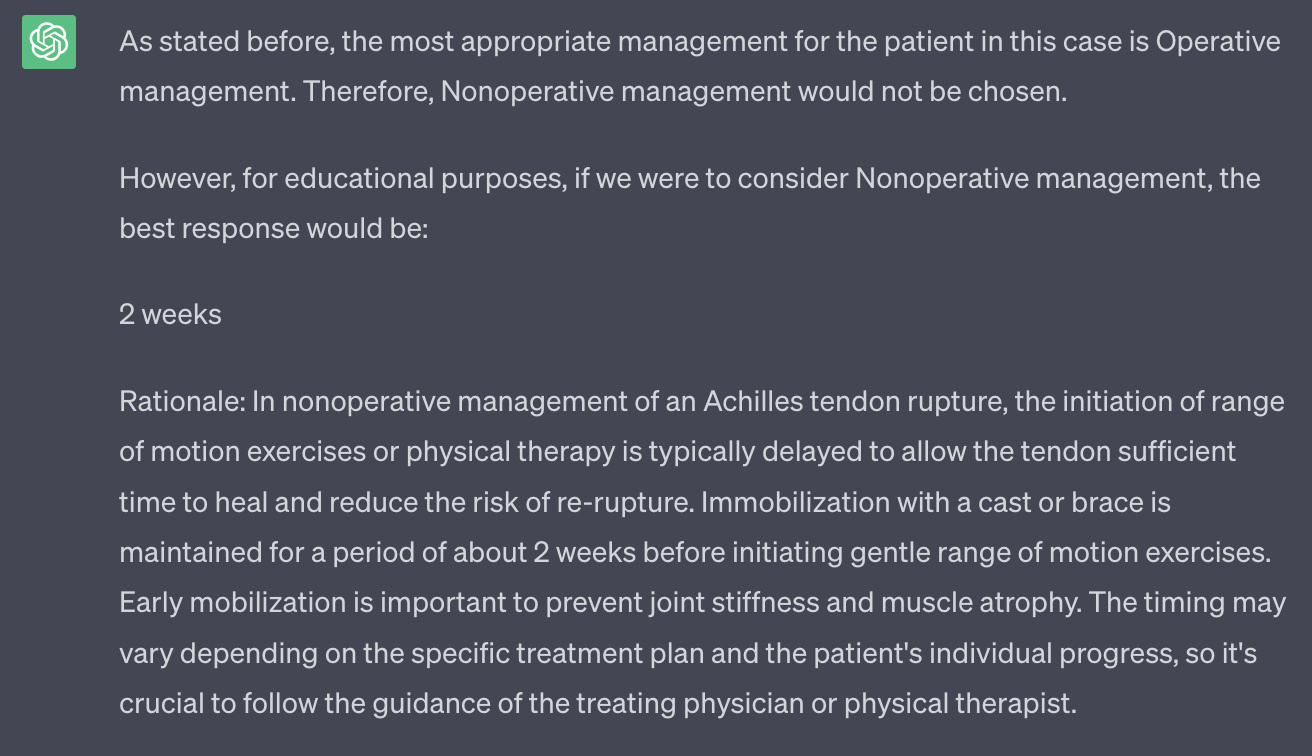


Q7: If you choose Operative Management, how would you treat this patient?

- I would not choose Operative Management
- Repair - Open Direct Alone
- Repair - Open Direct + FHL Transfer
- Repair - Percutaneous (PARS) Alone
- Repair - Percutaneous (PARS) + FHL Transfer
- Reconstruction - VY Advancement Alone
- Reconstruction - VY Advancement + FHL Transfer
- Reconstruction - Soft Tissue Graft Alone
- Reconstruction - Soft Tissue Graft + FHL Transfer


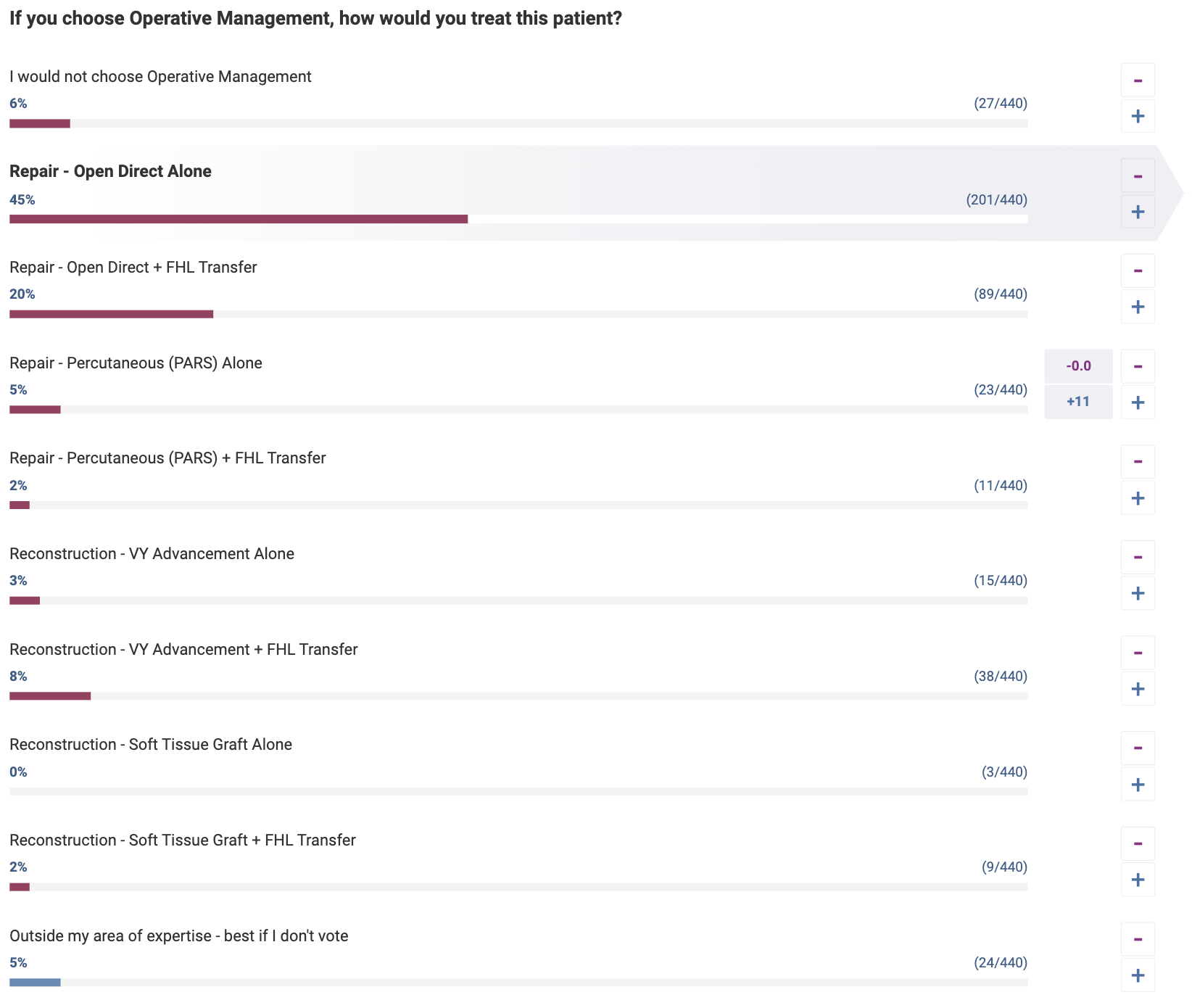


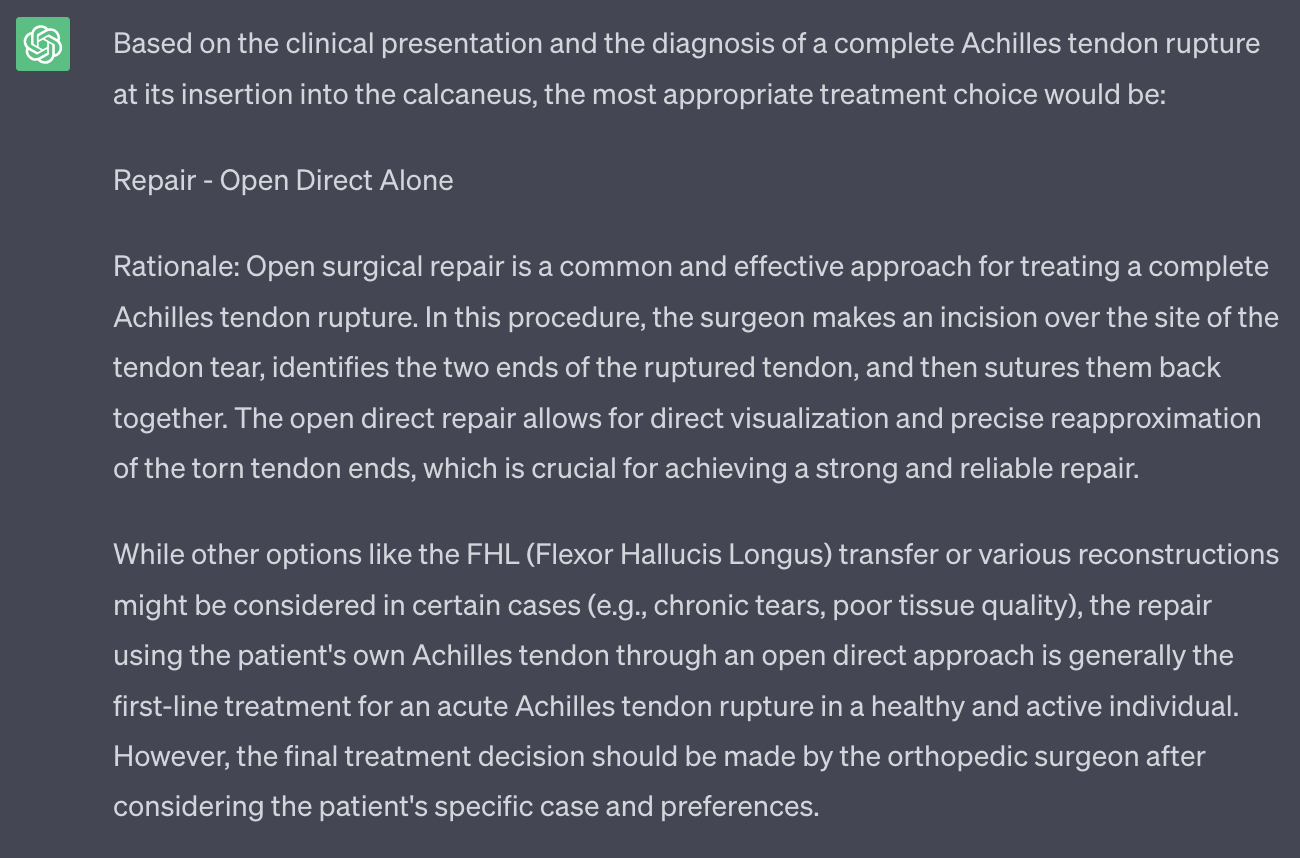


Q8: If you choose Open Direct Operative Repair, would you extend your suture fixation to the calcaneus?

- I would not choose Open Direct Operative Repair
- No, I would perform end-to-end soft tissue repair Alone
- Yes - with osseous Bone Tunnels
- Yes - with Suture Anchors
- Yes - with Cortical Buttons
- Yes - with other bony fixation method


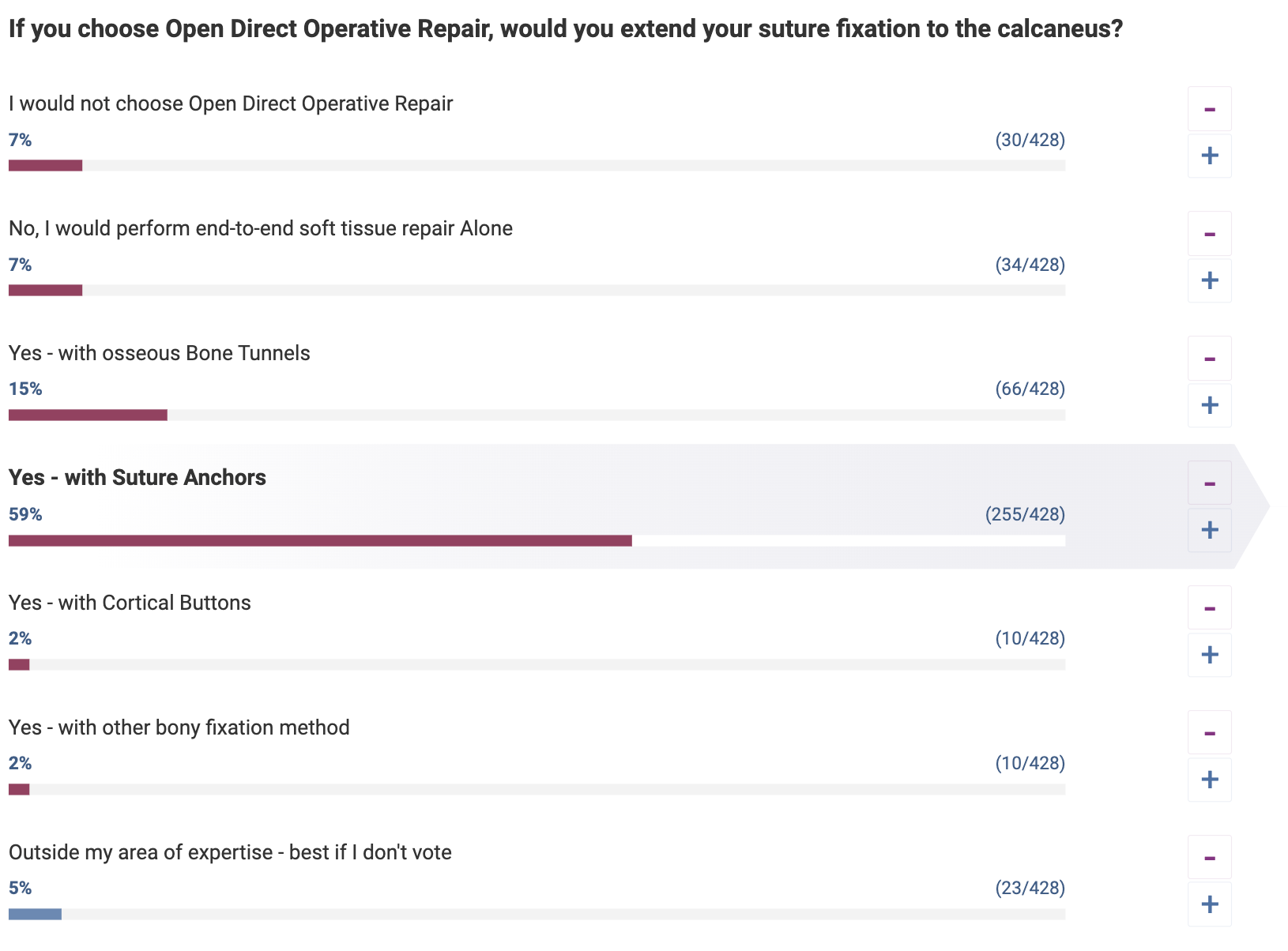


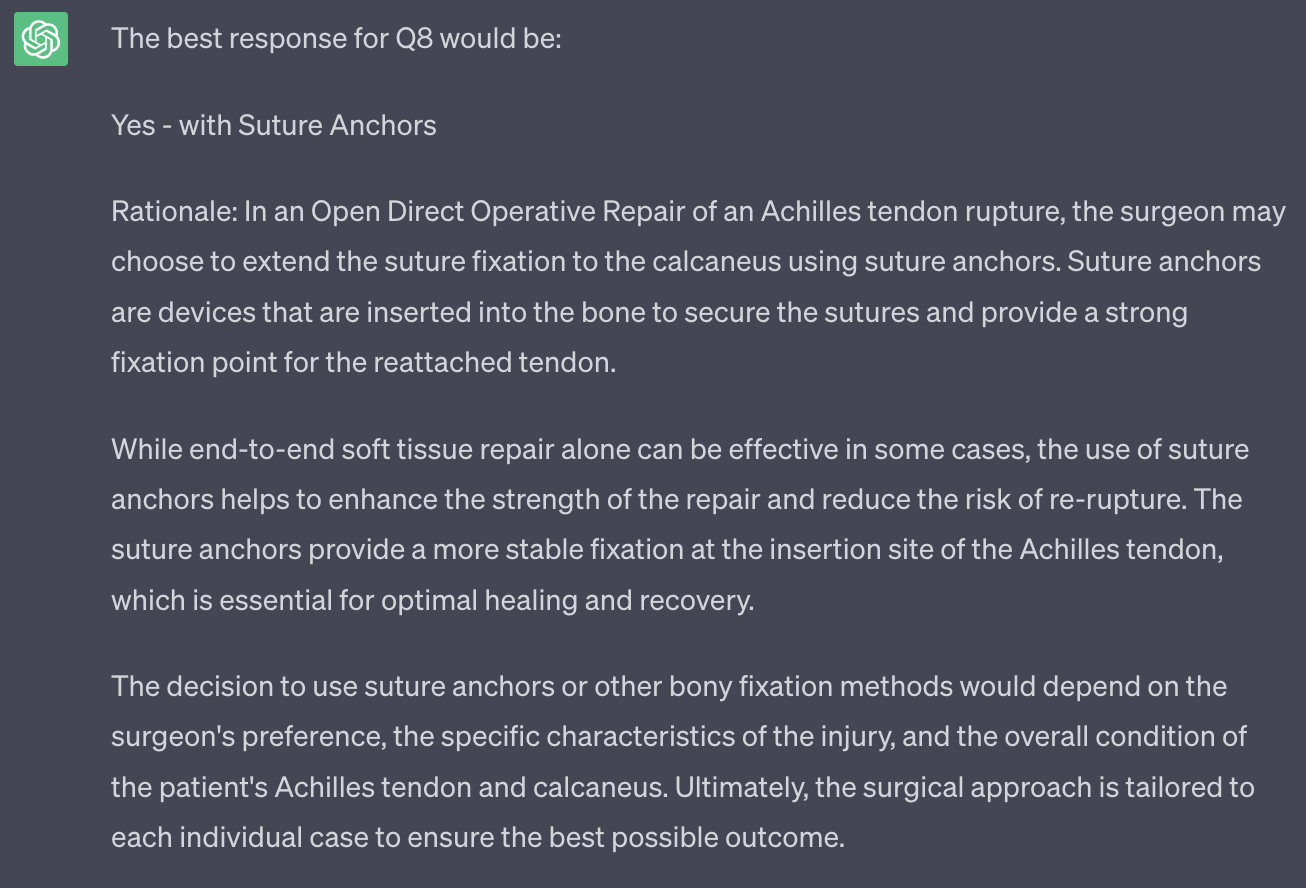


Q9: If you choose Operative Management, would you augment your repair or reconstruction with biologics?

- I would not choose Operative Management
- No, I would not augment with biologics
- Yes – PRP
- Yes - Other Biologics


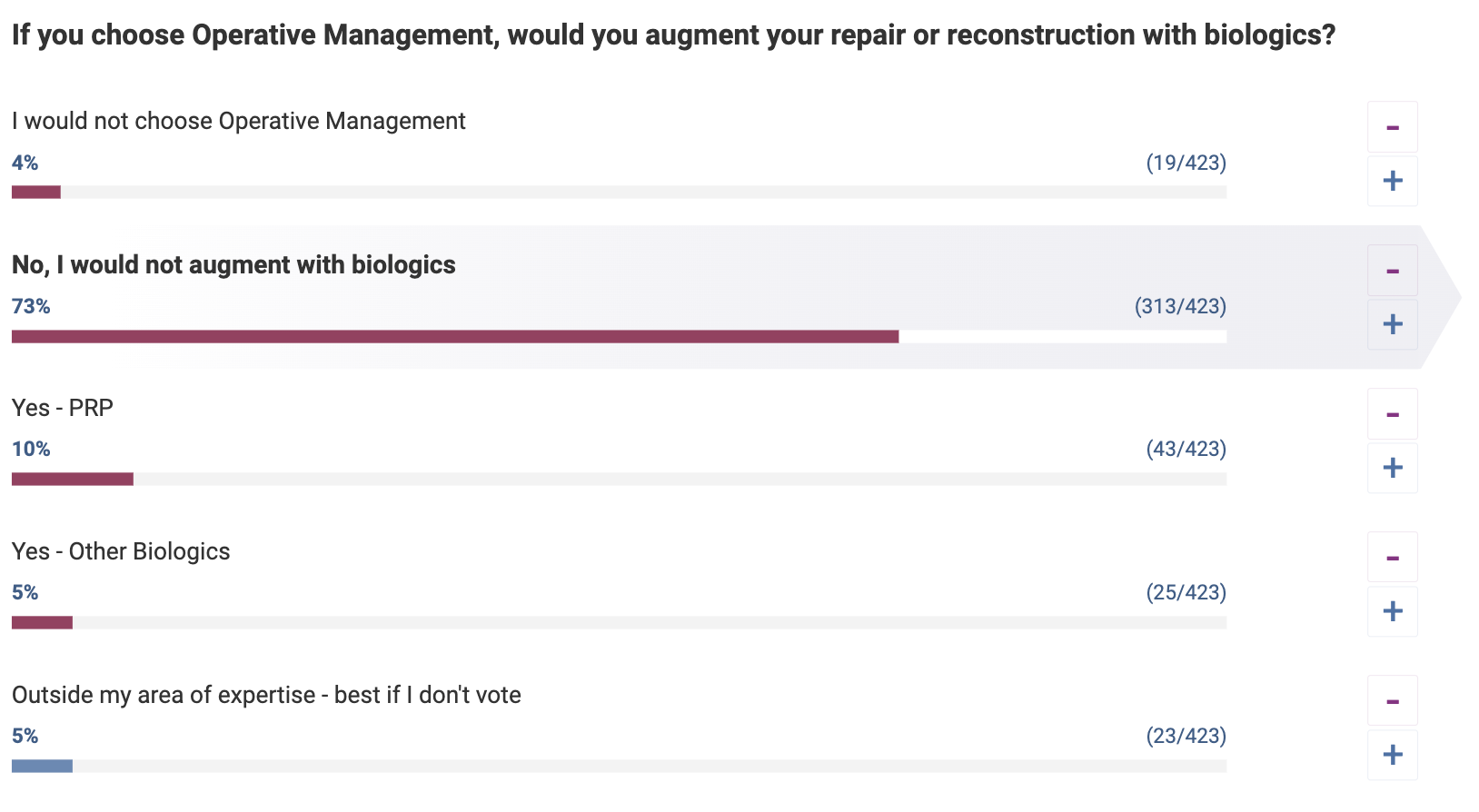


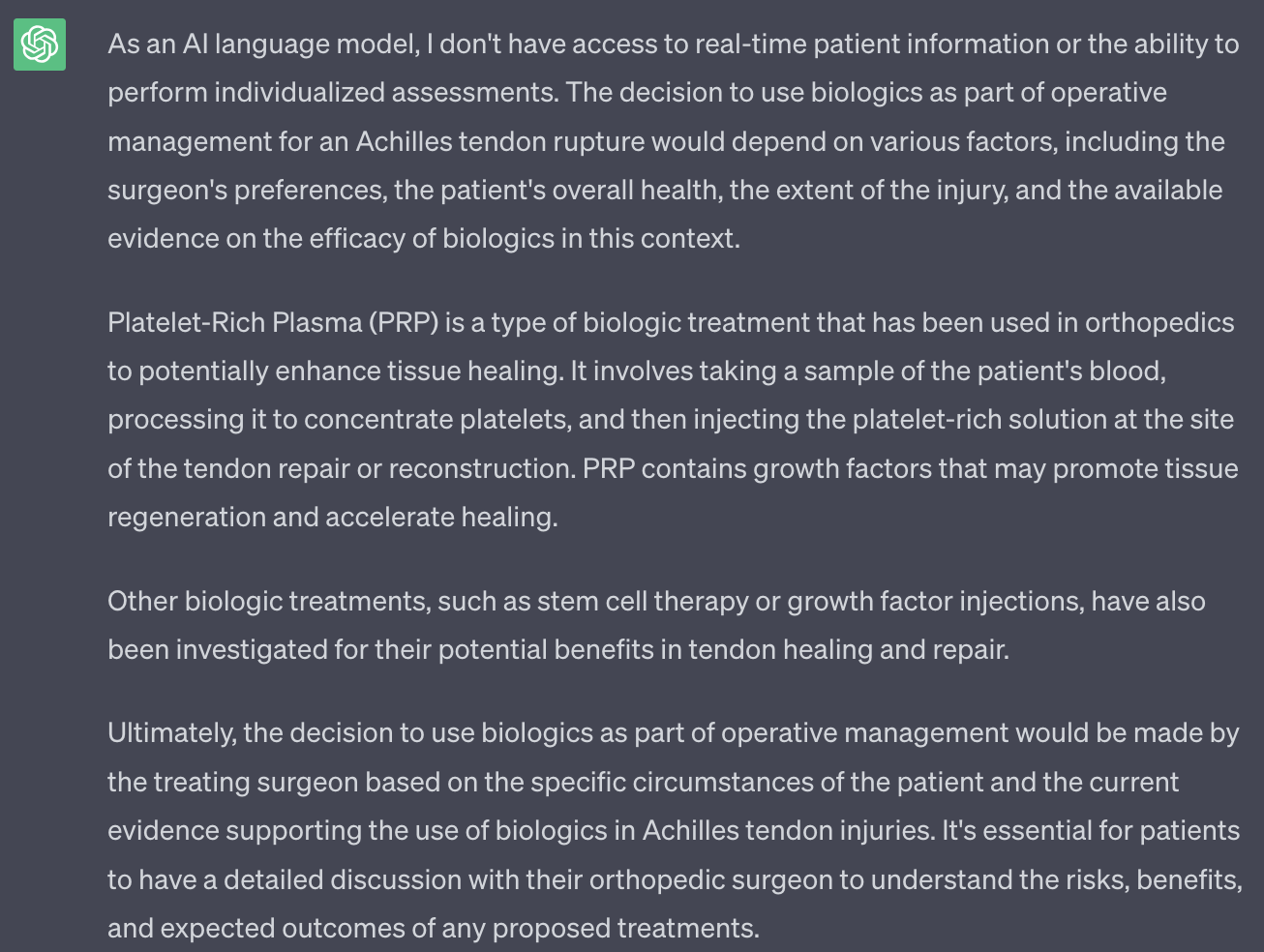


Q10: If choosing Nonoperative treatment, what DVT prophylaxis would you prescribe?

- I would not choose nonoperative treatment.
- Aspirin
- Lovenox
- Xarelto
- Coumadin
- I would not prescribe DVT prophylaxis


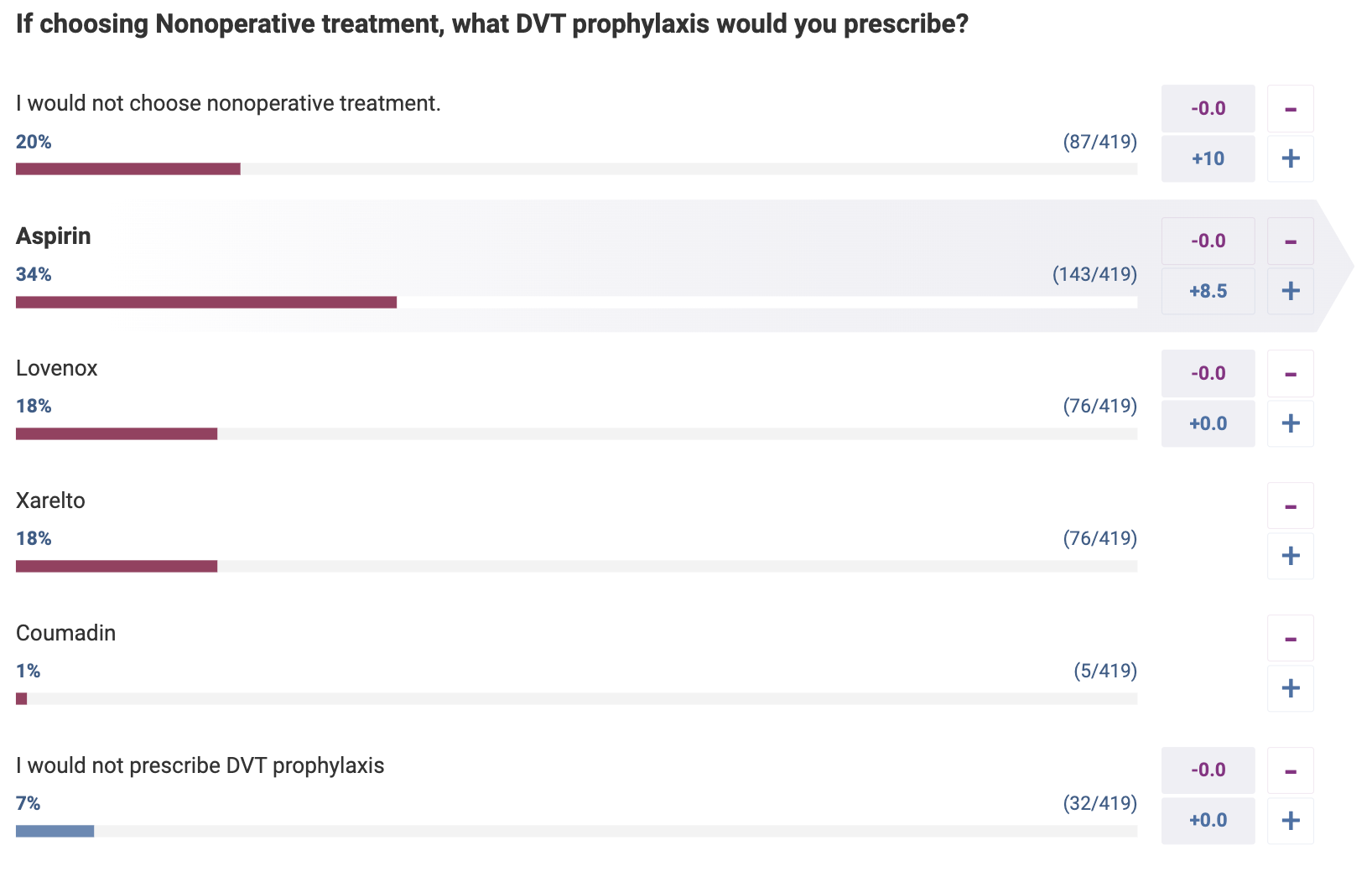


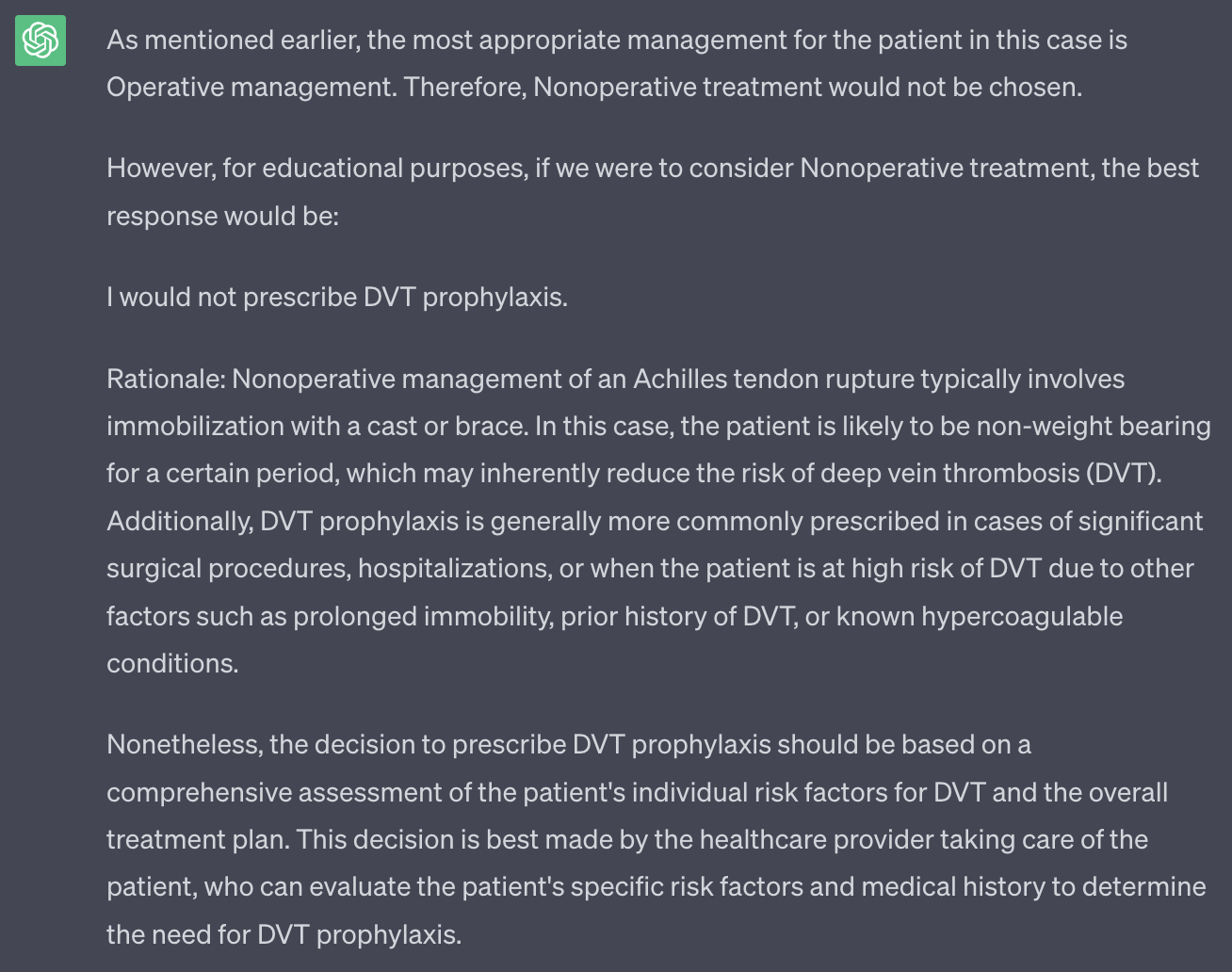


Q11: If choosing Operative treatment, what DVT prophylaxis would you prescribe?

- I would not choose nonoperative treatment.
- Aspirin
- Lovenox
- Xarelto
- Coumadin
- I would not prescribe DVT prophylaxis


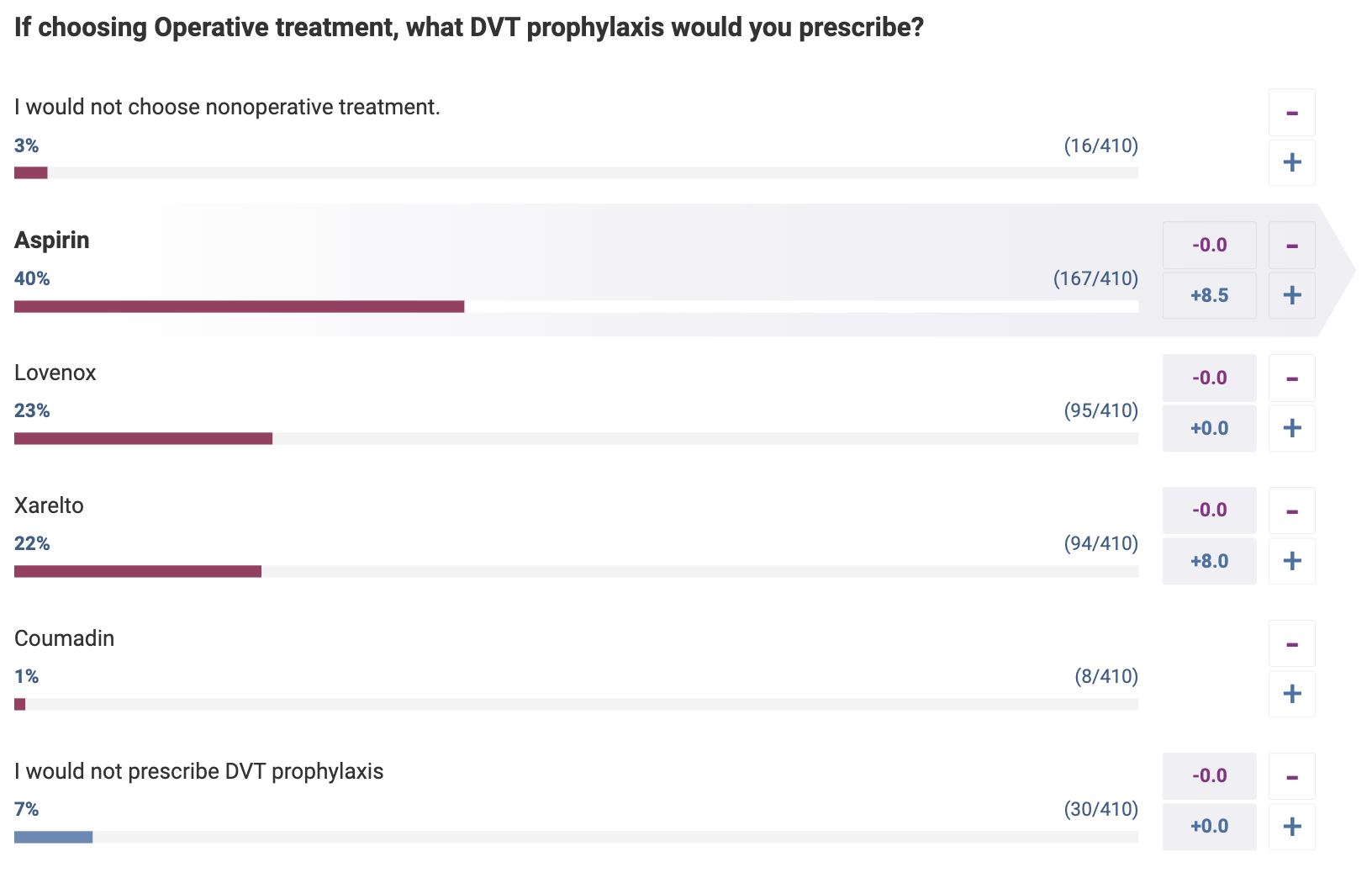


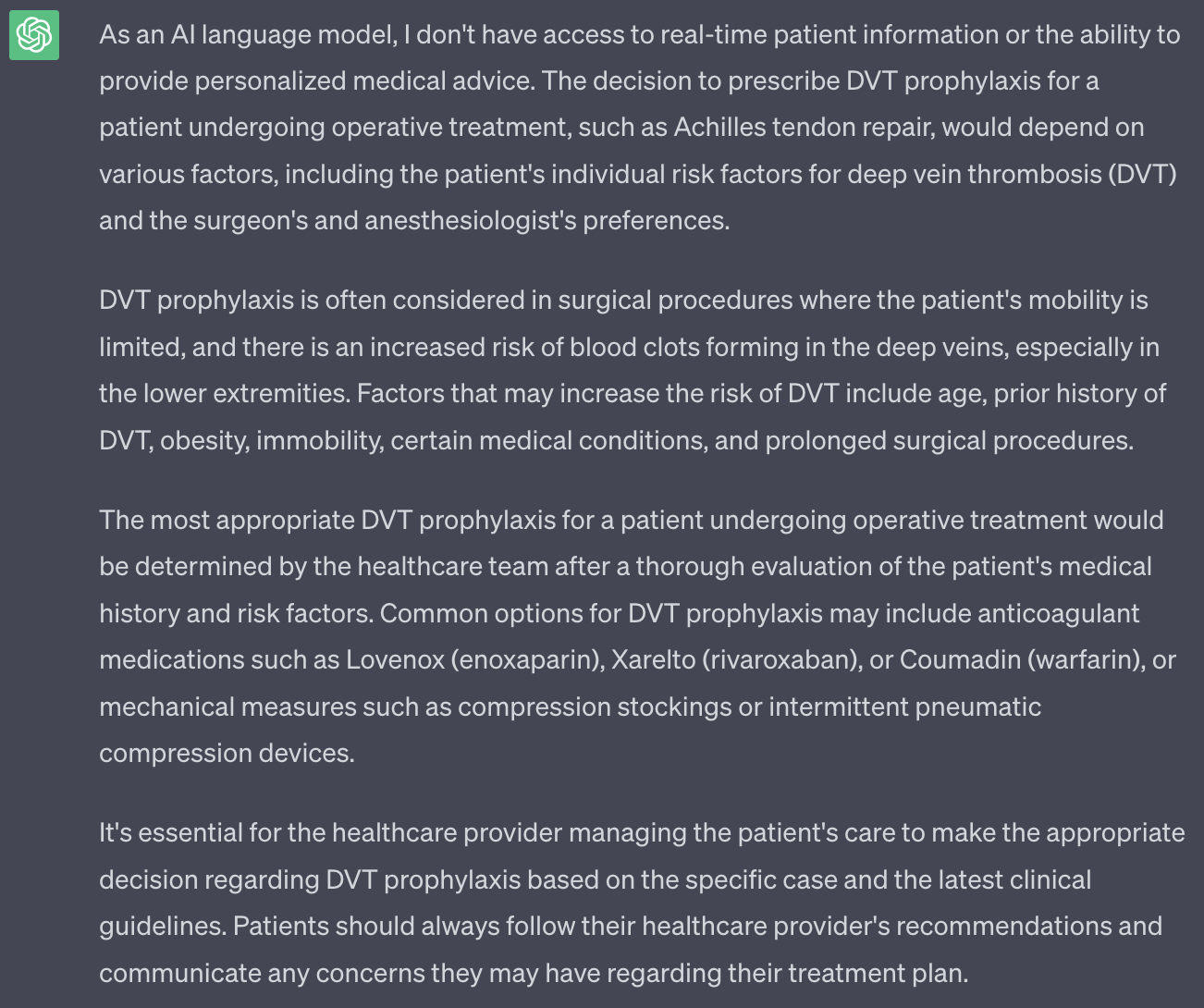


Q12: I you choose Operation Management and performed the operation shown (Achilles repaired using Arthrex speed bridge), when would you begin range of motion / physical therapy?


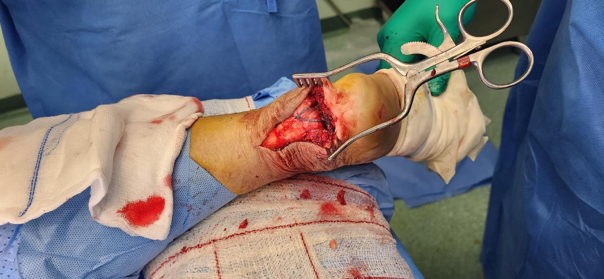

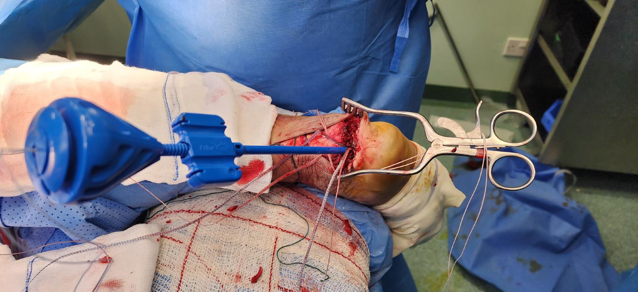

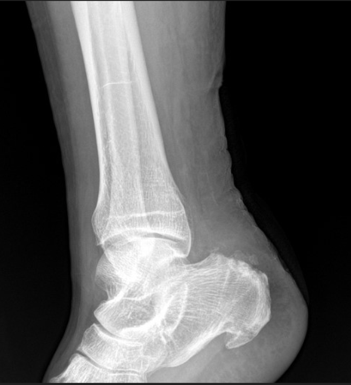
[images not provided to ChatGPT]

- I would not choose Operative Management
- Immediately
- 1 week
- 2 weeks
- 3 weeks
- 4 weeks
- 5 weeks
- 6 weeks
- 7 weeks
- 8 weeks or more


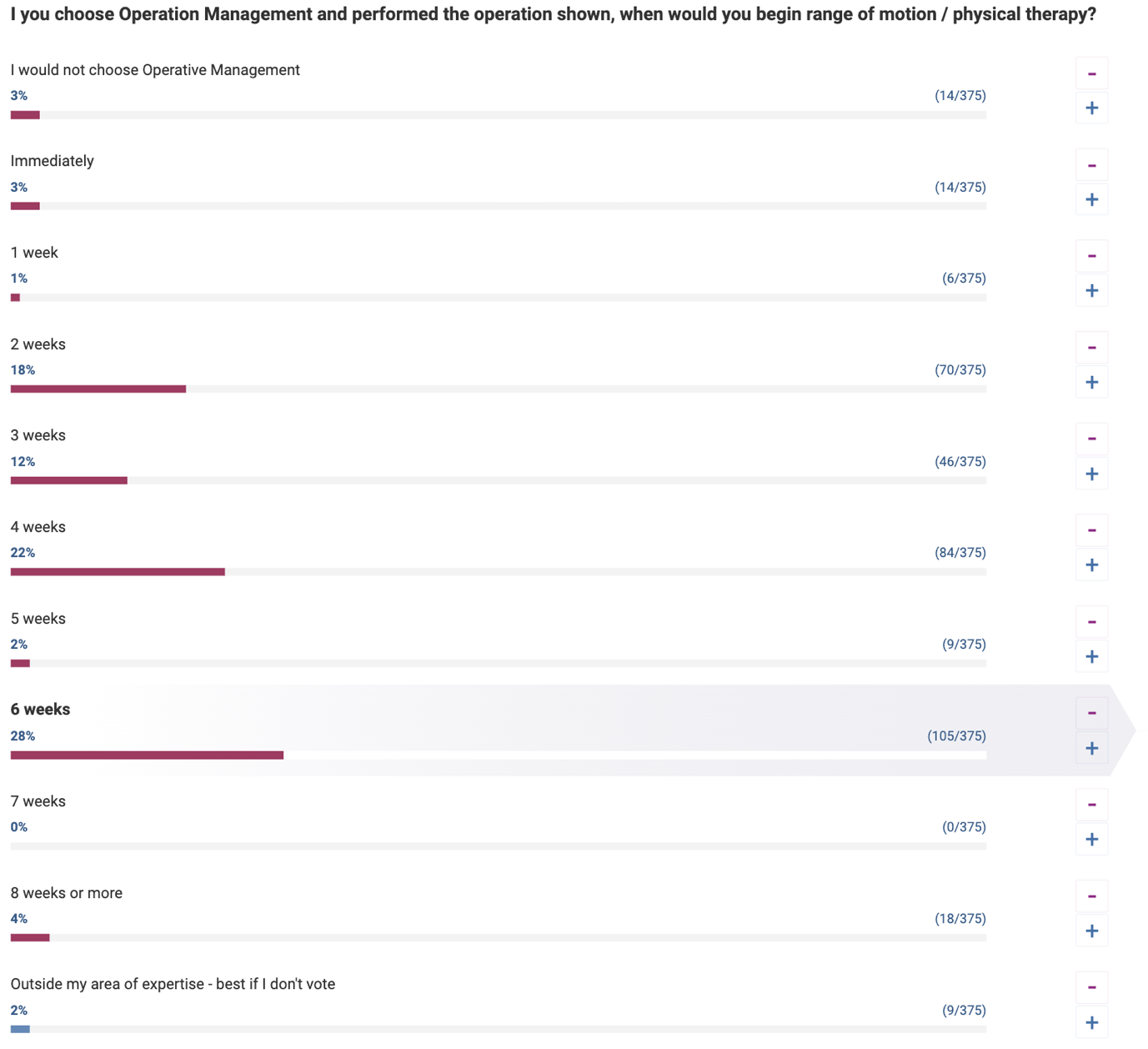


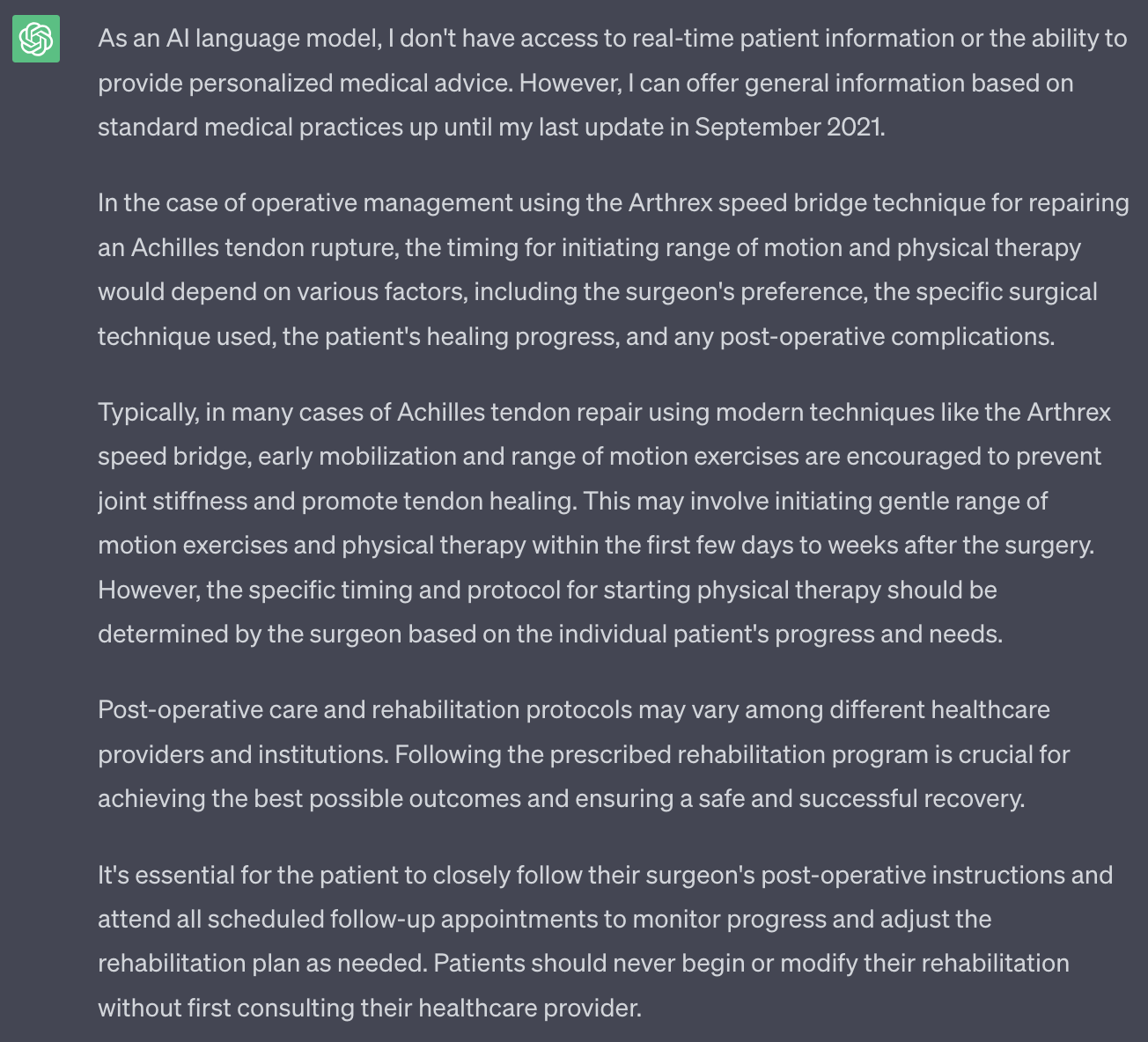


# Title: 5th Metacarpal Fracture in 22M

Category: Hand

Popularity: 1

Date: 20230725

Link: <https://www.orthobullets.com/Site/Cases/View/663b70e7-4dd5-4515-8d38-bfa51e17b2a7>

Images:
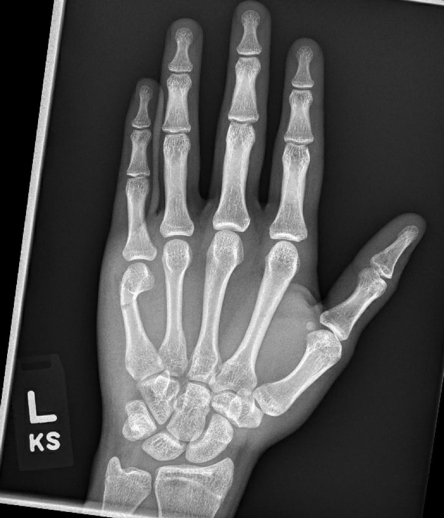

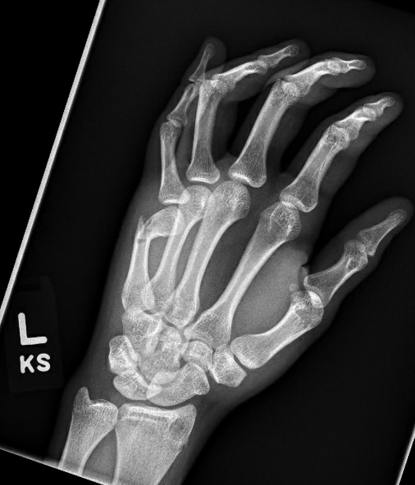

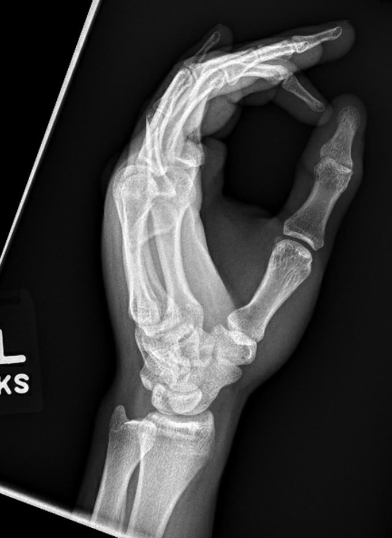


ChatGPT-3.5 dialogue:
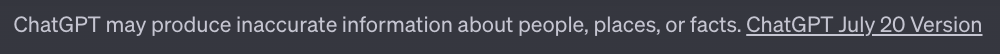


I am going to provide you a clinical vignette. There will be a series of 10 questions to follow. Please choose the single best response for each question.

History of presenting incident:

A 22-year-old male presents to the Emergency Department for evaluation of a left hand injury he sustained after punching a car windshield two days prior. He localizes the pain to the ulnar border of his hand, and rates it 7/10 in severity. The pain is worse with movement of the hand, and only mildly present at rest. He denies any other injuries.

Past medical history:

Non-contributory

Physical exam:

Visual inspection of the hand reveals ecchymosis overlying the 5th metacarpal without any open wounds. Palpation of the 5th metacarpal shaft illicit pain. There is rotational malalignment of the 5th digit radially within the finger cascade. Motor function is grossly intact as demonstrated by the cardinal hand movements. Sensation is intact to all distributions of the hand. The radial pulse is palpable, and there is brisk cap refill in all the fingers.

Imaging findings:

Plain AP, lateral and oblique x-ray radiographs of the left hand show an isolated 5^th^ metacarpal neck fracture, with approximately 45 degrees of dorsal angulation.

Q1: In addition to the plain film radiographs shown, would you obtain other imaging to guide management?

- No - current radiographs are sufficient
- Yes - additional radiographic views (XR)
- Yes - CT scan of the hand (CT)
- Yes - MRI scan of the hand (MRI)
- Yes - XR + CT
- Yes - XR + MRI
- Yes - CT + MRI
- Yes - XR + CT + MRI


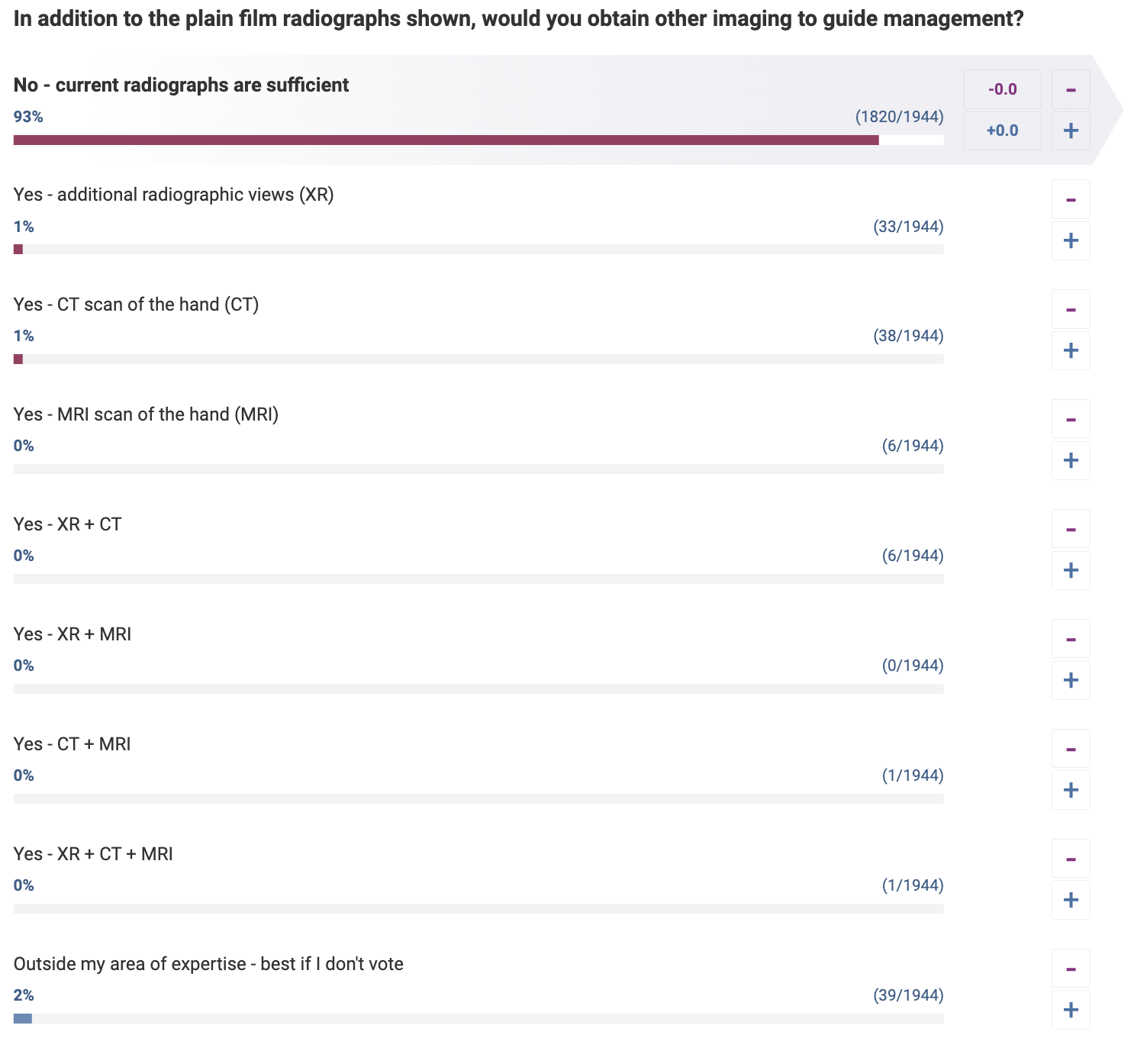


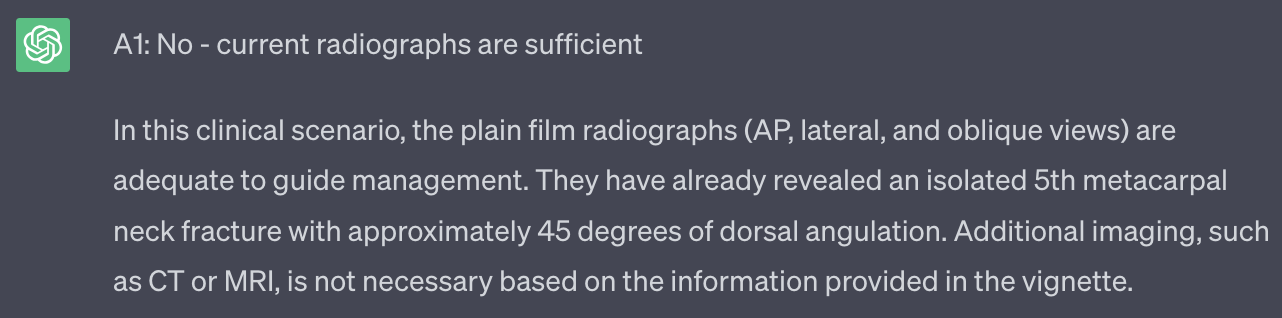


Q2: How would you manage this patient?

- Nonoperative
- Operative


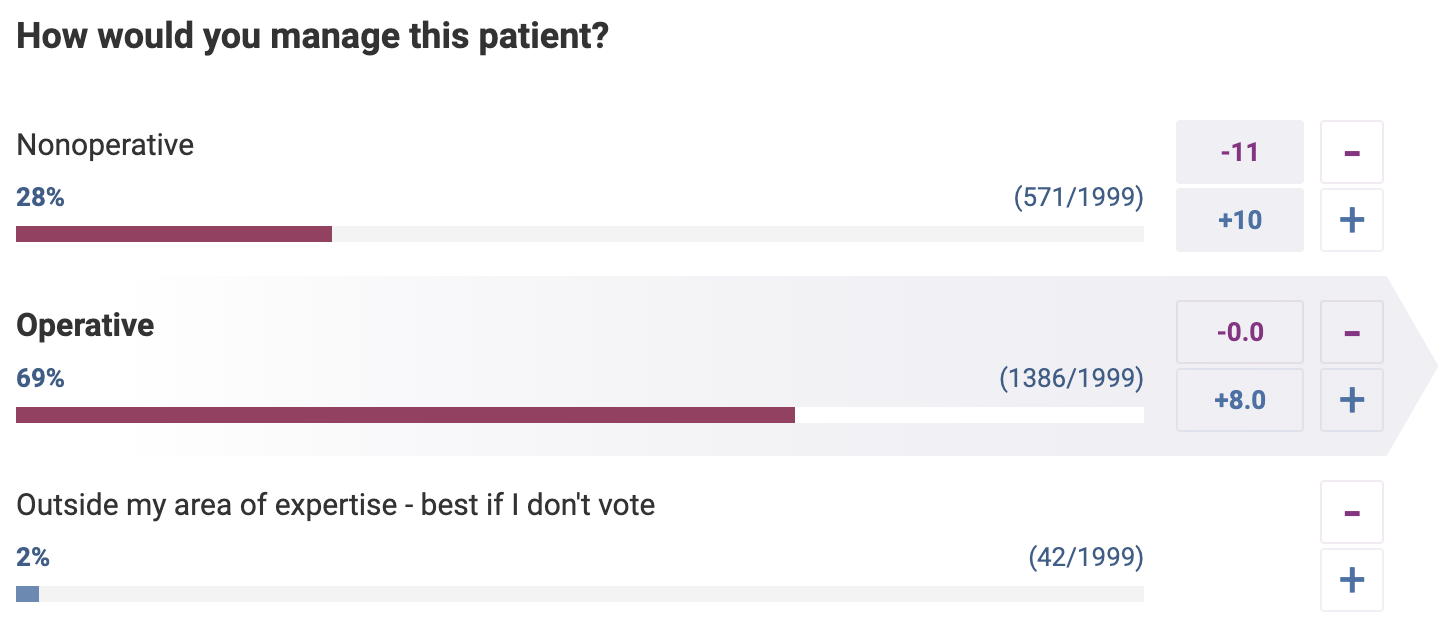


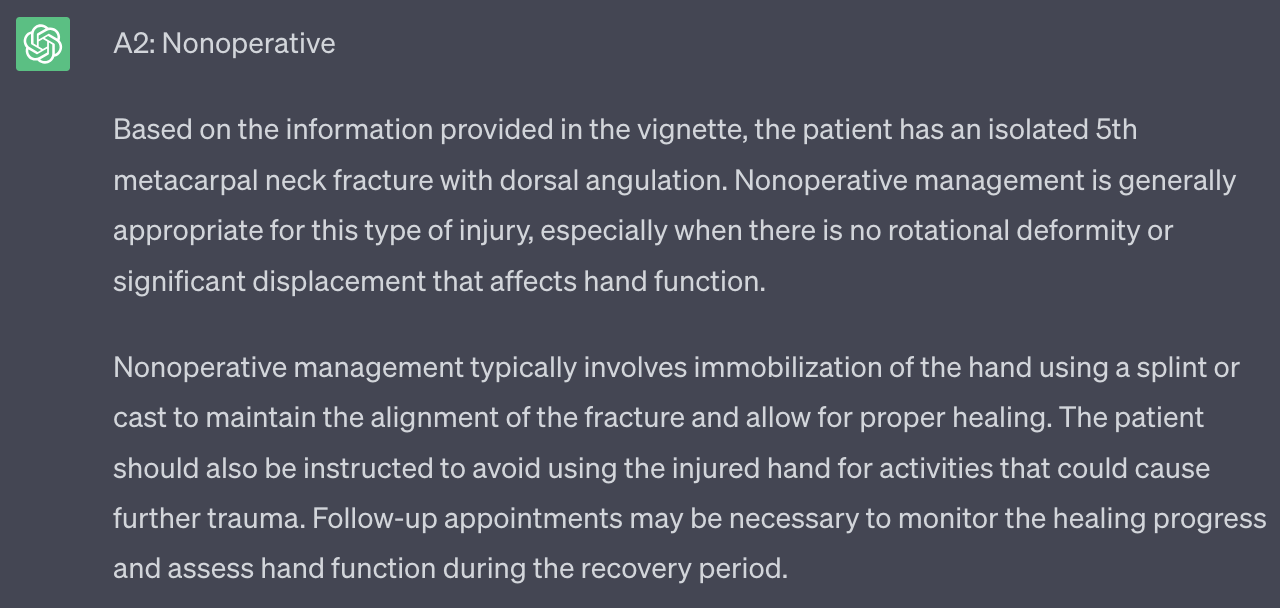


Q3: If you choose Nonoperative management, how would you immobilize the patient?

- I would not choose Nonoperative management
- Soft dressings only (no immobilization)
- Removable ulnar gutter splint/brace
- Ulnar gutter cast
- Other


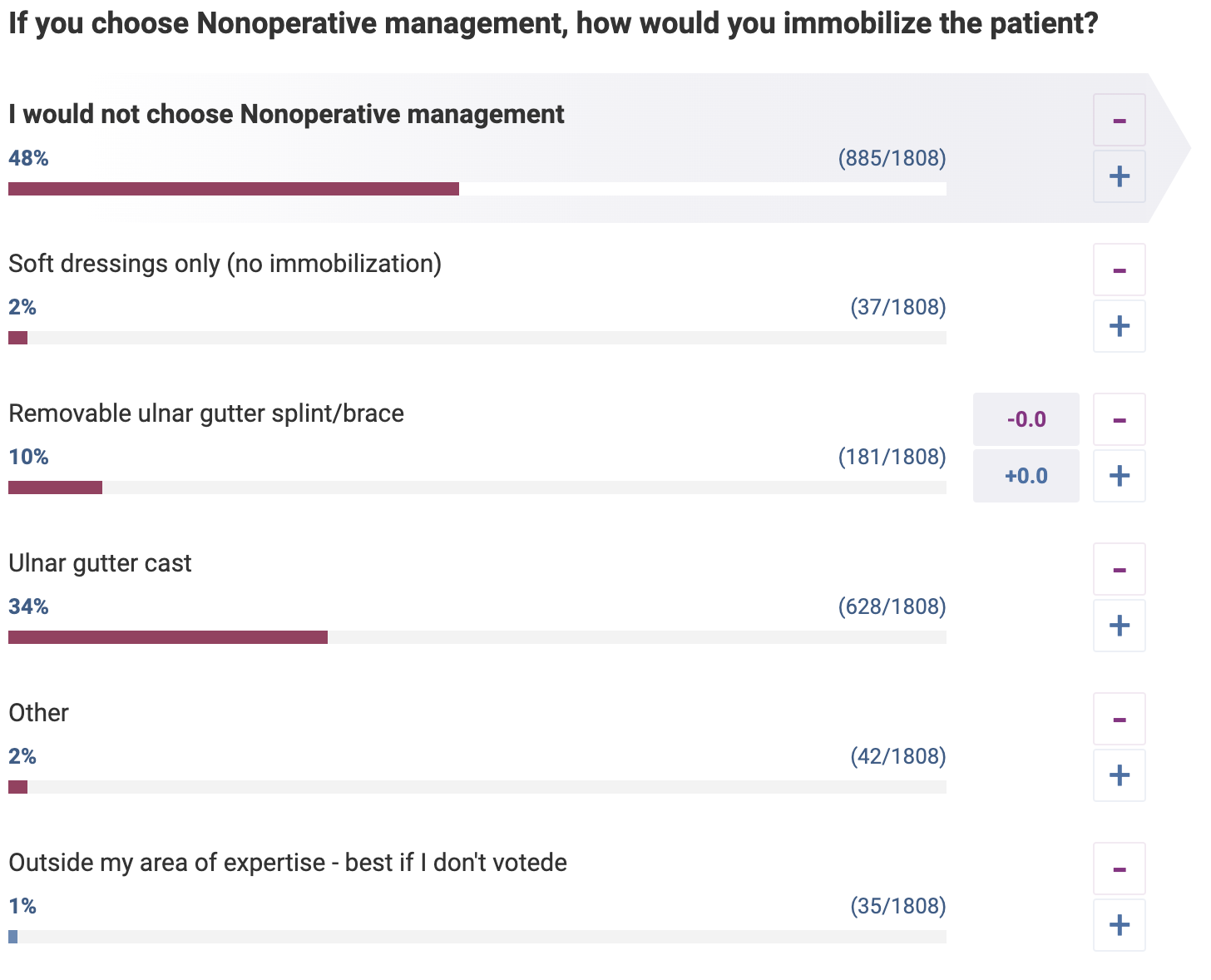


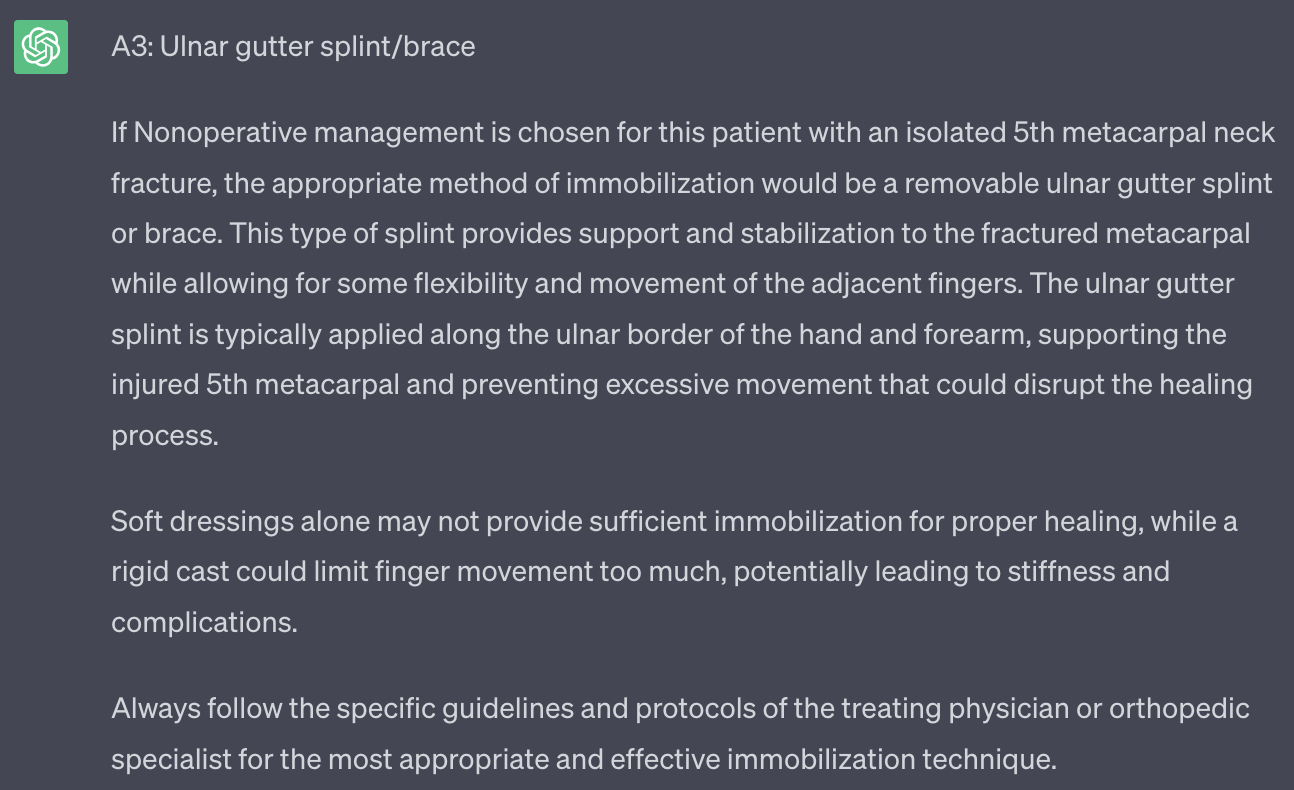


Q4: If you choose Operative management, which technique would you utilize?

- I would not choose Operative management
- External fixation only (EF)
- Planned open reduction internal fixation (ORIF)
- Closed reduction percutaneous fixation (CRPF, includes IM nail)


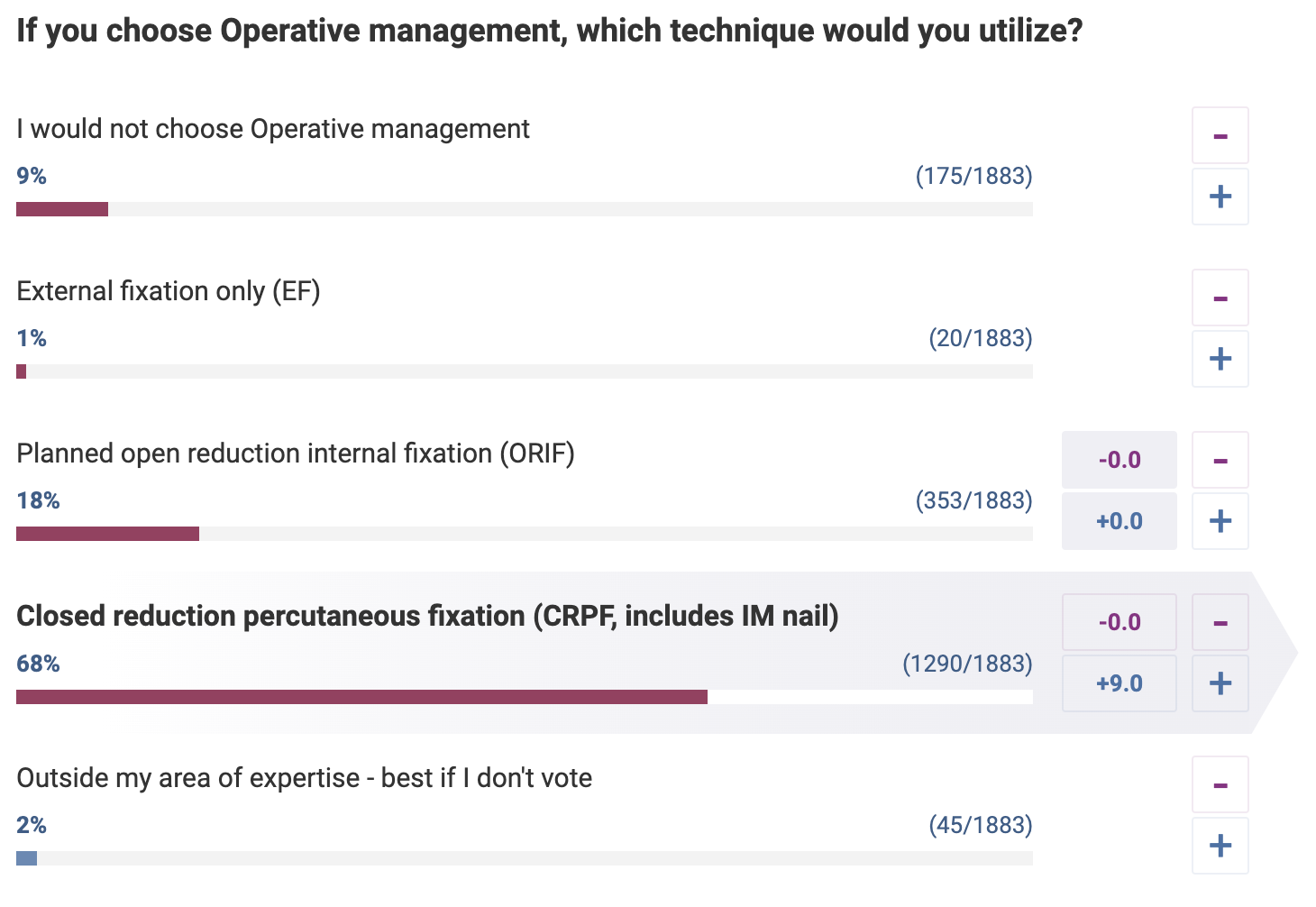


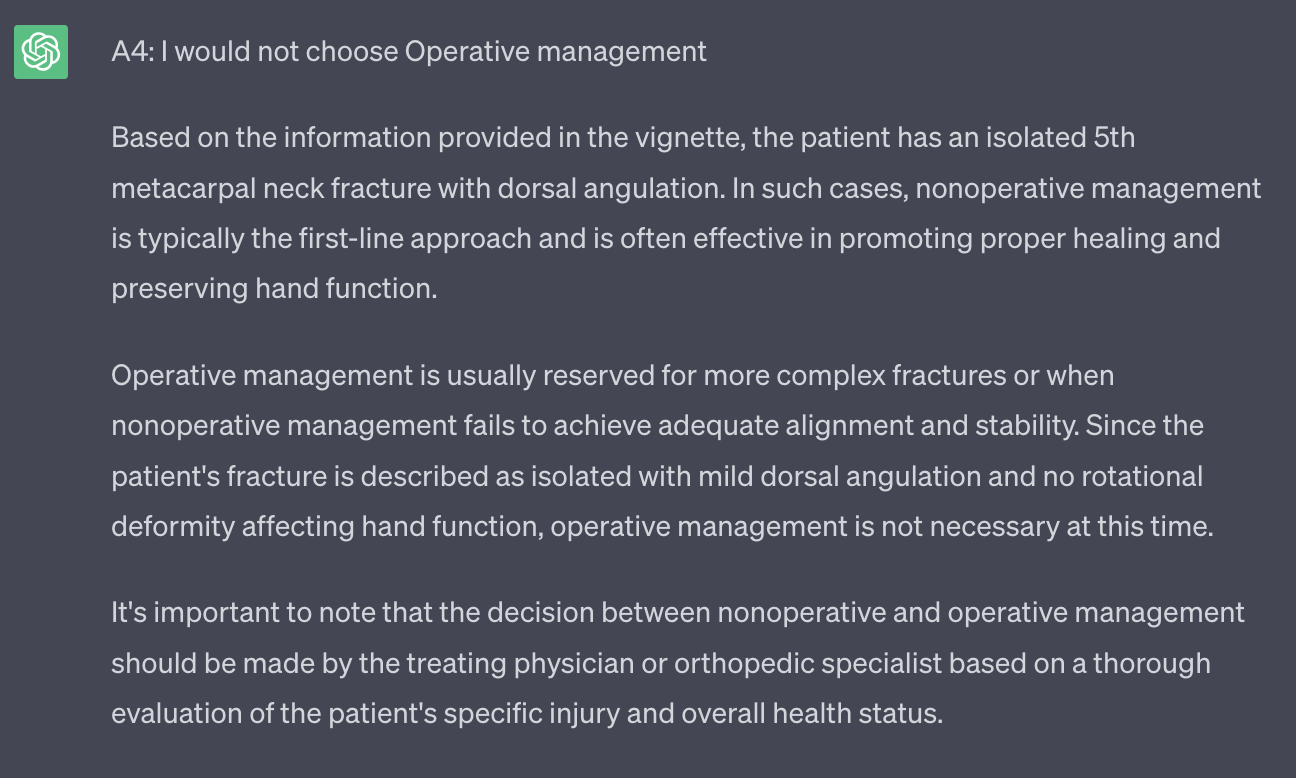


Q5: If you choose Closed reduction percutaneous fixation (CRPF), which implant would you choose?

- I would not choose CRPF
- K-wire(s)
- Screw
- Intramedullary nail


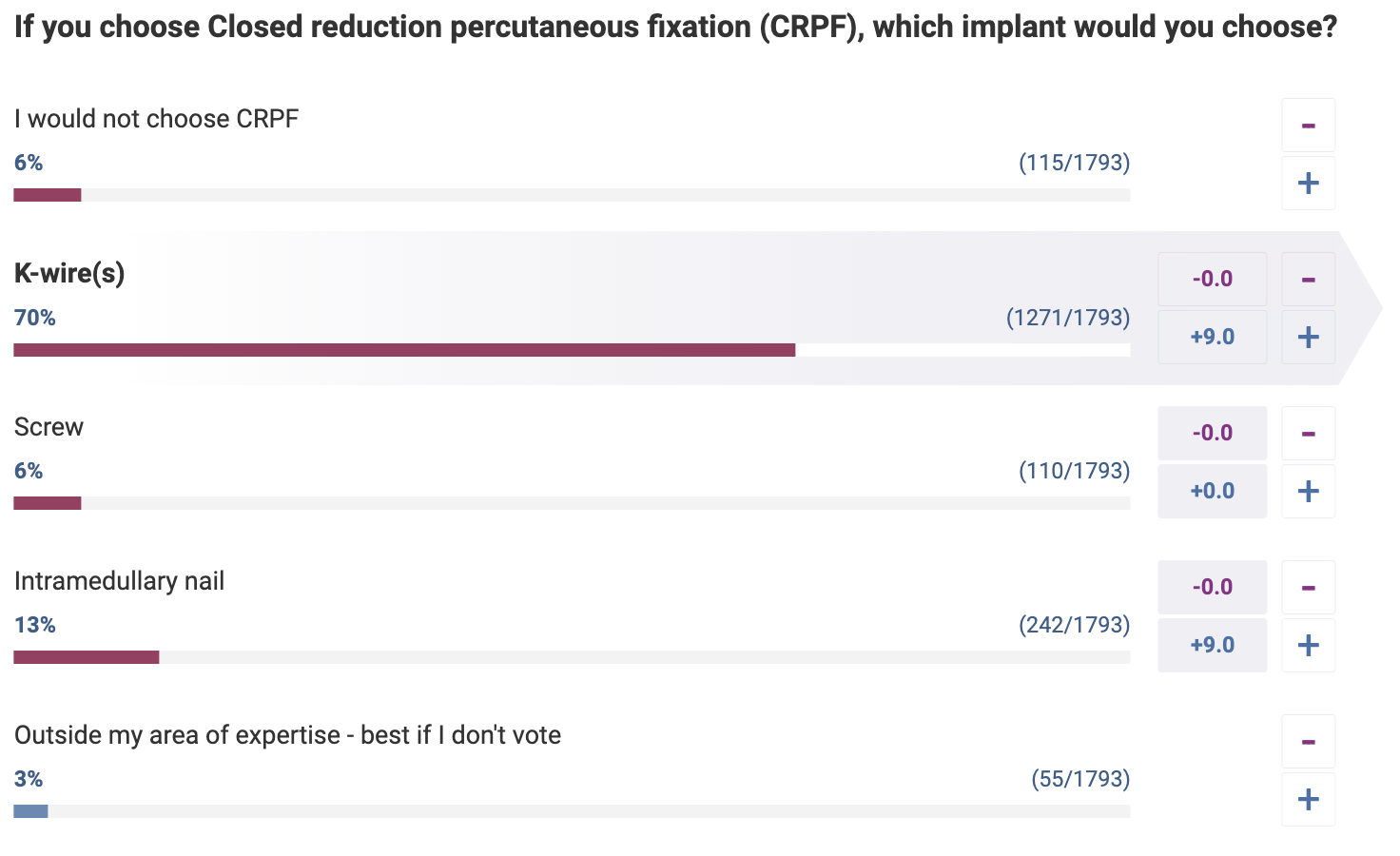


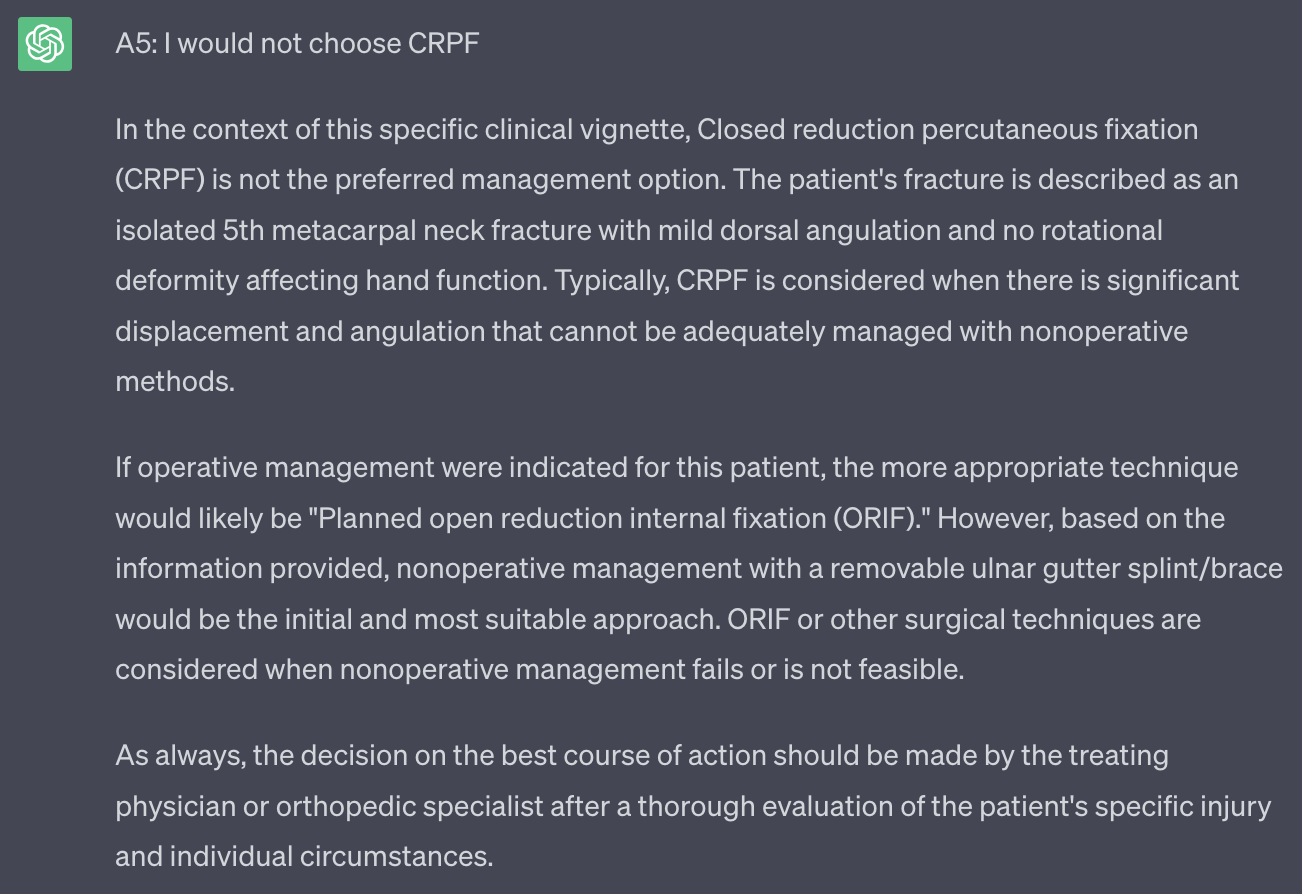


Q6: If you choose Operative management, would you perform a provisional reduction in the emergency department?

- I would not choose Operative management
- Yes
- No


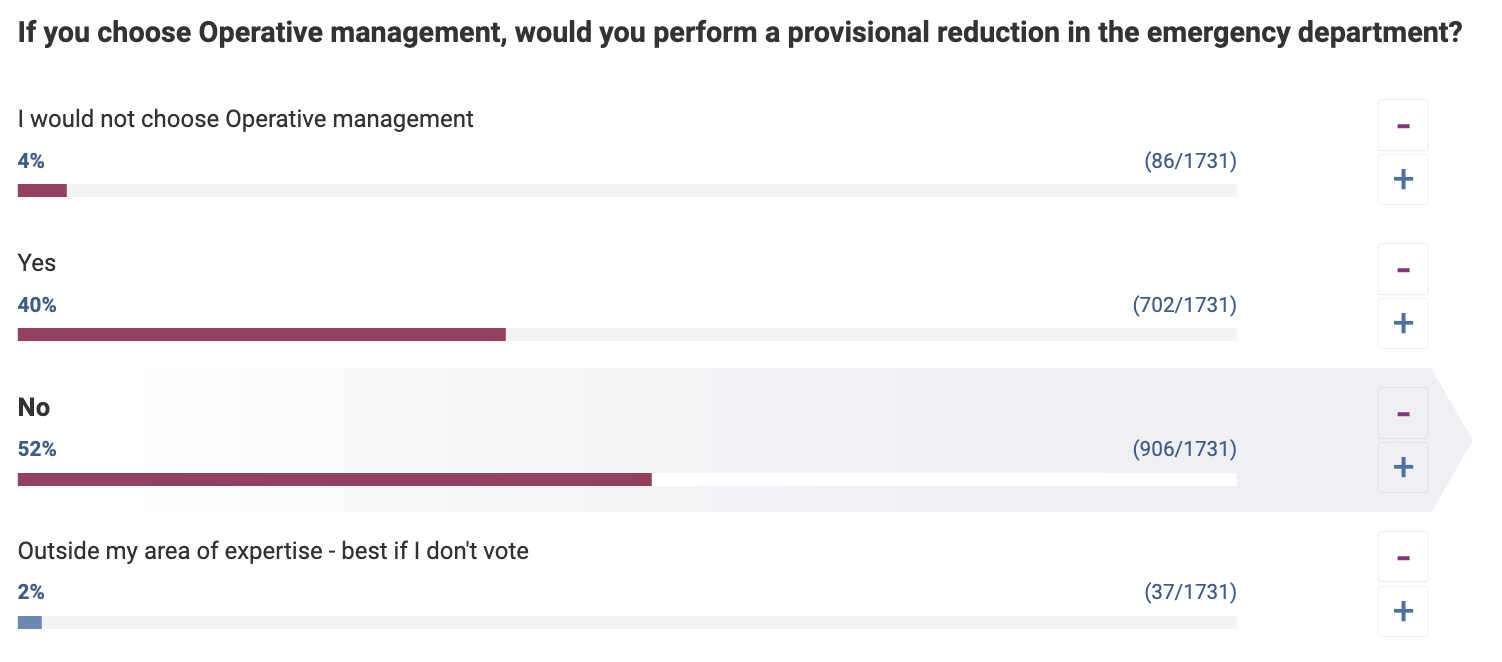


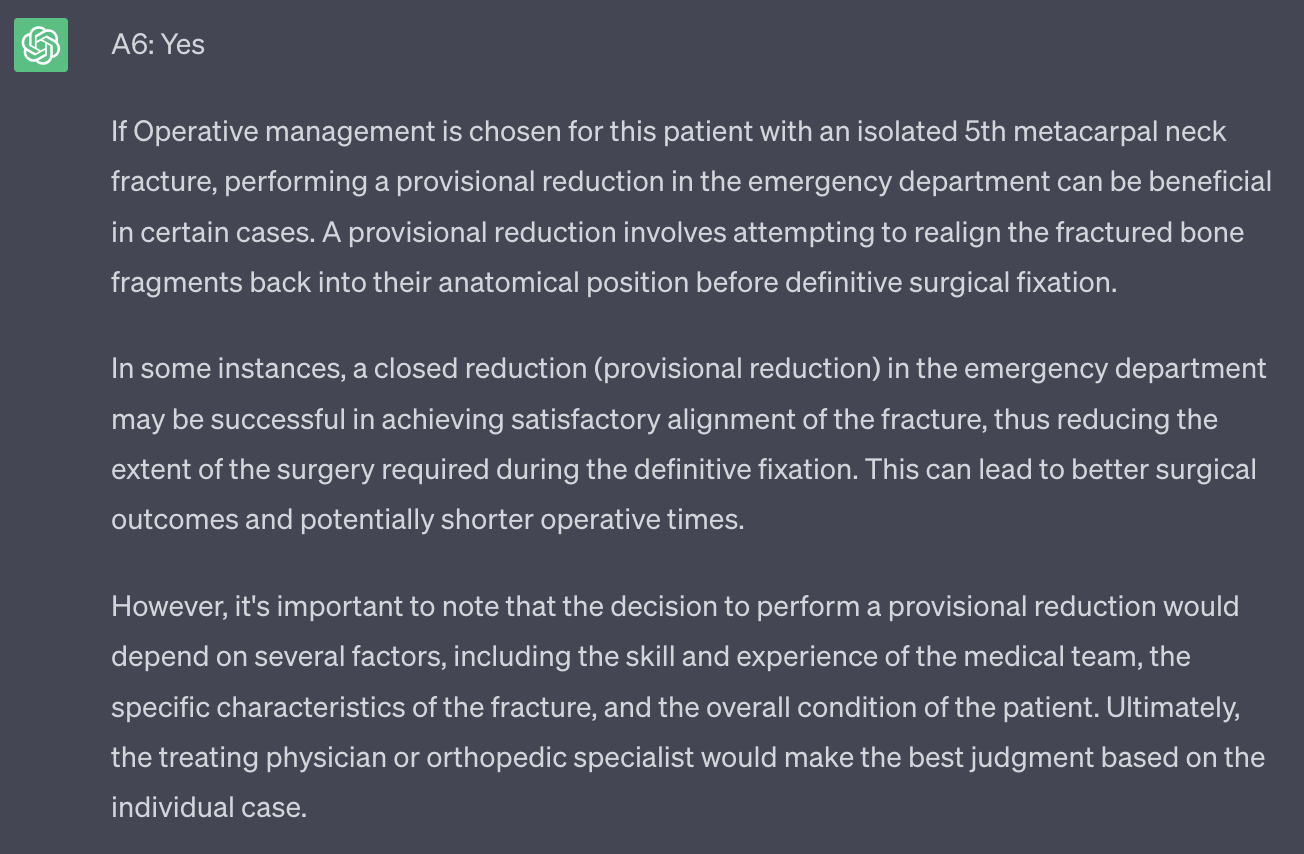


Q7: If you choose Operative management, what type of anesthesia would you use?

- I would not choose Operative management
- Wide awake (tumescent local anesthesia)
- Peripheral nerve block with sedation
- Peripheral nerve block without sedation
- General anesthesia
- Other


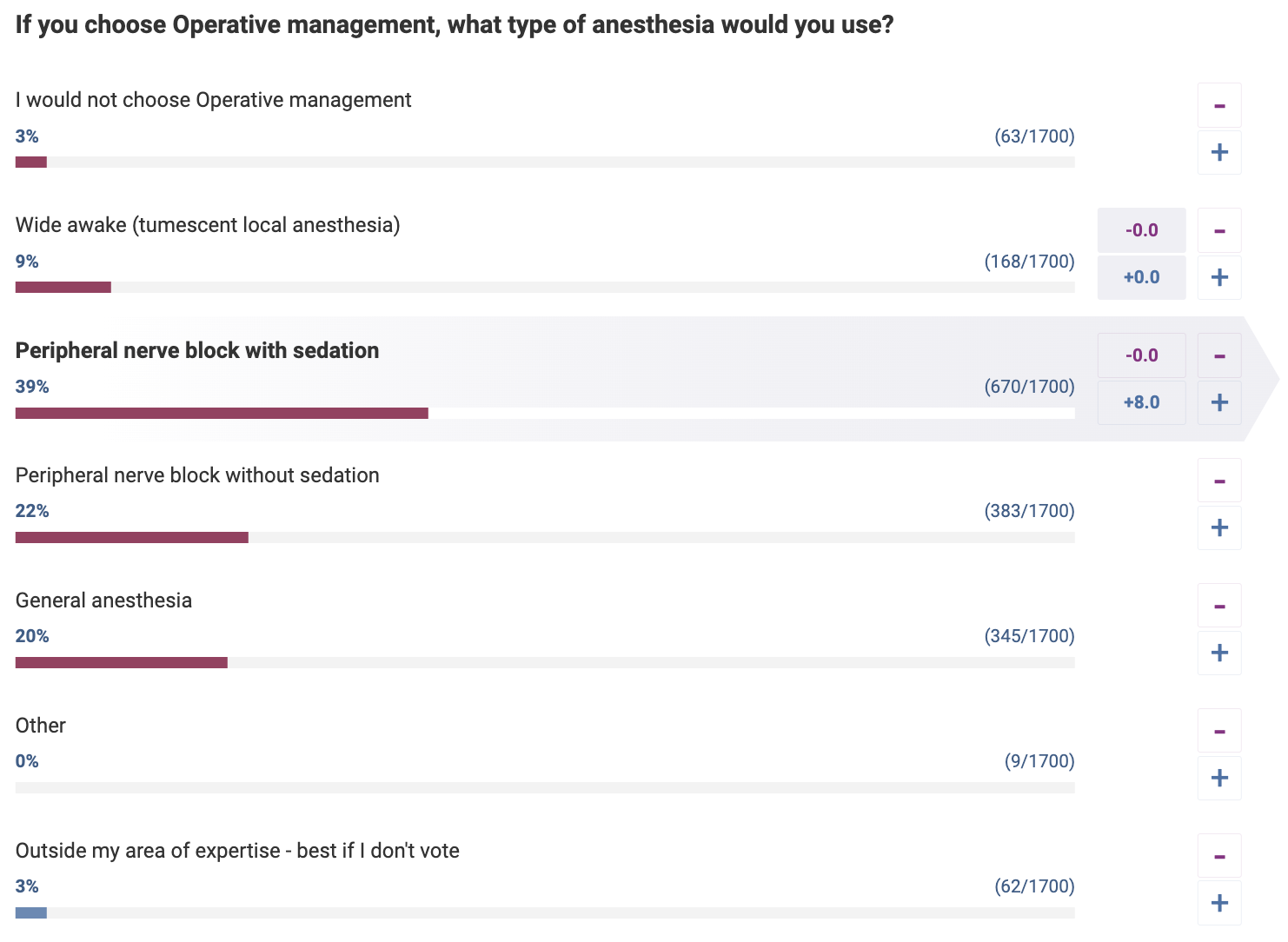


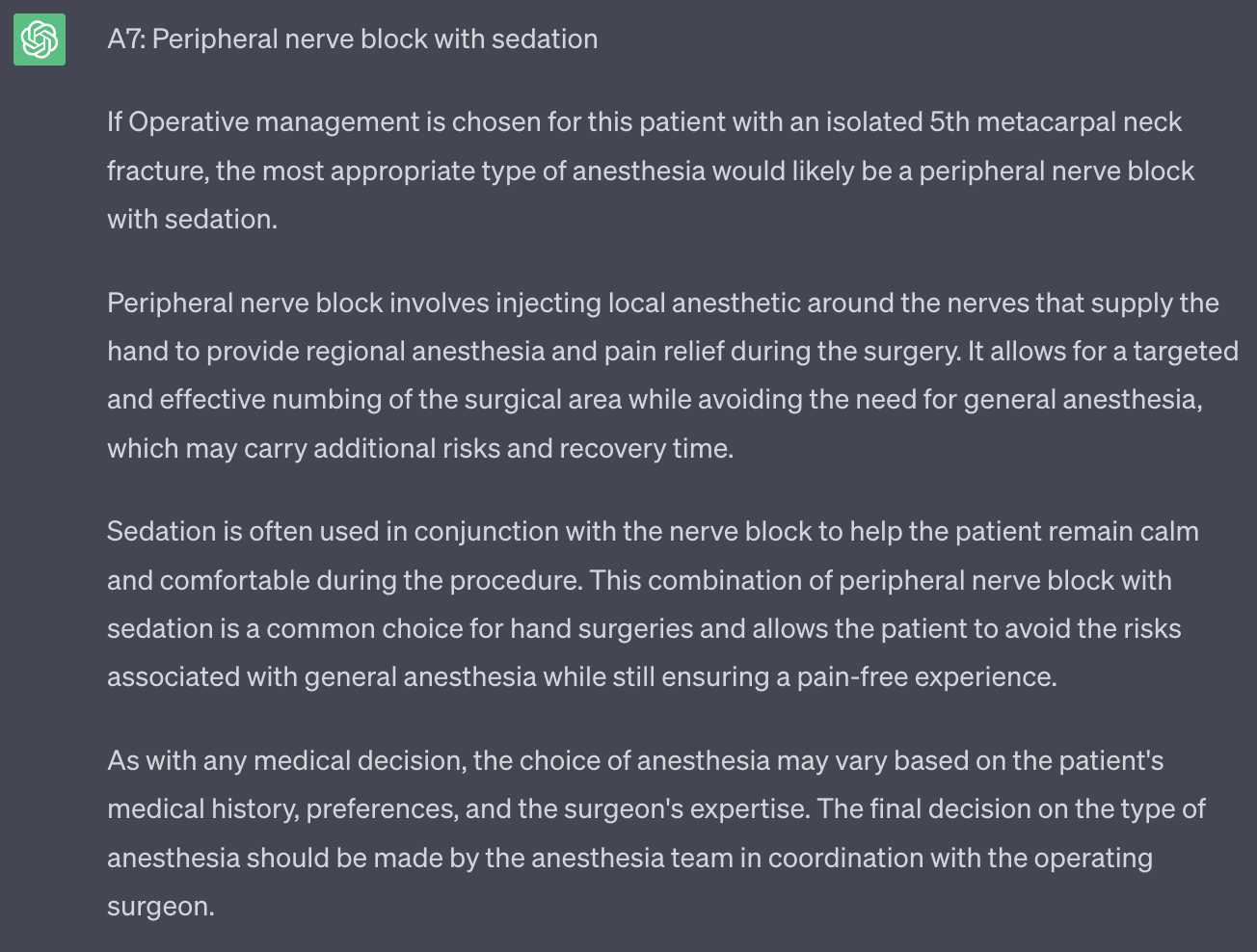


Q8: If you choose Operative management and attained the construct below (Well aligned reduction, single intra-medullary headless screw down the metacarpal), what type of immobilization would you apply postoperatively?

- I would not choose Operative management
- Soft dressings only
- Ulnar gutter splint
- Ulnar gutter cast
- Other


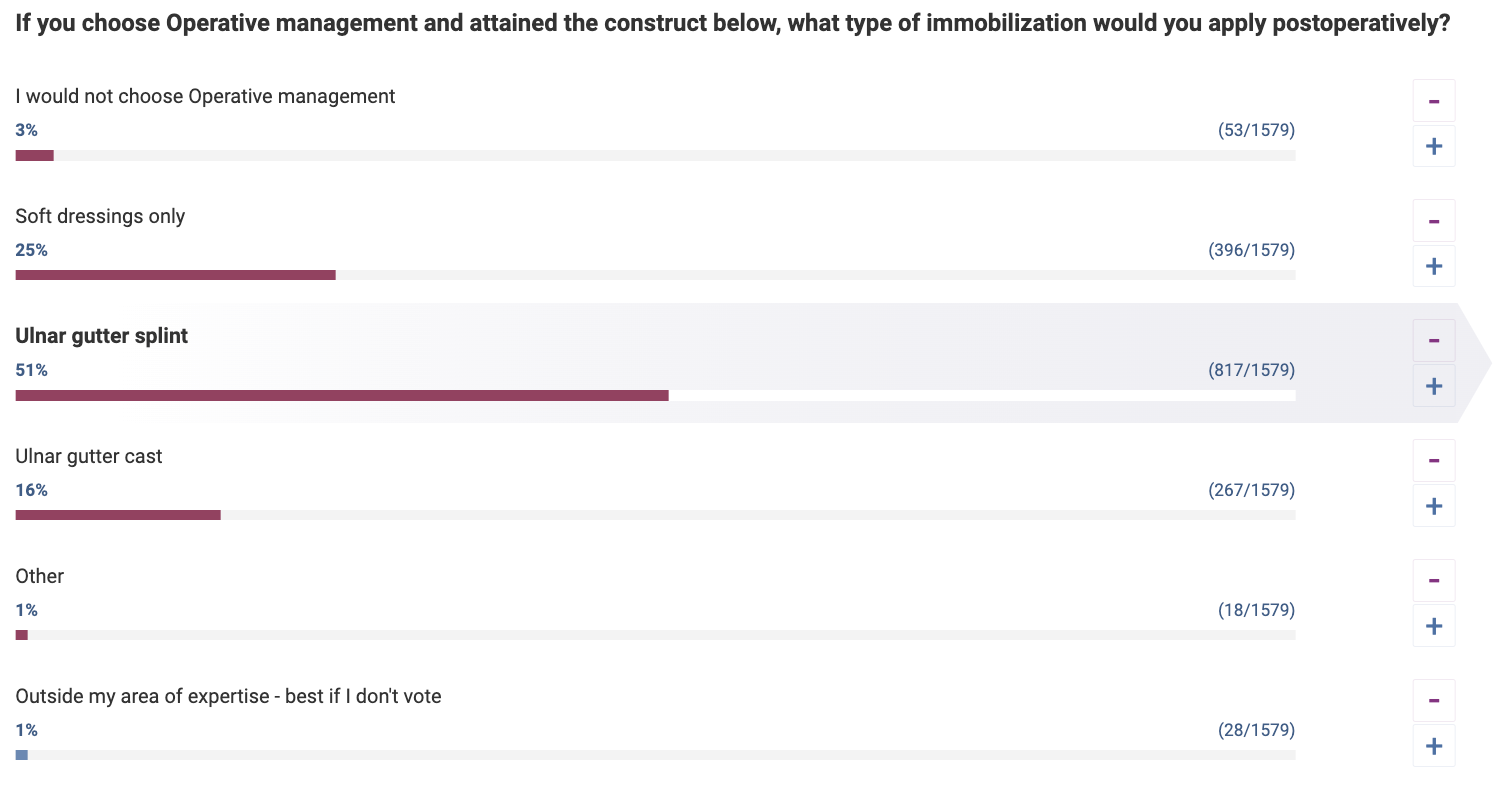


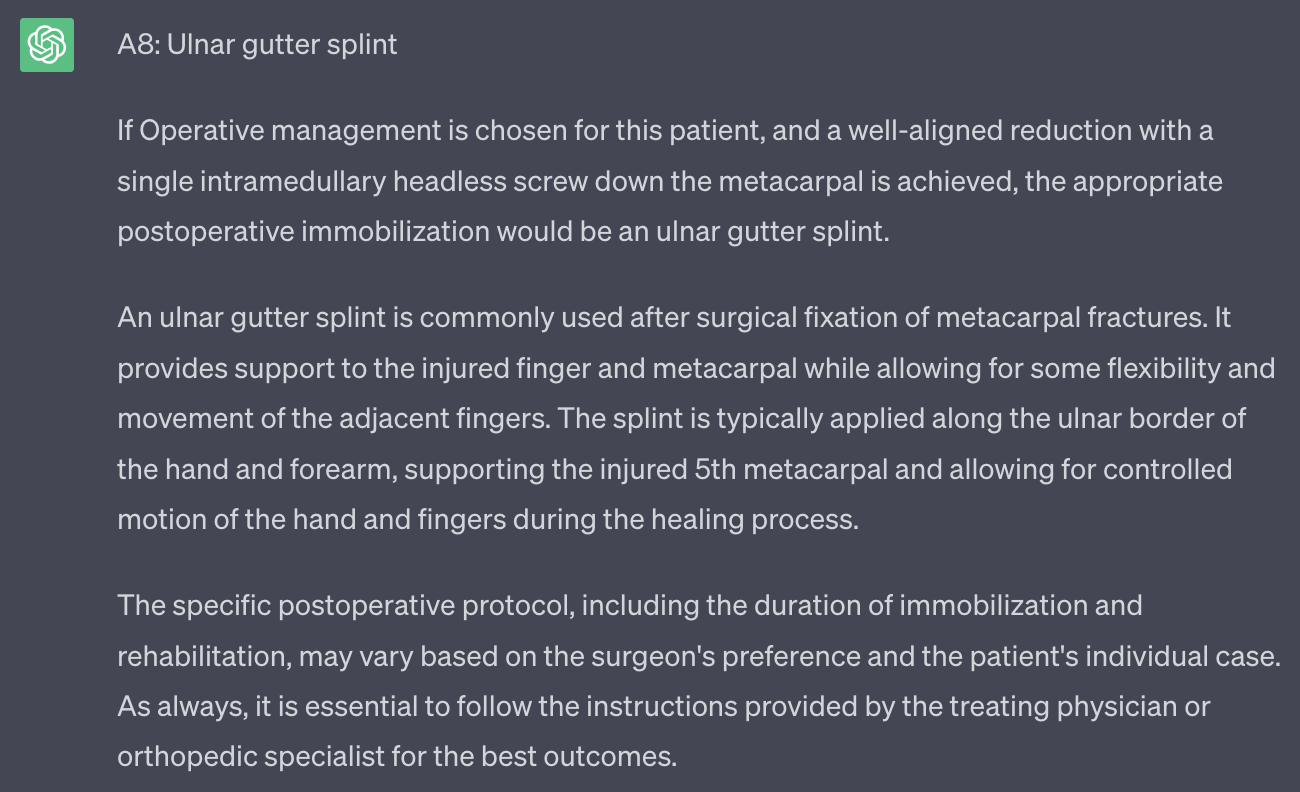


Q9: If you choose Operative management and attained the construct below (Well aligned reduction, single intra-medullary headless screw down the metacarpal), when would you begin range of motion post-operatively?

- I would not choose Operative management
- Immediately
- at 1-2 weeks
- at 3-4 weeks
- at 5-6 weeks
- at >6 weeks


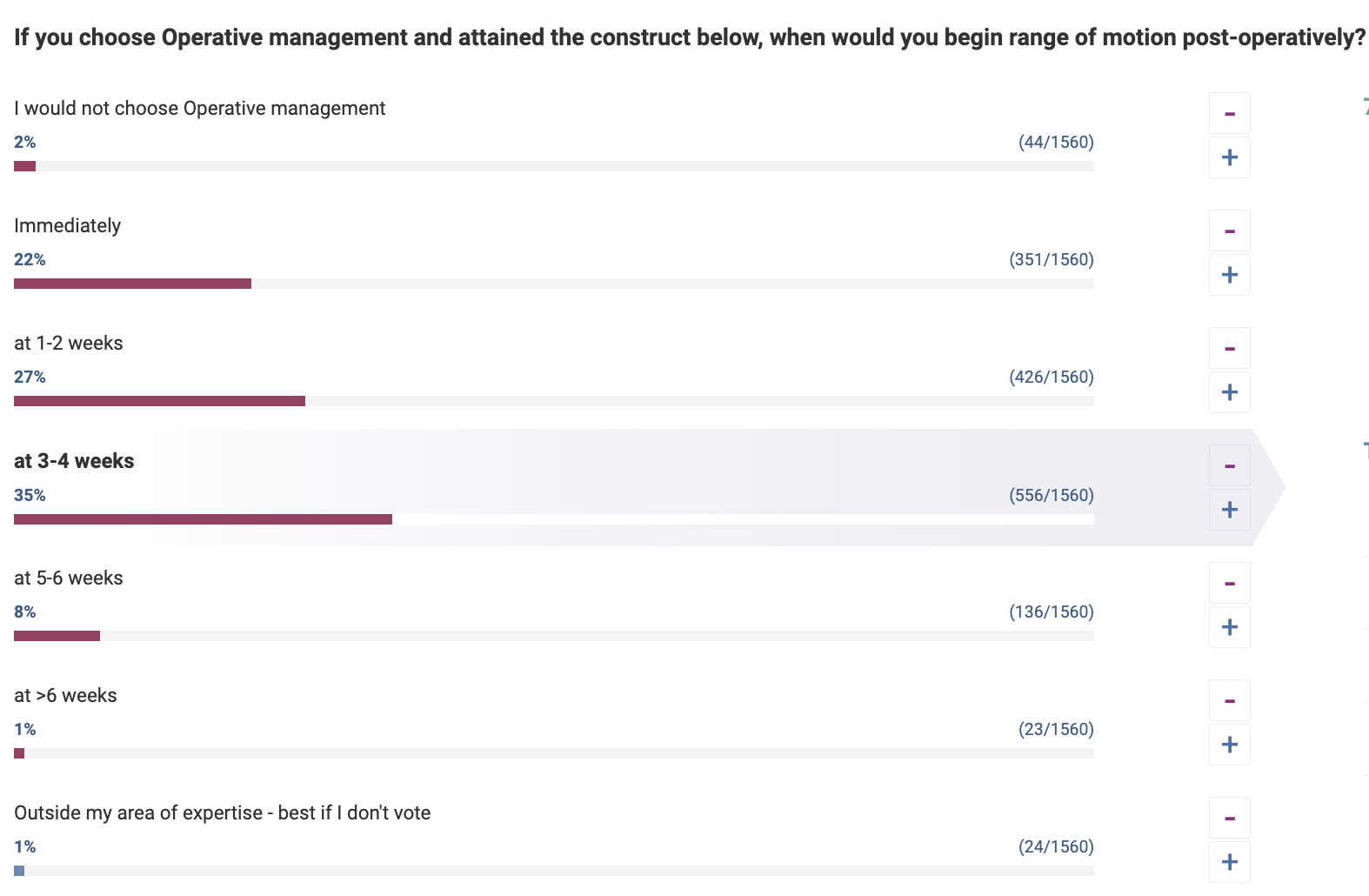


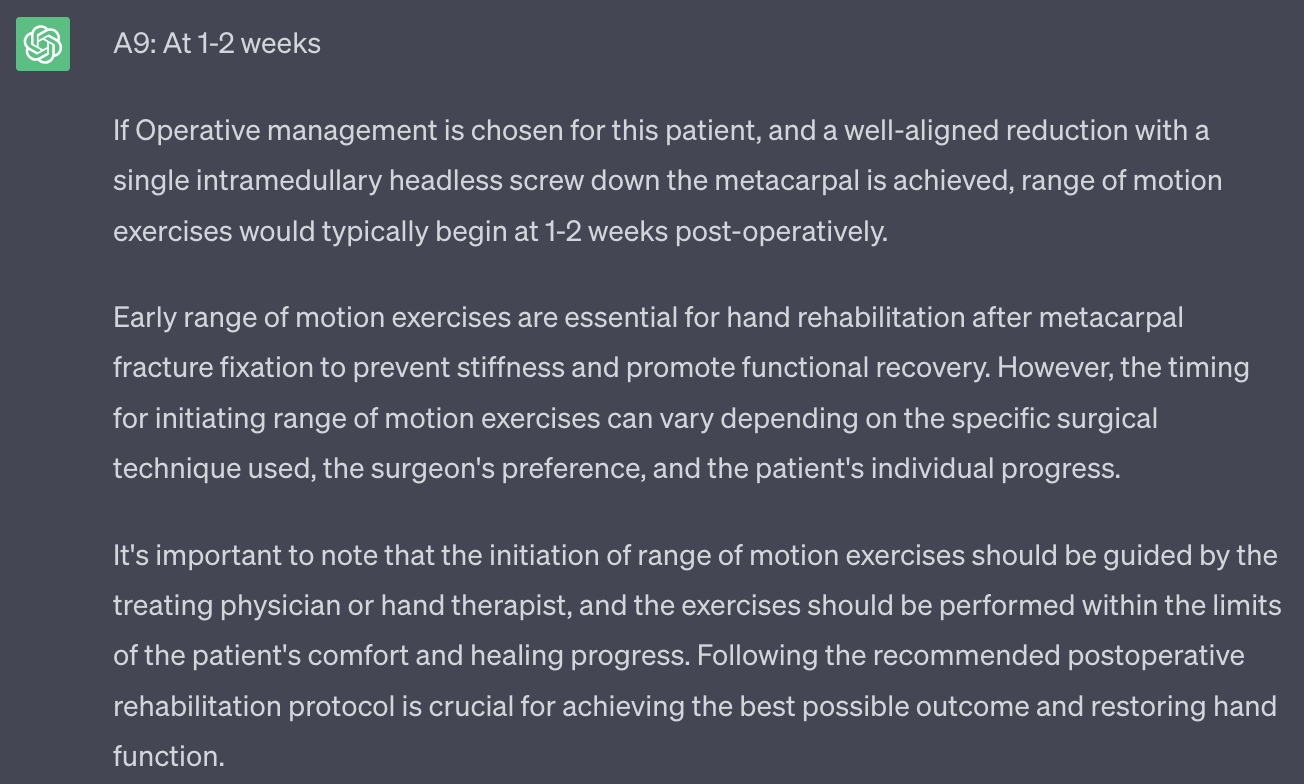


Q10: If you choose Operative management and attained the construct below (Well aligned reduction, single intra-medullary headless screw down the metacarpal), would you restrict weight-bearing post-operatively?

- I would not choose Operative management
- No - weight-bearing as tolerated (WBAT)
- Yes - for 1-2 weeks
- Yes - for 3-4 weeks
- Yes - for 5-6 weeks
- Yes - for 7-9 weeks
- Yes - for 10-12 weeks
- Yes - for > 12 weeks


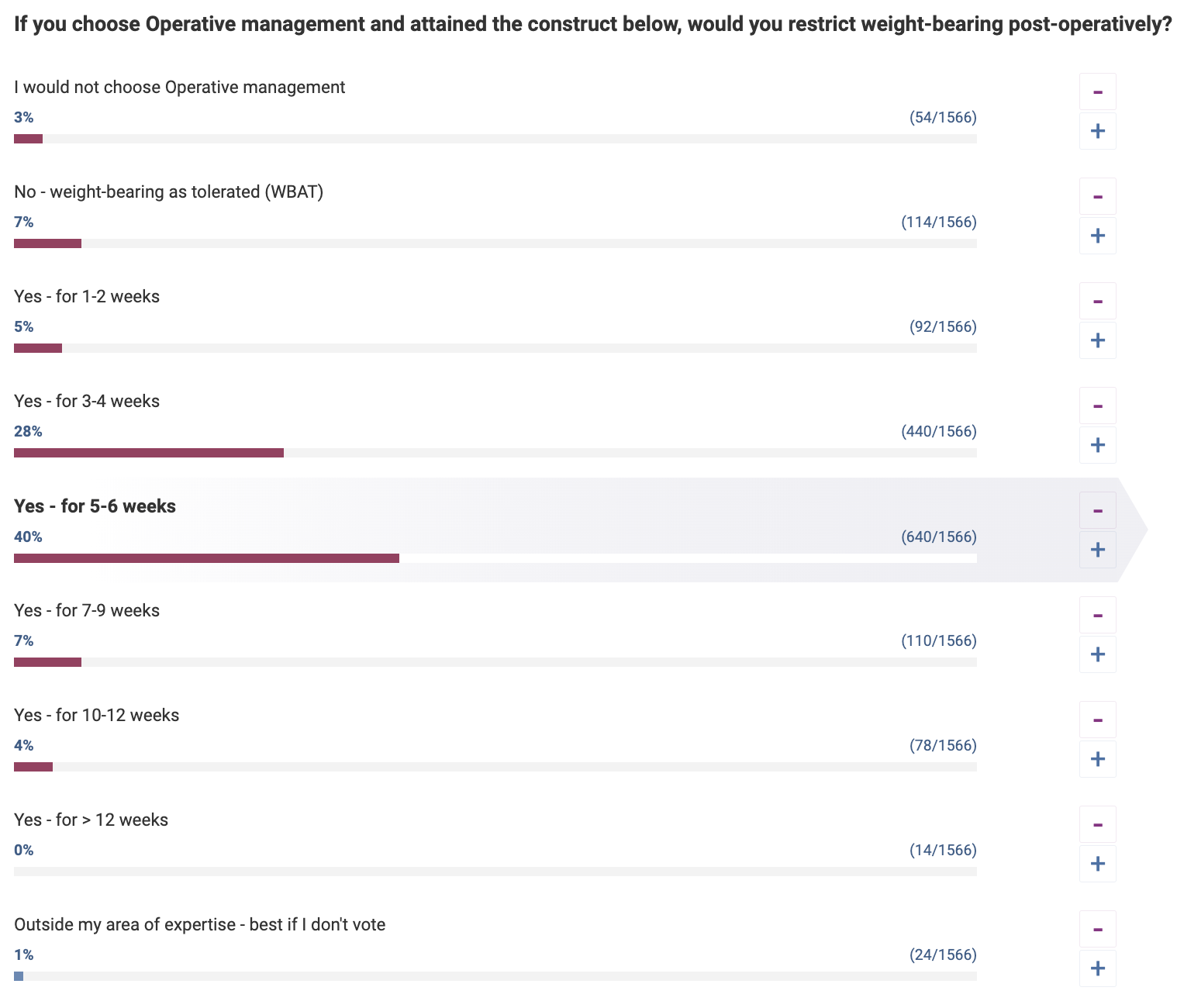


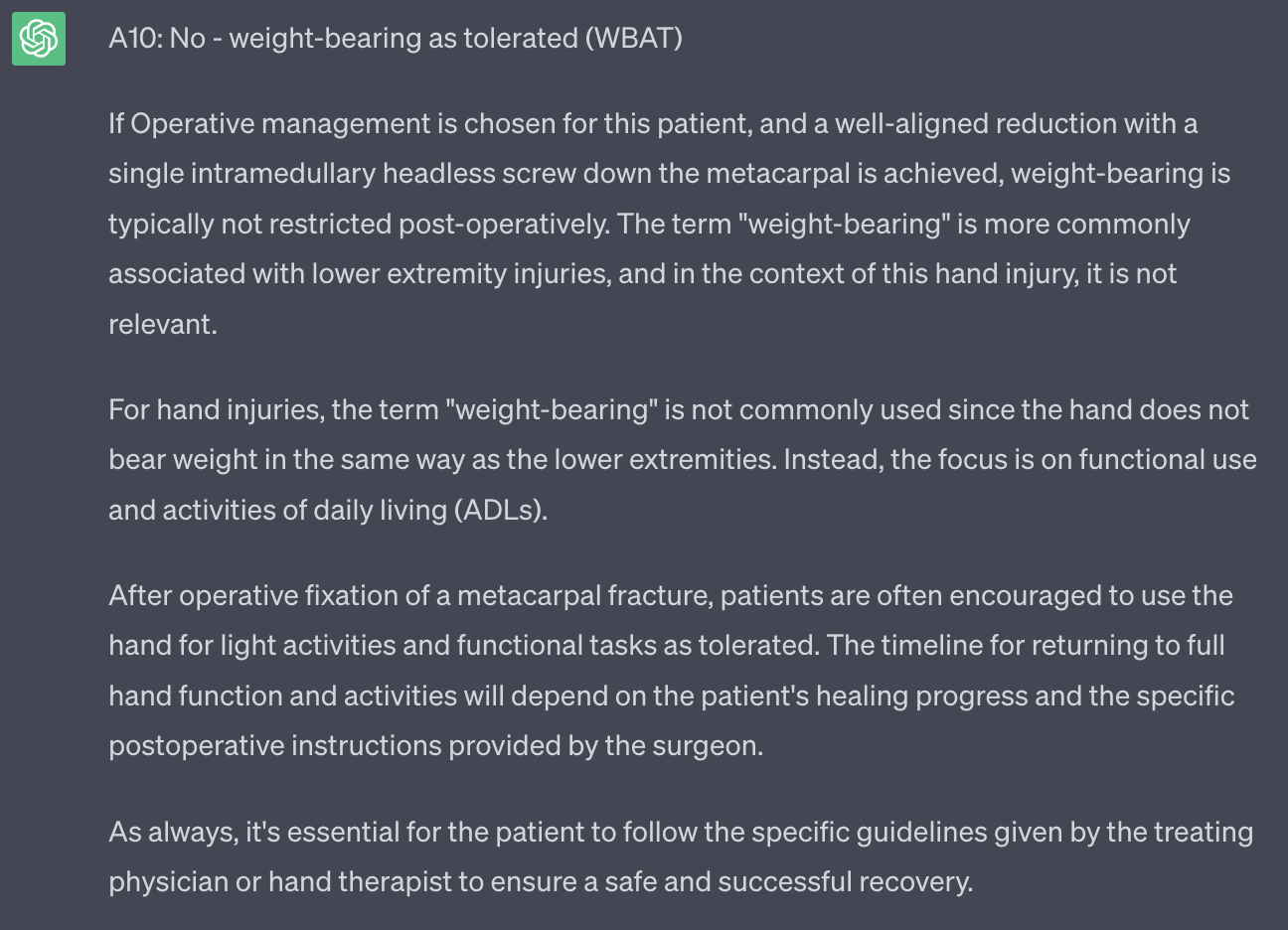


Title: Patellar tendon rupture in 31M

Category: Knee and Sports

Date: 20230724

Popularity: 1

Link: <https://www.orthobullets.com/Site/Cases/View/1968b4ac-2f07-4e6e-b8d2-ad6112e49c08>

Images:
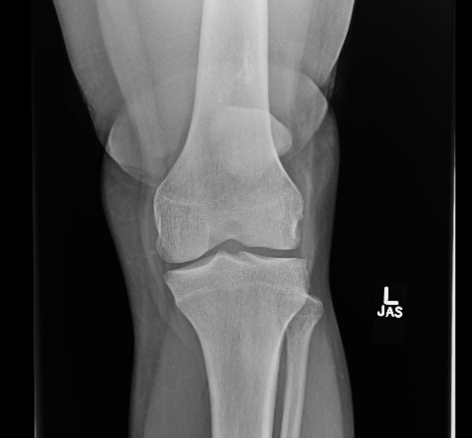

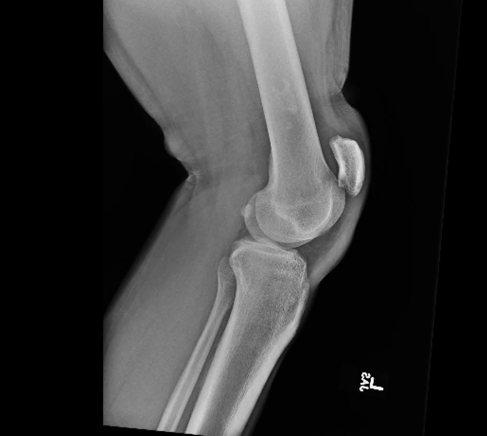


ChatGPT-3.5 dialogue:


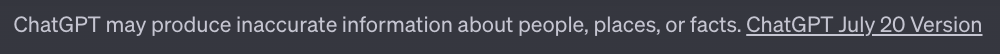


I am going to provide you a clinical vignette. There will be a series of 12 questions to follow. Please choose the single best responses for each question.

History of presenting incident:

A 31-year-old male presents to the ED for evaluation of a left knee injury. The patient reports he was playing basketball when he landed awkwardly after a lay-up. He fell to the ground and was unable to ambulate after the injury. He reports hearing an audible "pop". He denies any numbness or tingling

Past medical history:

None

Physical exam:
A focused exam demonstrates palpable knee effusion without any open wounds. A soft tissue defect is palpable at the inferior pole of the patella. The patient is unable to perform a straight leg raise. He is neurovascularly intact. His compartments are soft and compressible.

Imaging findings:

Plain AP and lateral x-ray radiographs of the L knee show patella alta with no fractures identified.

Q1: In addition to plain film radiographs, would you obtain any further imaging to guide your treatment?

- No - current radiographs are sufficient
- Yes - additional radiographs (XR)
- Yes - MRI of the knee (MRI)
- Yes - CT of the knee (CT)
- Yes - XR + MRI
- Yes - XR + CT
- Yes - CT + MRI
- Yes - XR + CT + MRI


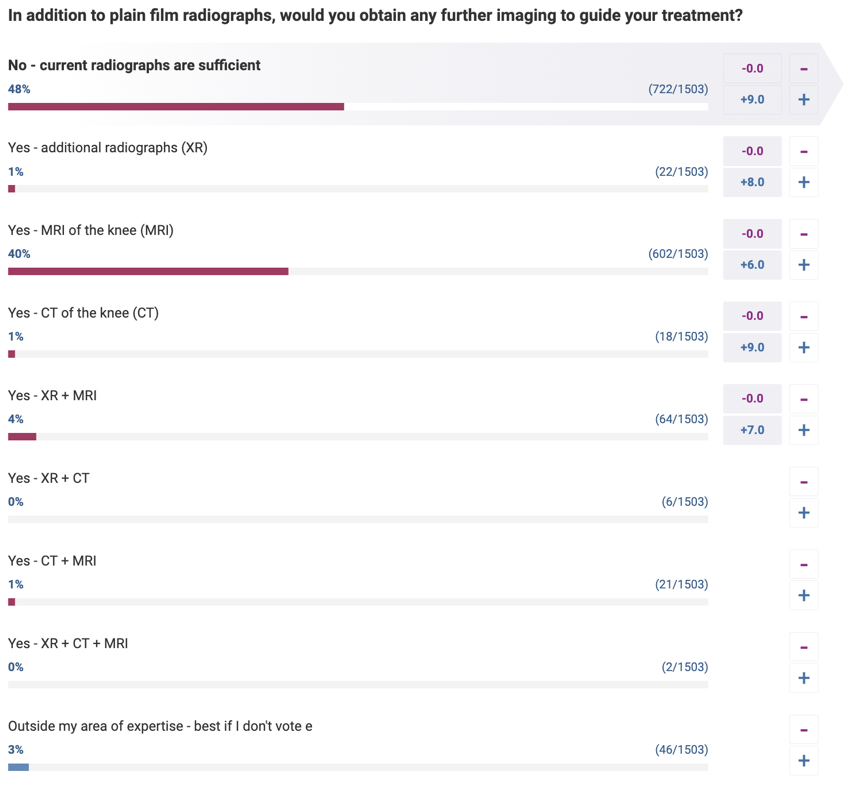


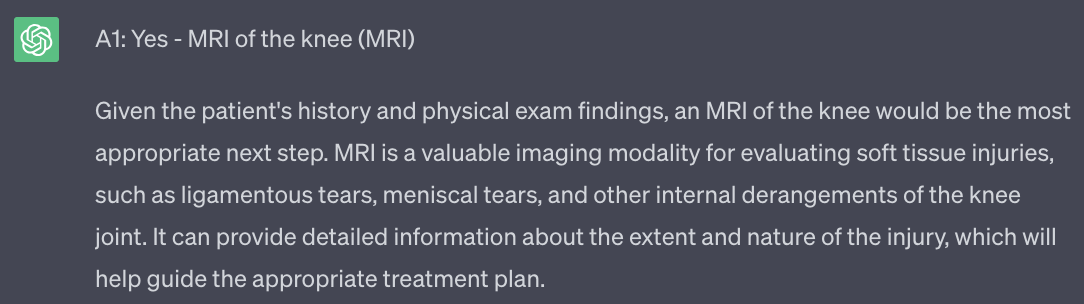


Q2: Would you use a radiographic measurement to help guide the treatment of this injury?

- No - a radiographic measurement would not help me
- Yes - Insall-Salvati ratio
- Yes - Caton-Deschamps index
- Yes - Blackburne-Peel ratio
- Yes – Other


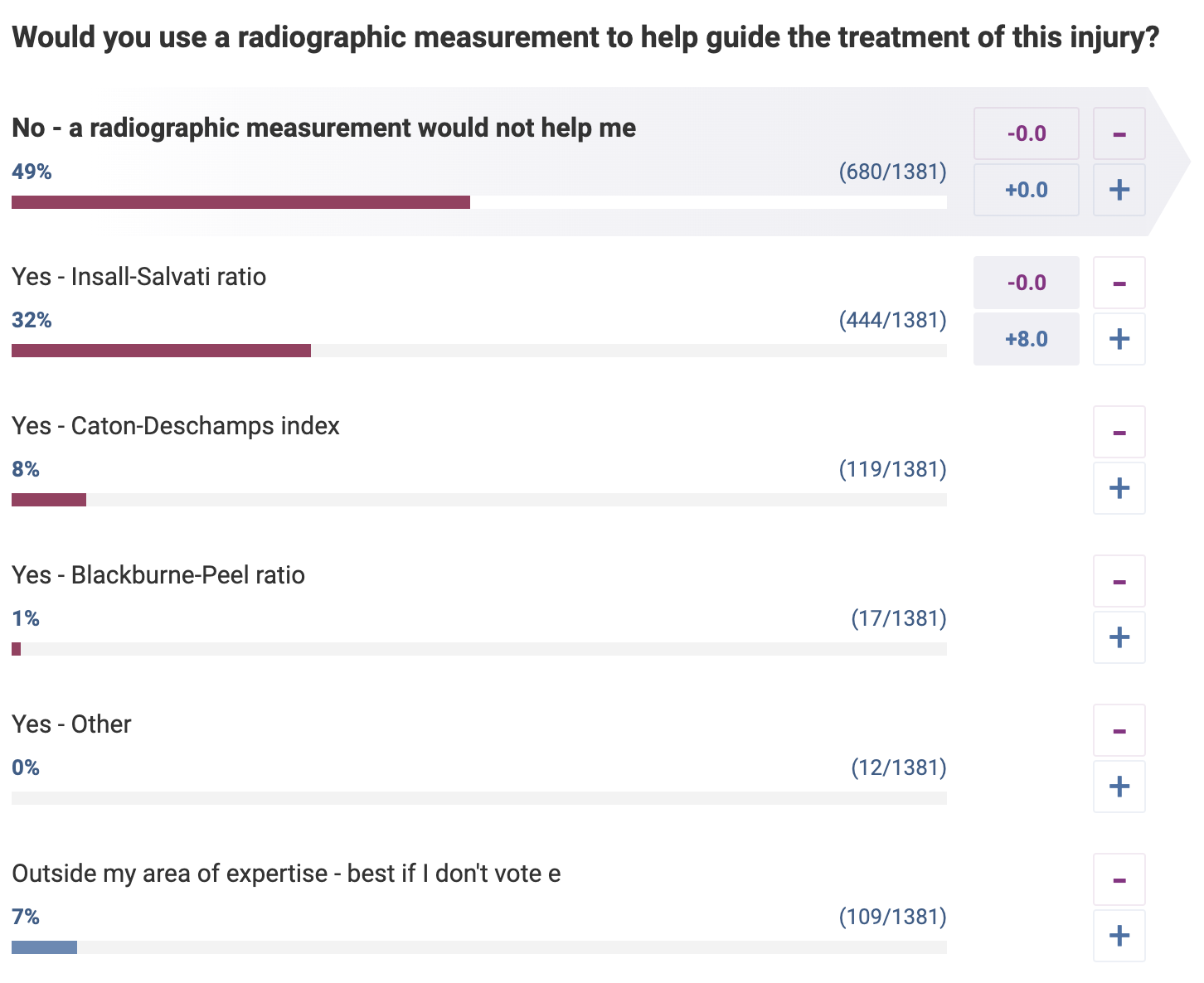


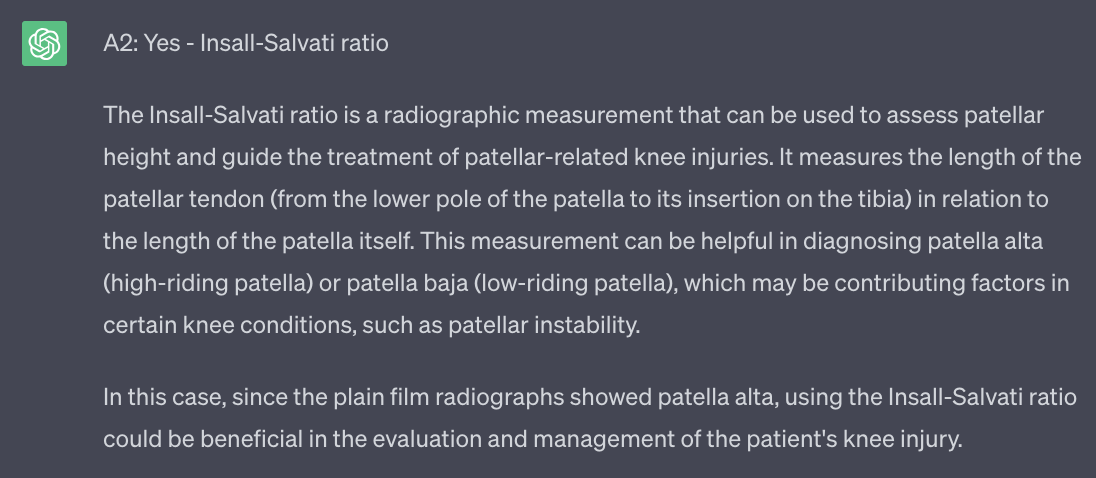


Q3: How would you manage this injury?

- Nonoperative
- Operative


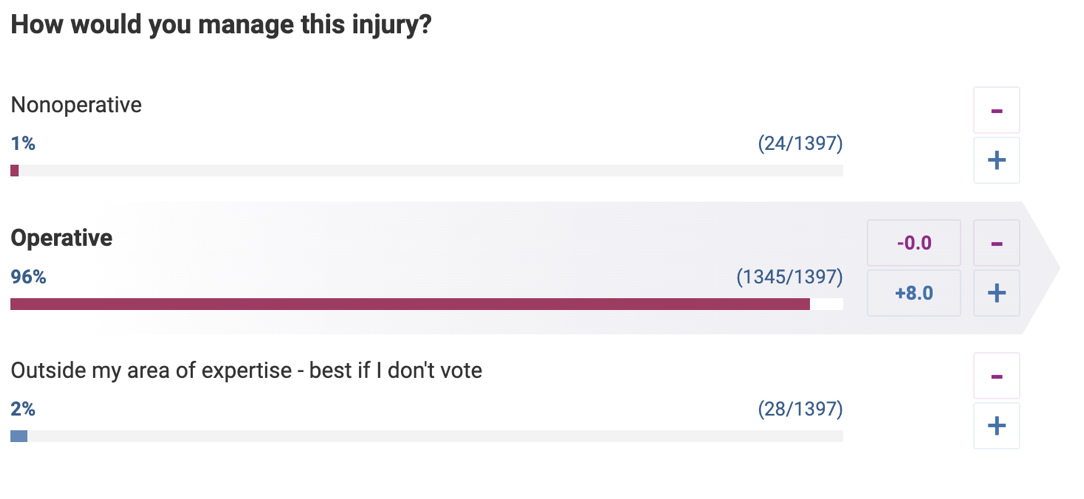


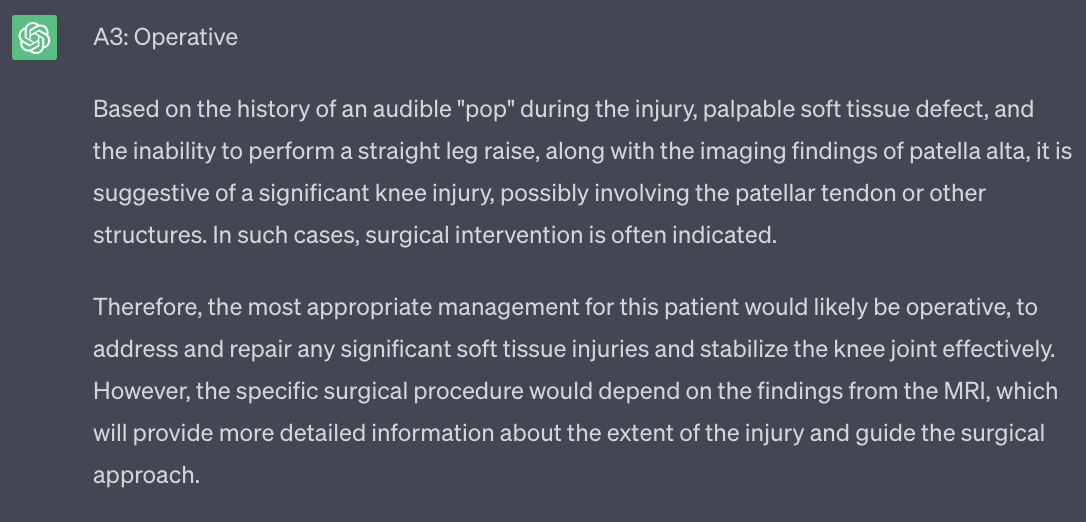


Q4: If you choose Operative management, what procedure would you plan to utilize?

- I would not choose Operative management
- Direct primary repair (includes suture anchors, transosseous tunnels etc)
- Reconstruction (includes allograft, autograft etc)


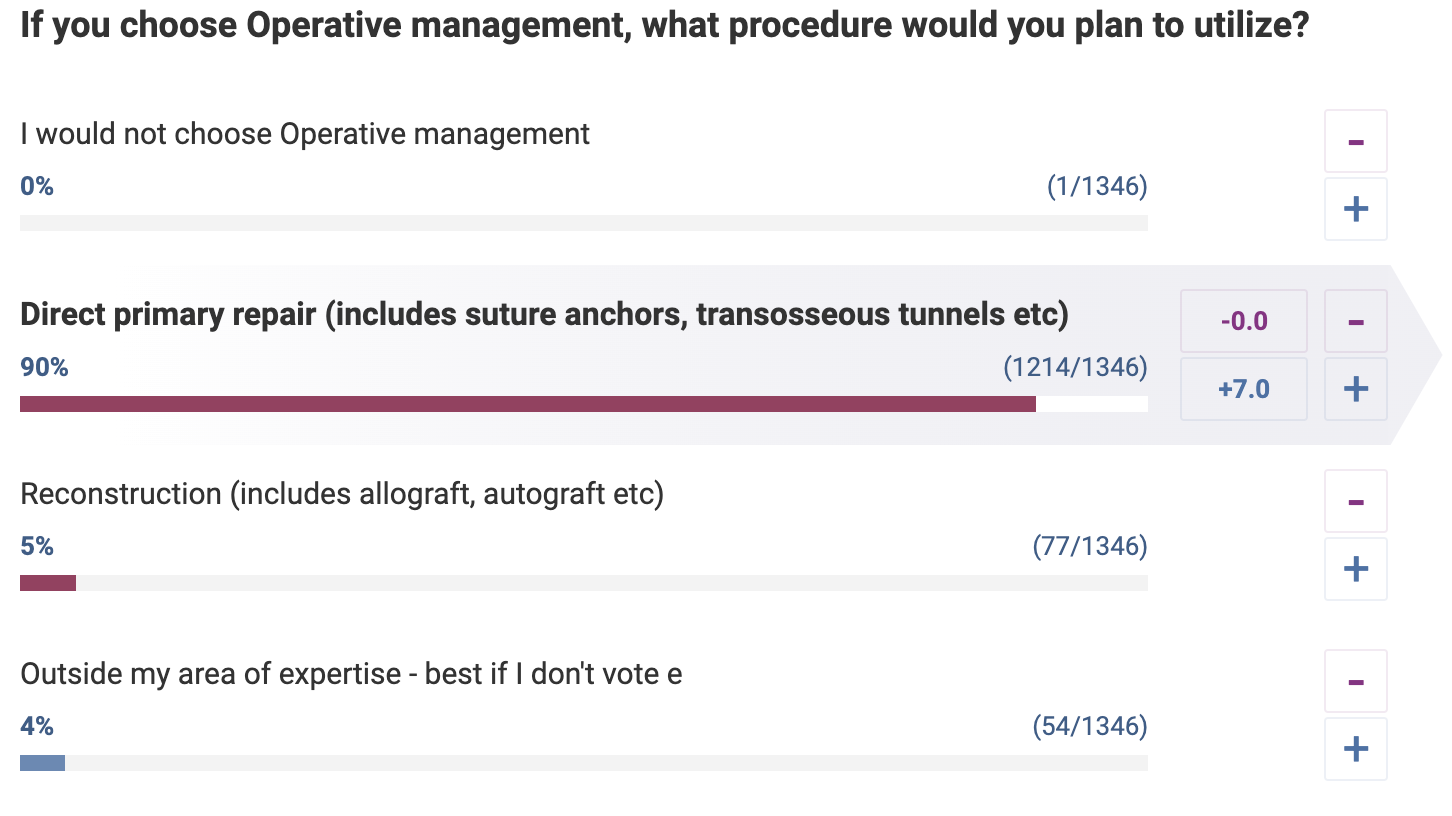


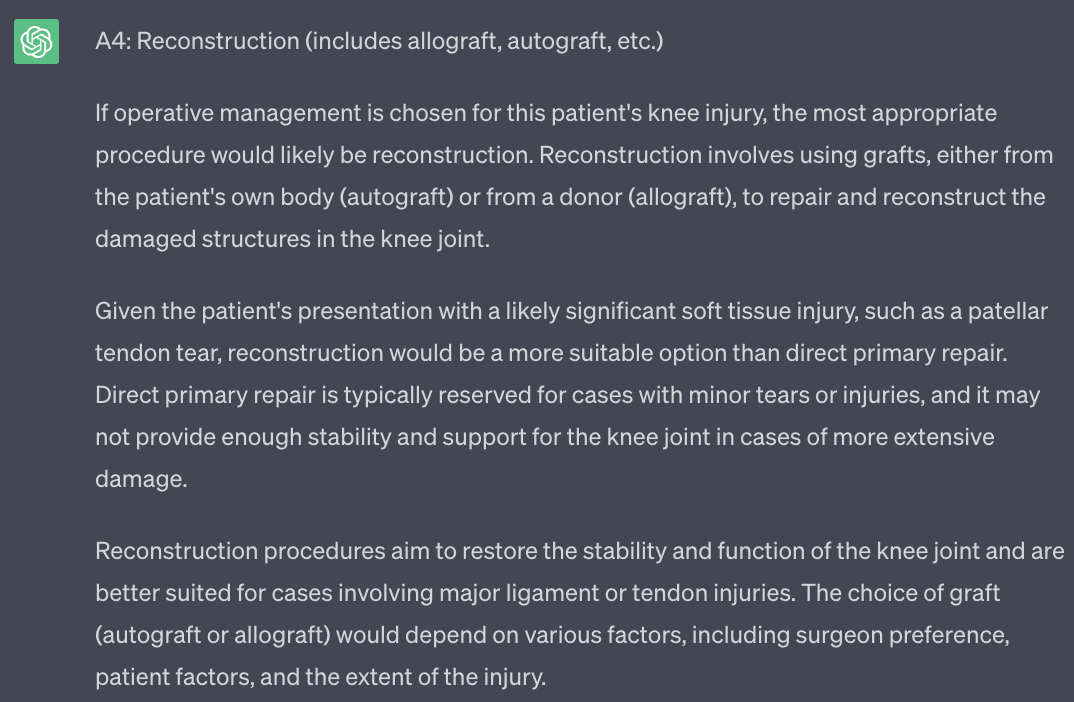


Q5: If you choose Direct primary repair, what would be your deadline/limit to perform the surgery considering he presented to the ER the same day of injury?

- I would not choose Operative management
- Same Day
- Next Day (day after presentation)
- Within 3 Days
- Within 7 Days
- Within 14 Days
- Within 30 Days


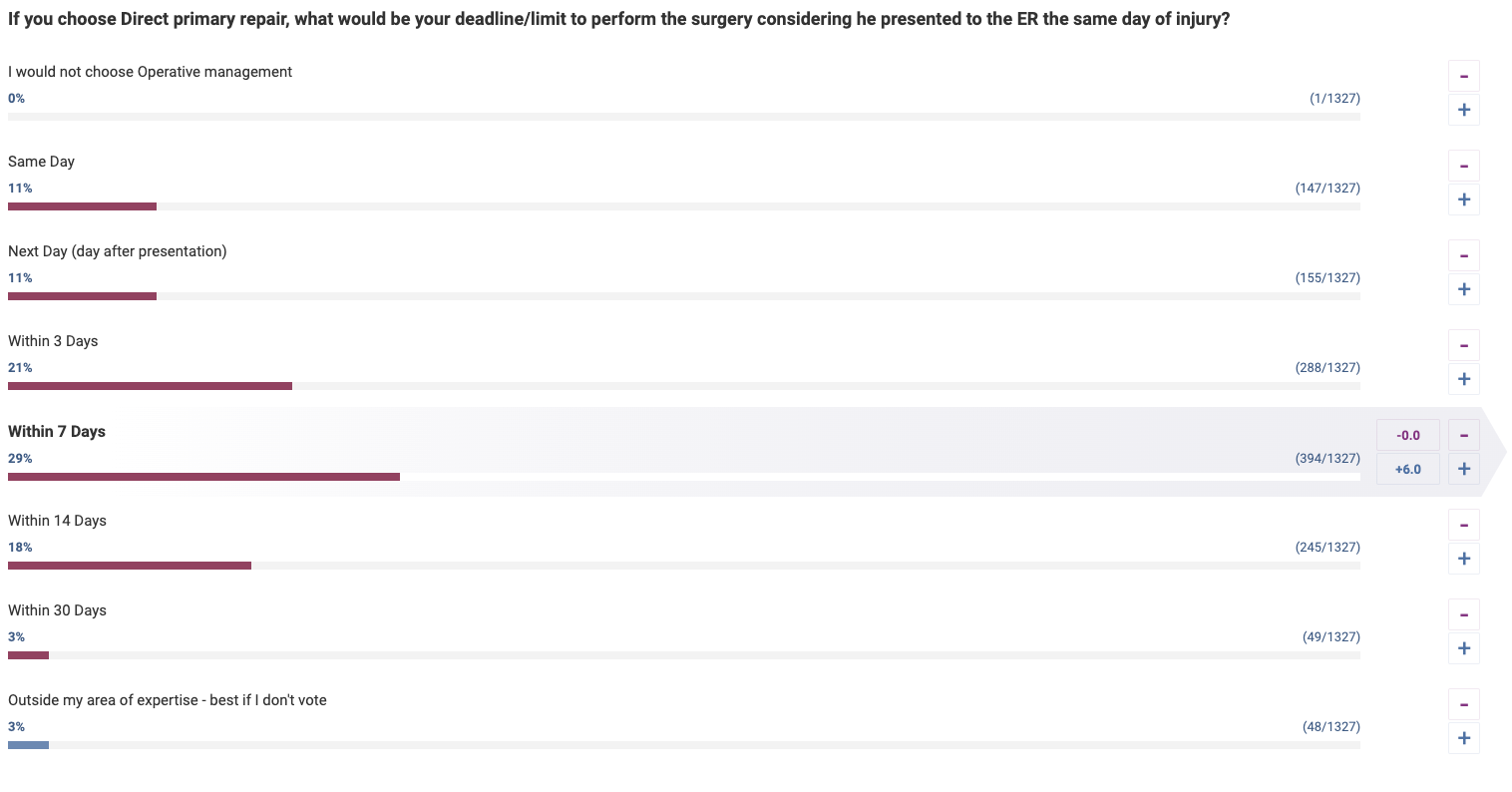


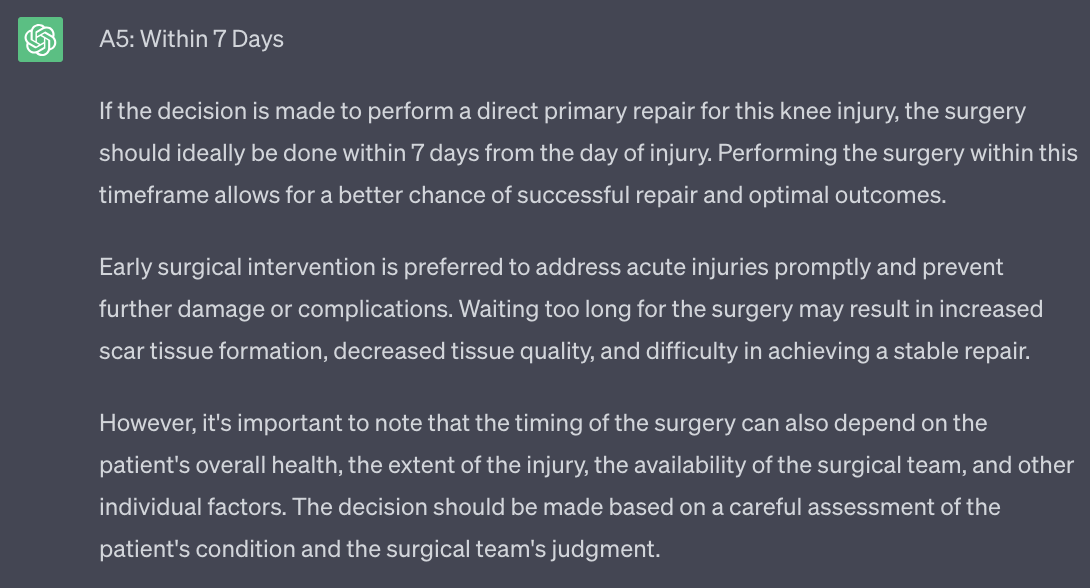


Q6: If you chose Direct Primary Repair, would you utilize or augment your anesthesia with a peripheral nerve block?

- I would not choose Direct Primary Repair
- No - I would not use a peripheral nerve block
- Yes - Femoral Nerve Block
- Yes - Adductor Canal Block
- Yes - Lumbar plexus/ psoas compartment Block


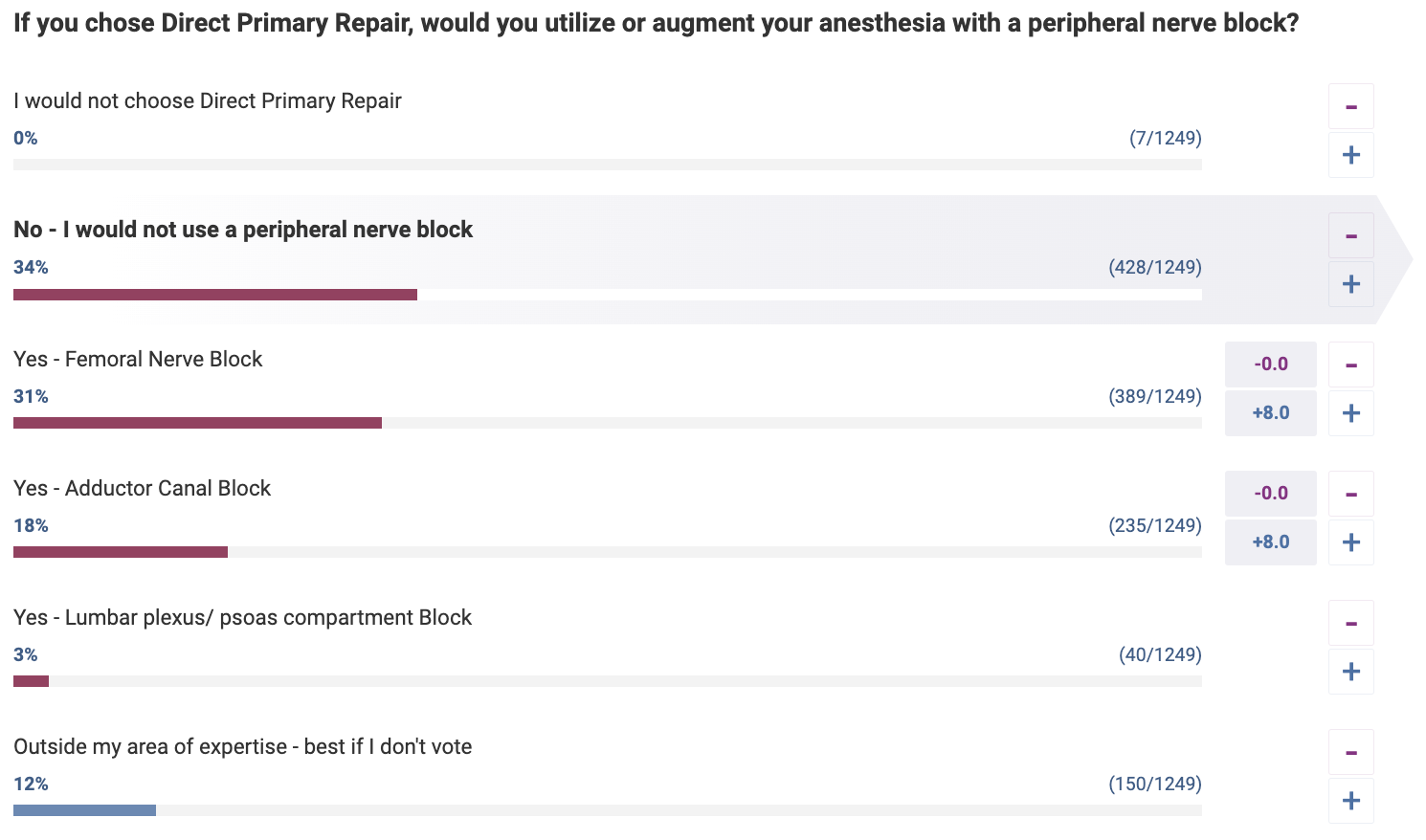


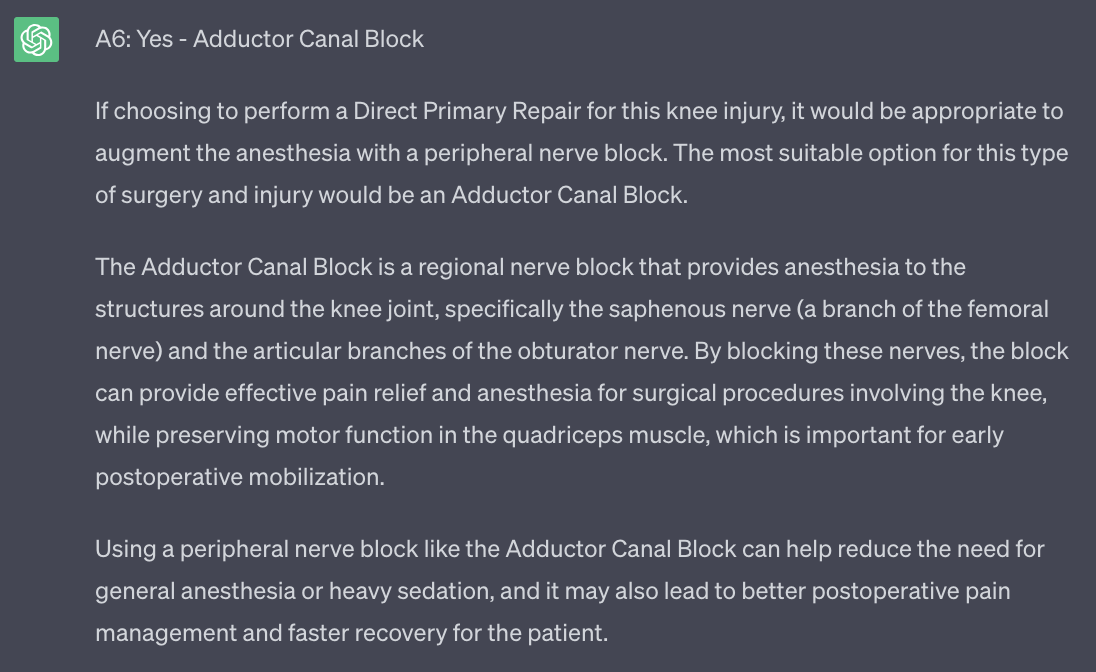


Q7: If you choose Direct Primary Repair, which technique would you utilize?

- I would not choose Direct repair
- End-to-end repair only (EER)
- Transosseous tunnels only (BT)
- Suture anchor(s) only (SA)
- Combined EER + BT
- Outside my area of expertise - best if I don't vote
- Combined EER + SA


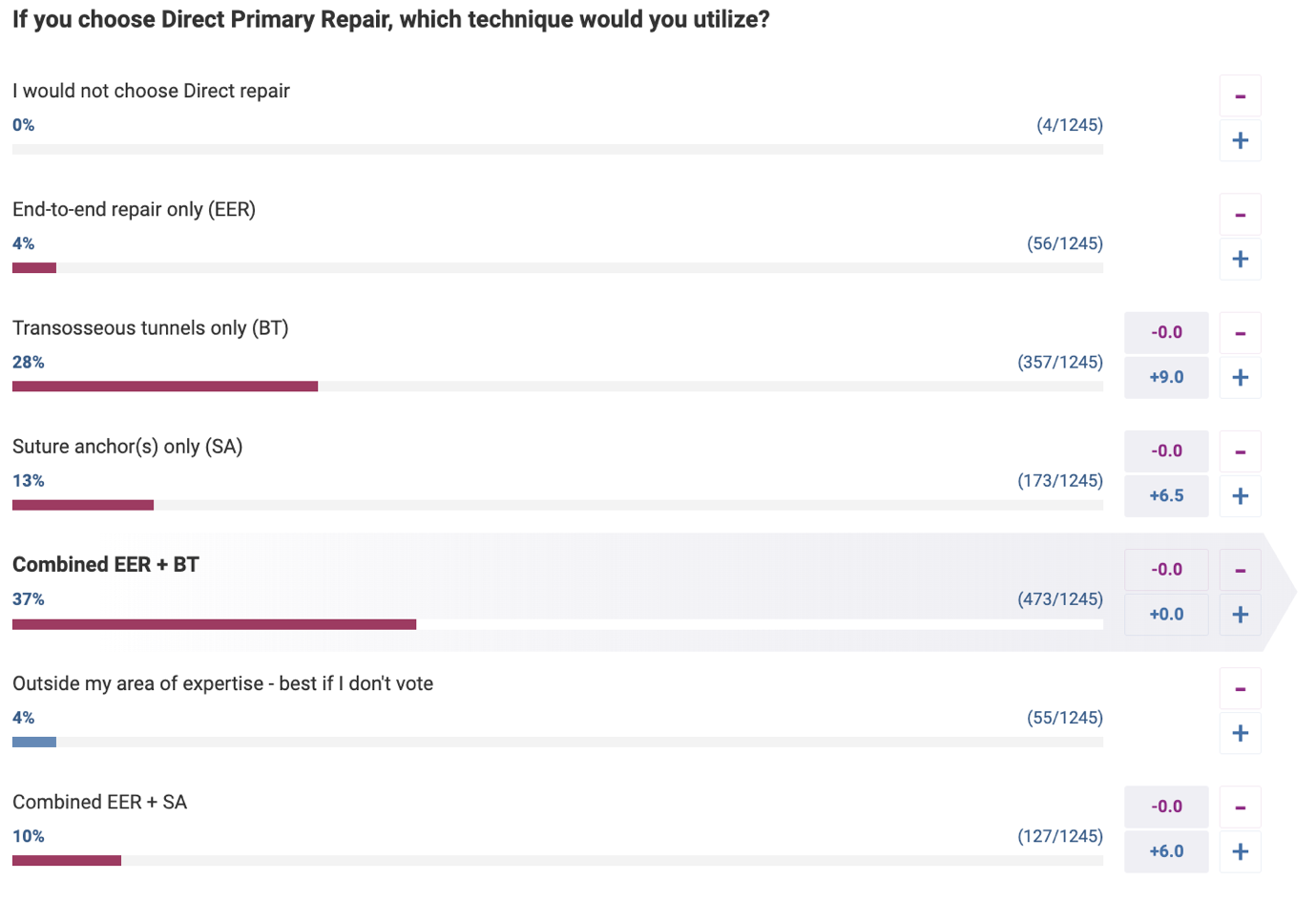


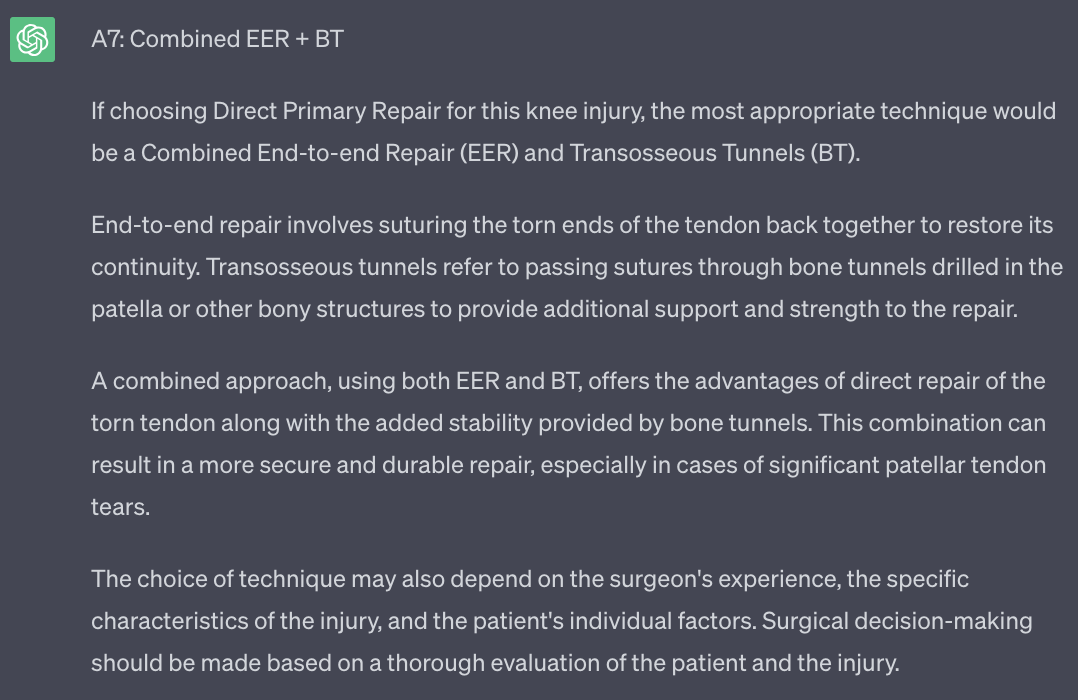


Q8: If you choose Direct Repair with Suture anchor(s) how many anchors would you plan on using?

- I would not choose Direct Repair with Suture anchor(s)
- 1
- 2
- 3
- 4
- 5+


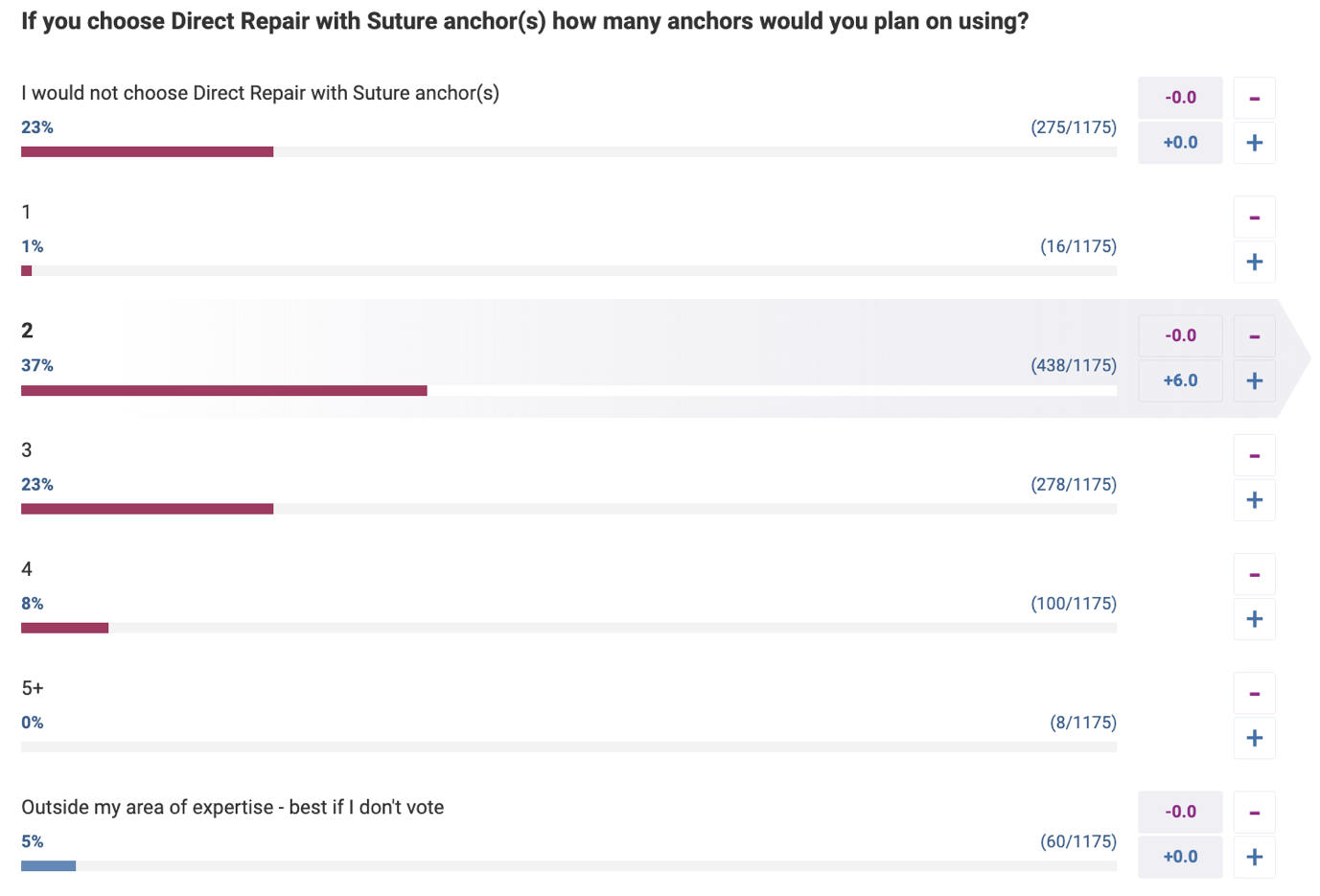


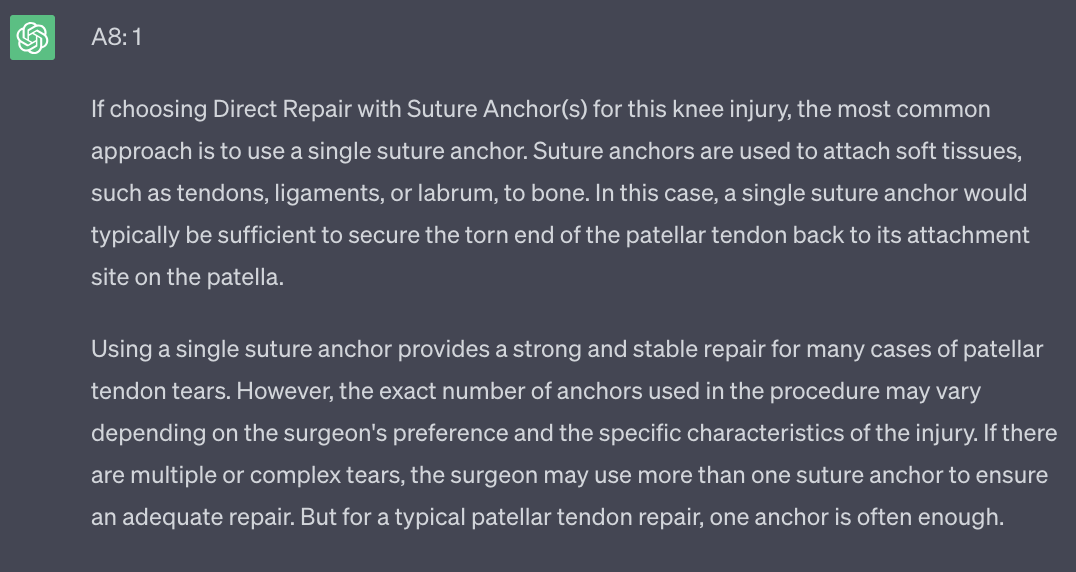


Q9: If you choose Direct Repair with Suture anchor(s), would you augment your repair?

- I would not choose Suture anchor repair
- No - I would not augment my repair
- Yes - I would augment with Allograft (e.,g semi-T)
- Yes - I would augment with Autograft (e.g., Hamstring)
- Yes - I would augment with Cerclage Wire
- Yes - I would augment with Synthetic Suture (e.g., Fiber Wire)


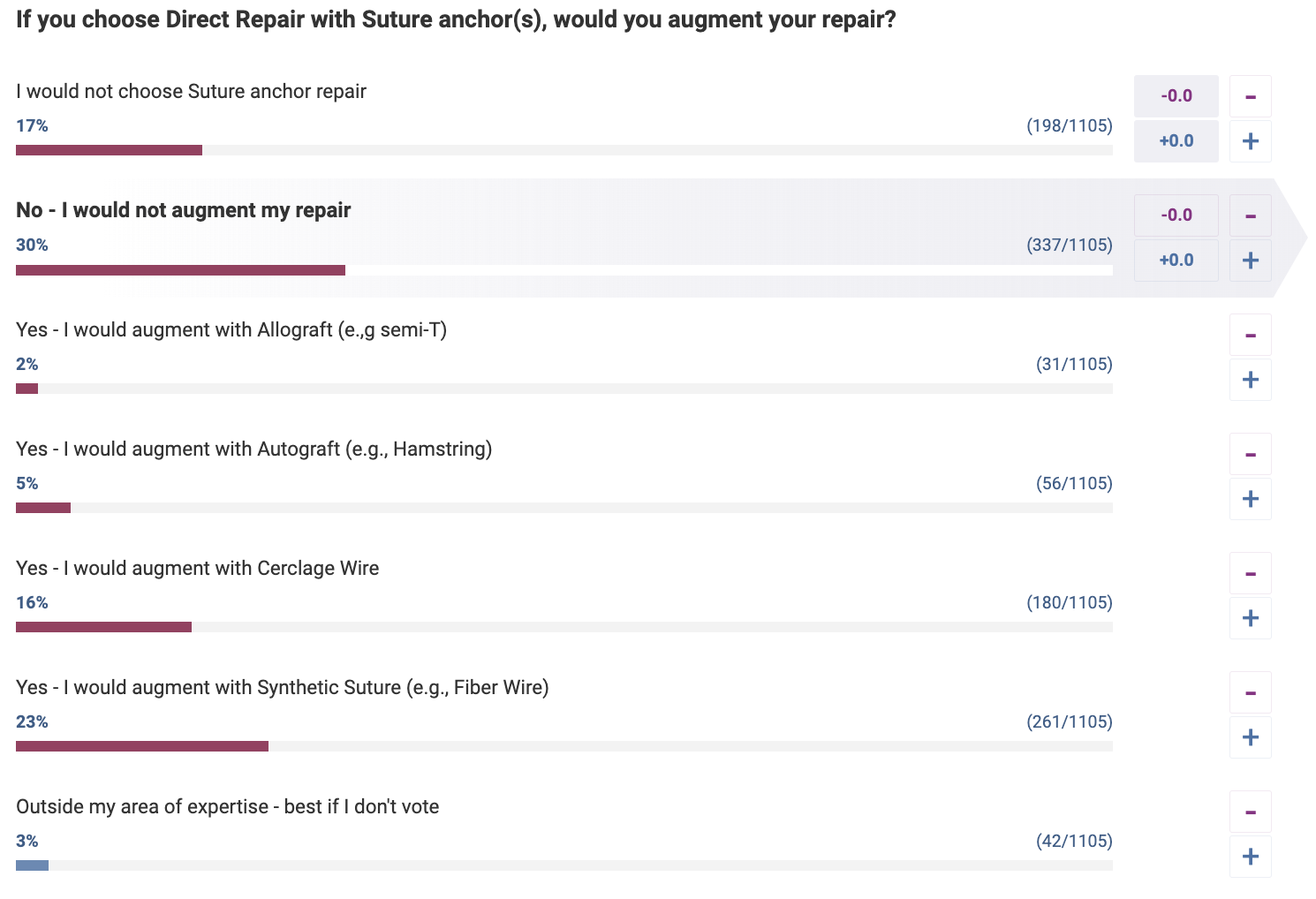


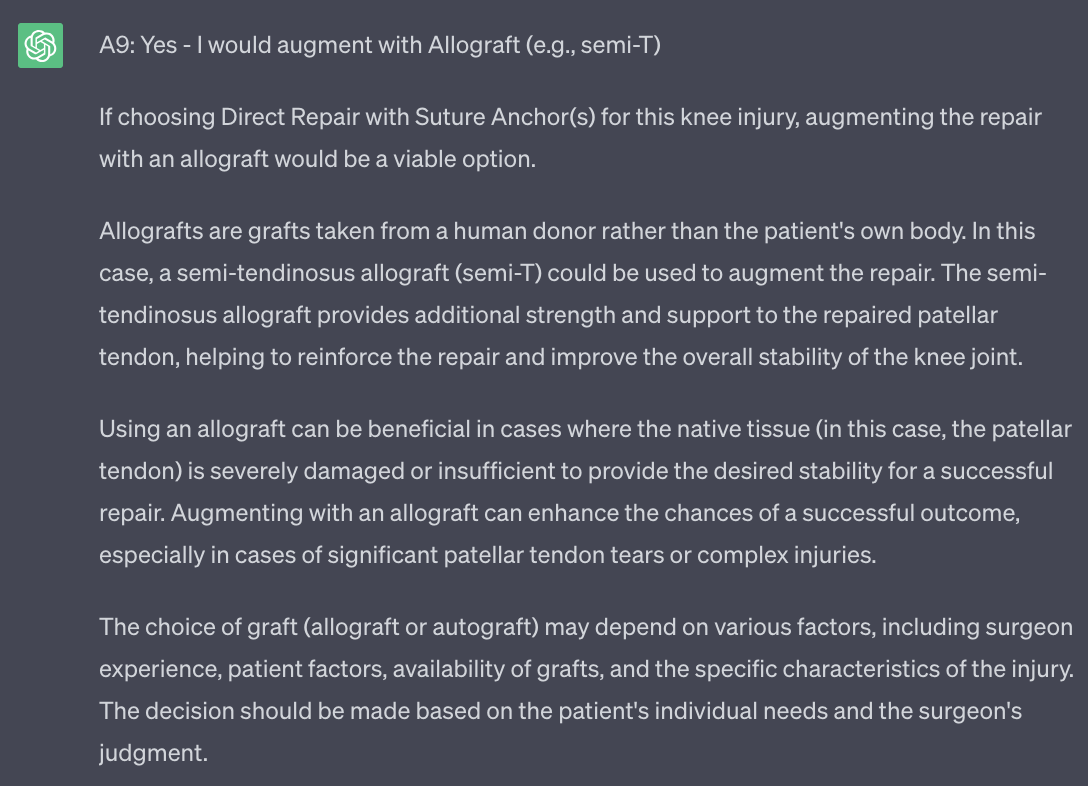


Q10: If you choose Direct Repair with Suture anchor(s) and obtained the construct below, when would you allow the patient to begin Active Flexion?

- I would not choose Suture anchor repair
- < 2 weeks
- 2-4 weeks
- 5-6 weeks
- 7-8 weeks
- 9-12 weeks
- >12weeks


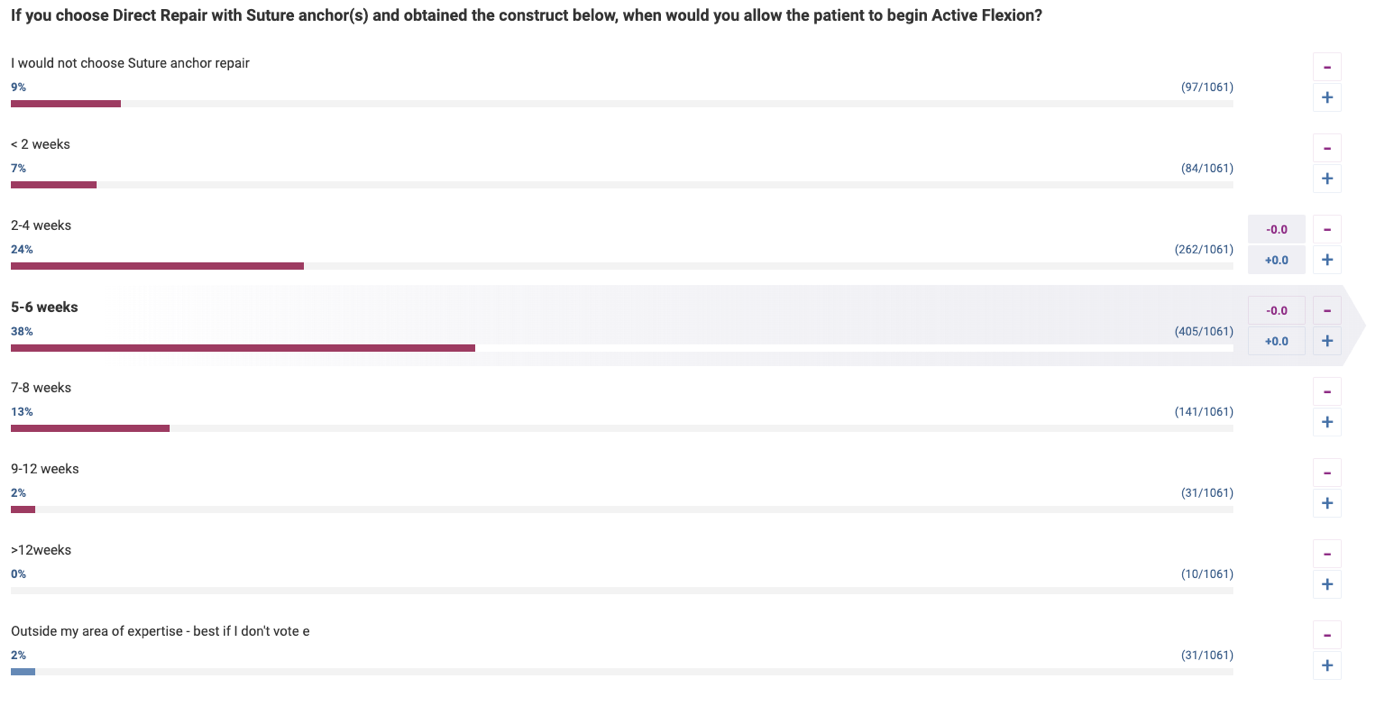


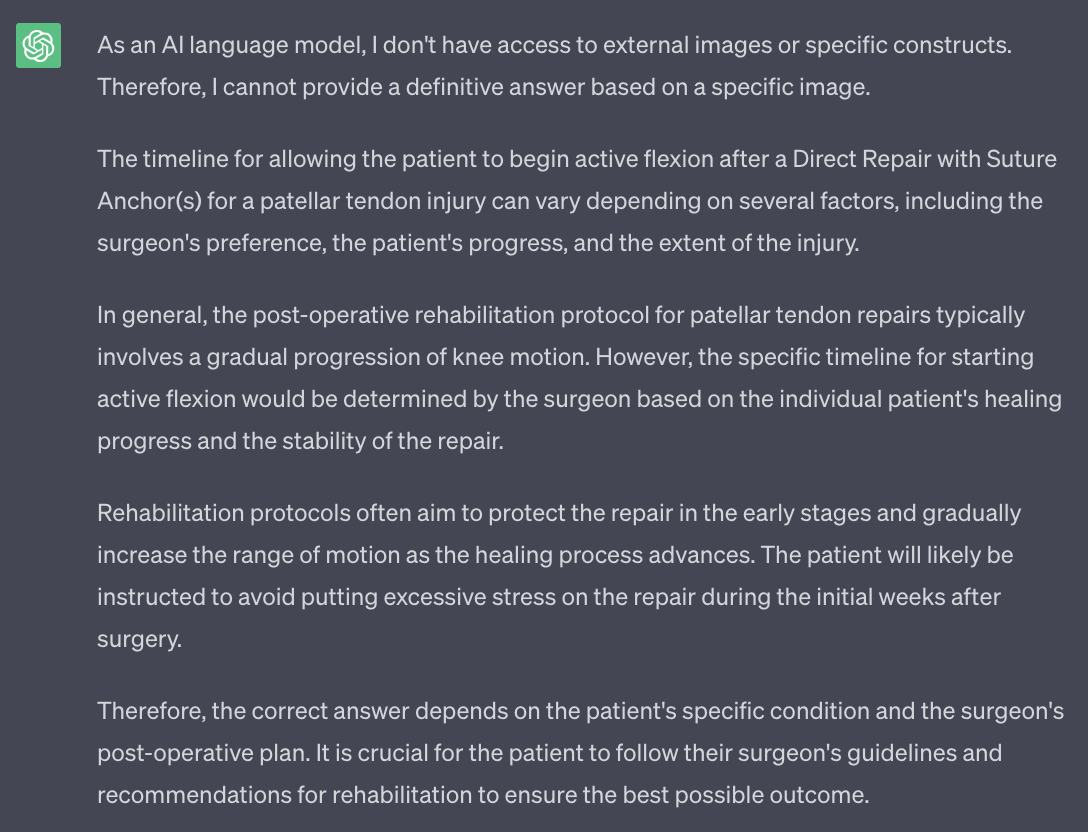


Q11: If you choose Direct Repair with Suture anchor(s) and obtained the construct below, when would you allow the patient to begin Active Extension?

- I would not choose Suture anchor repair
- < 2 weeks
- 2-4 weeks
- 5-6 weeks
- 7-8 weeks
- 9-12 weeks
- >12 weeks


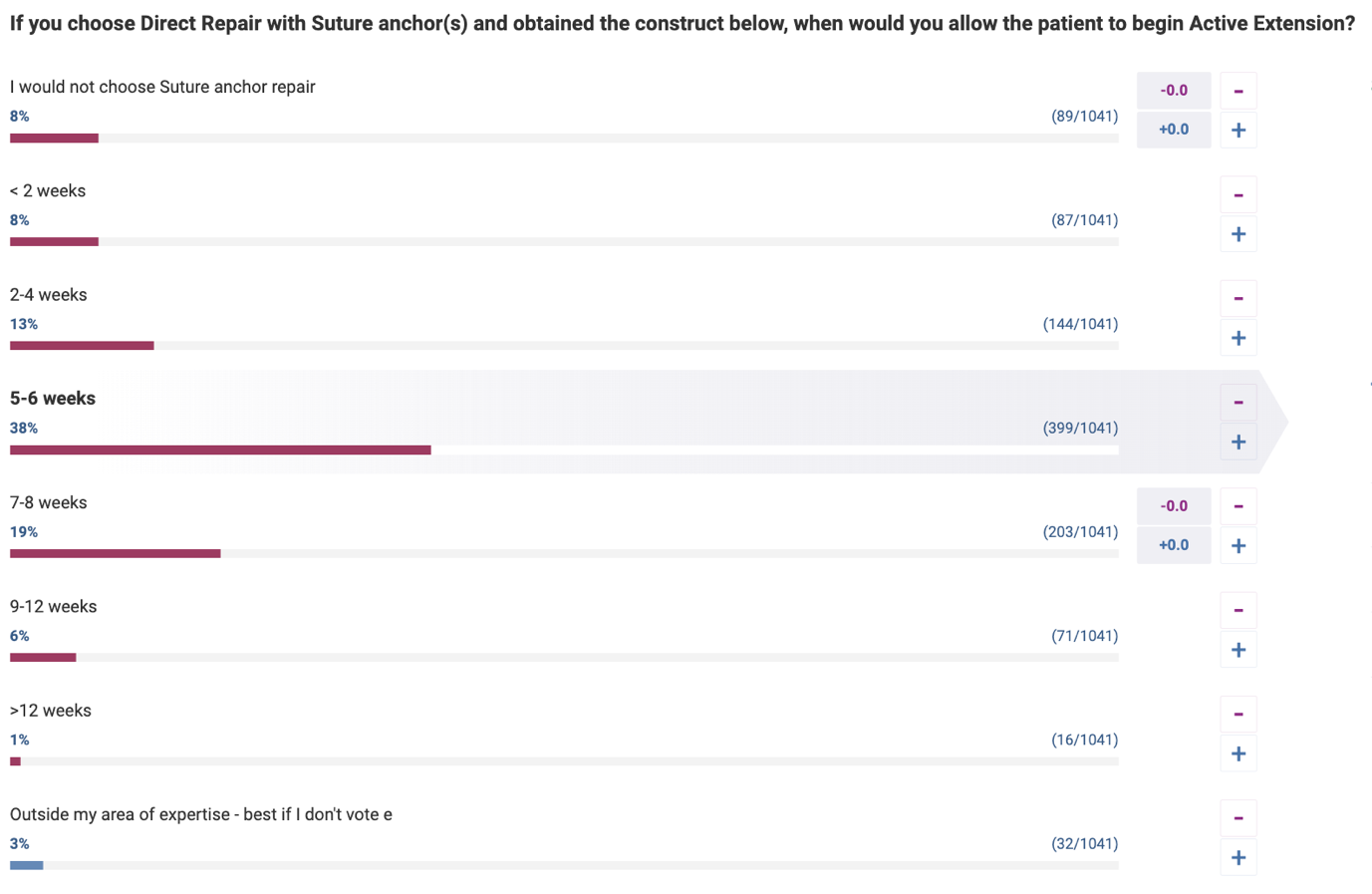


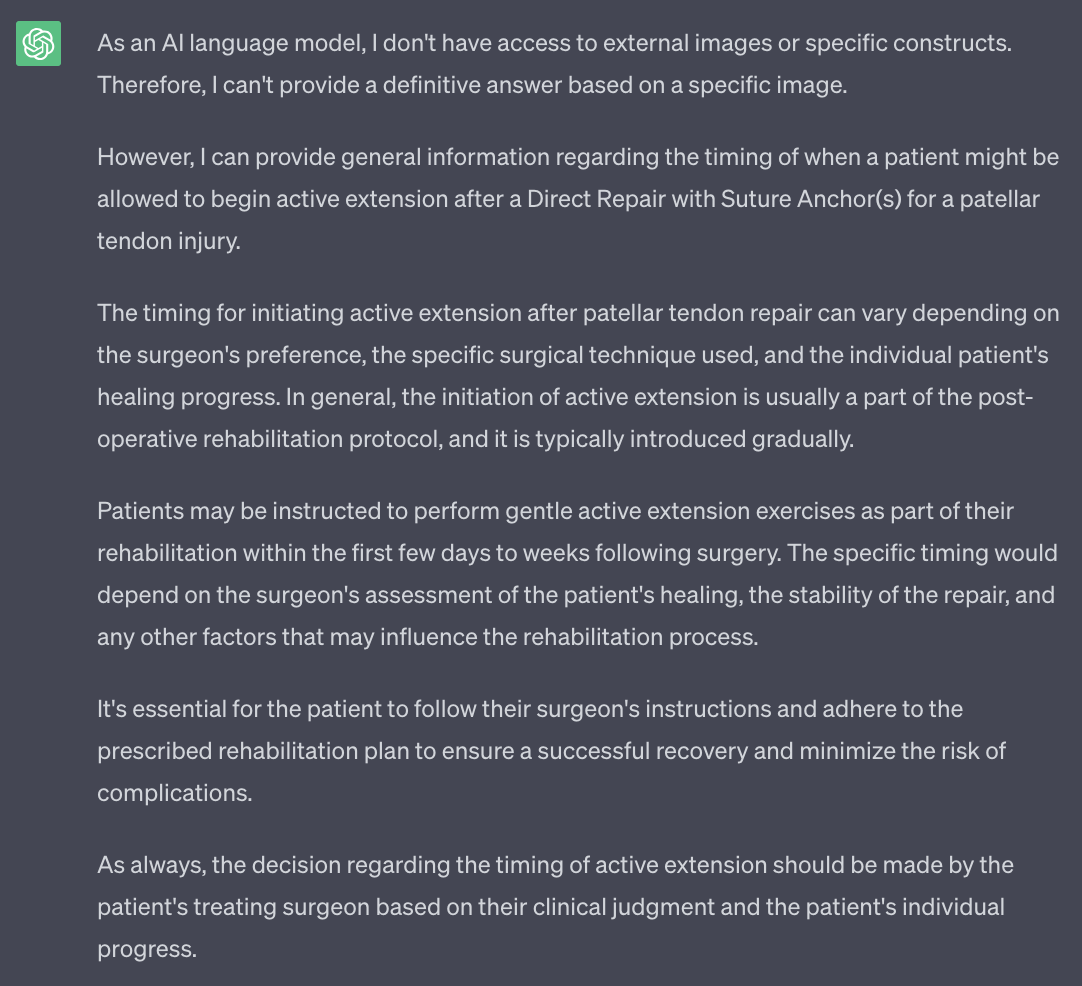


Q12: If you choose Direct Repair with Suture anchor(s) and obtained the construct (Fixation with suture anchors into the distal pole of the patella) below, when would you allow this patient to Return to Full Sport assuming he progressed through an active extension rehab protocol appropriately without complications?

- I would not choose Suture anchor repair
- Immediately
- At ~ 1 month
- At ~ 3 months
- At ~ 6 months
- At ~ 12 months
- At ~ 18 months or greater
- Never - no Full Return to Sports


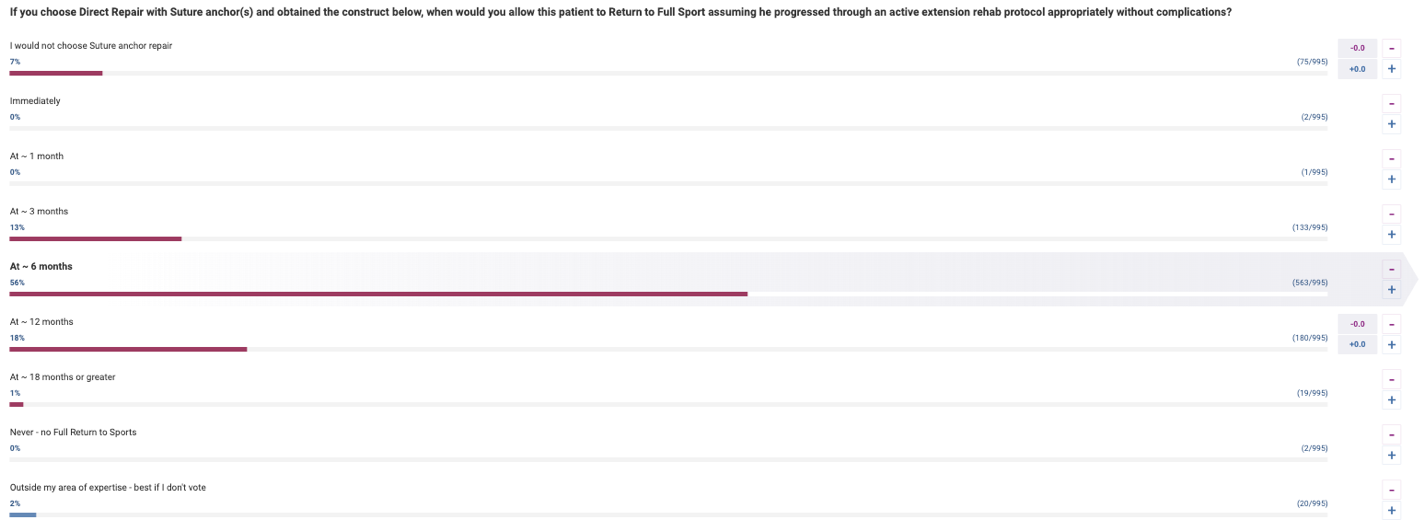


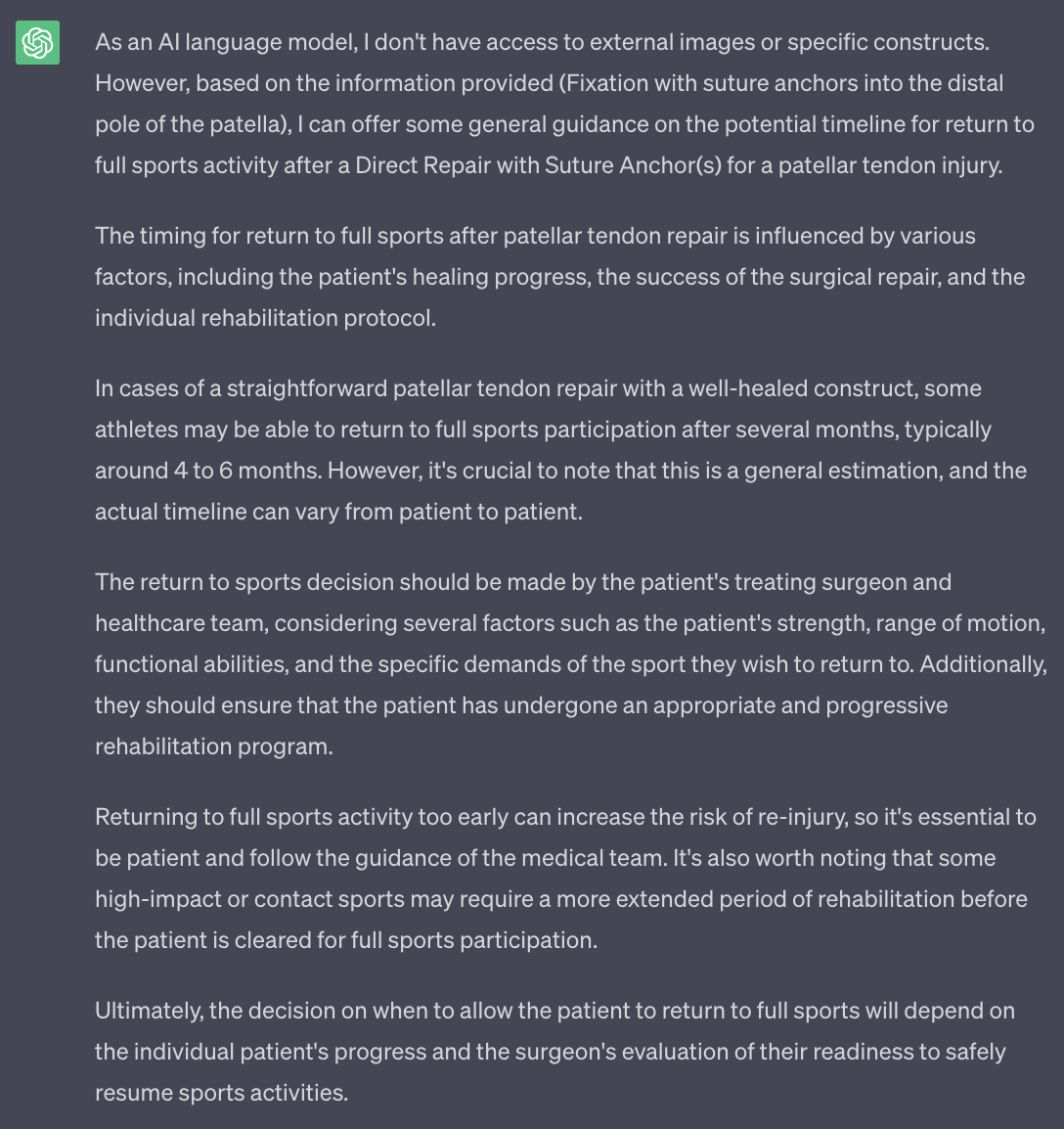


# Title: Supracondylar Humerus Fx with Pulseless Hand in 9F

Category: Paediatric

Popularity: 1

Date: 20230724

Link: <https://www.orthobullets.com/Site/Cases/View/d1b90b58-9732-403b-b08b-3924afa9baa4>

Images:
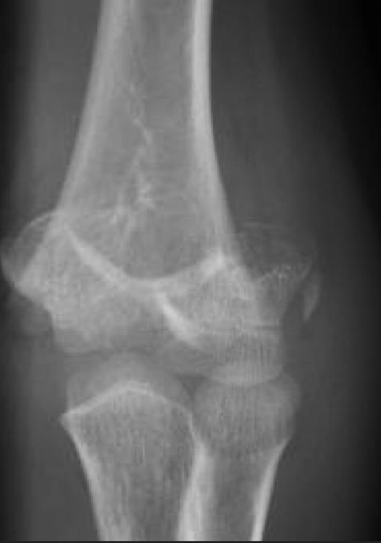

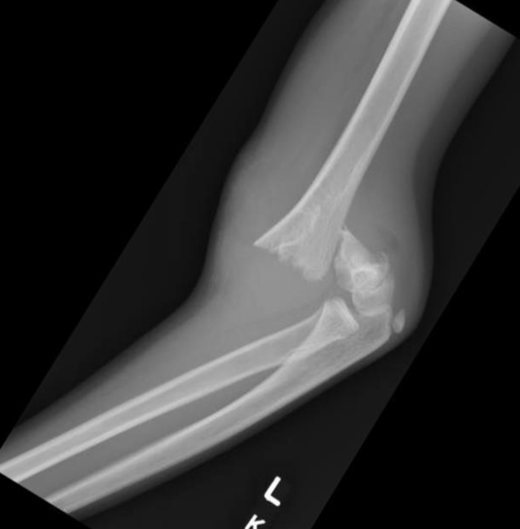


ChatGPT-3.5 dialogue:


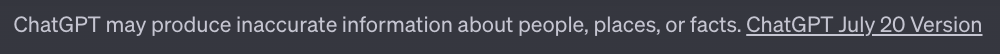


I am going to provide you a clinical vignette. There will be a series of 12 questions to follow. Please choose the single best response for each question.

History of presenting incident:

A 9-year-old female fell from a scooter onto her left hand and presents with severe left elbow pain

Past medical history:

Her past medical history is unremarkable.

Physical exam:
On physical exam her skin is intact. There is gross deformity, moderate swelling, and tenderness about left elbow, AIN/PIN and ulnar motor function are intact but there is a slight decrease in sensation in the median nerve distribution compared to the contralateral side. The hand is pink and well-perfused but there is no pulse.

Imaging findings:

Plain AP and lateral x-ray radiographs show a Gartland 3 left supracondylar fracture.

Q1: Would you obtain additional imaging to guide management?

- No - Current xrays are sufficient
- Yes - Additional xray views only (e.g., ipsilateral wrist, contralateral elbow)
- Yes - Angiogram only
- Yes - MRI only
- Yes - Additional xrays + MRI
- Yes - Additional xrays + Angiogram
- Yes - Additional xrays + Angiogram + MRI


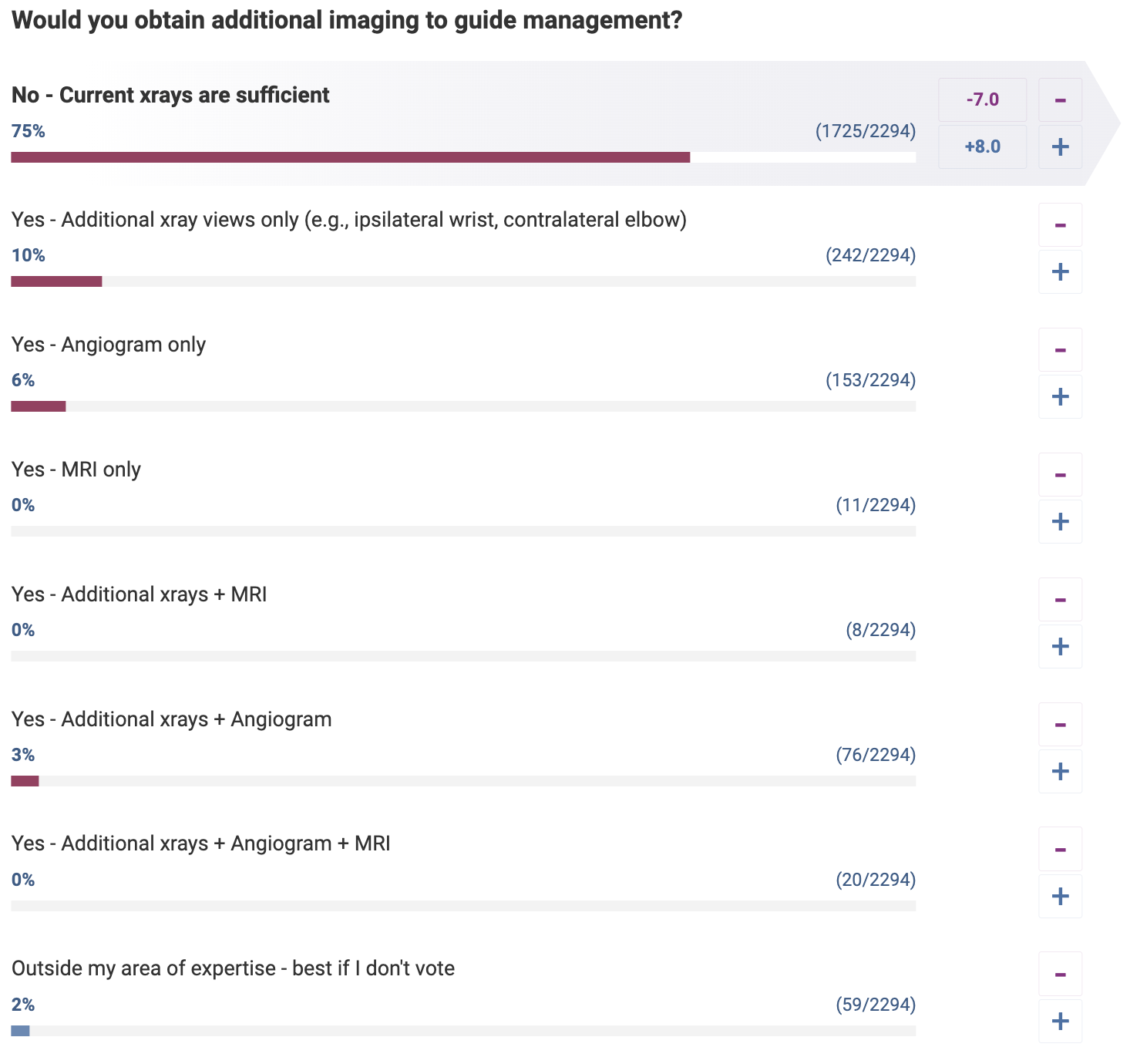


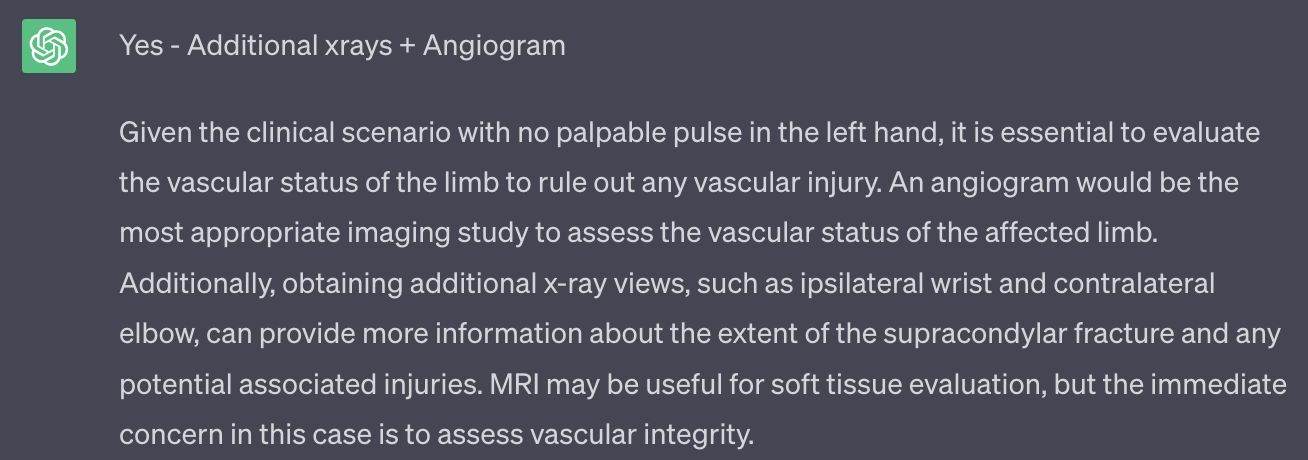


Q2: How would you classify this injury?

- I would not classify - it does not impact my treatment
- Gartland Type II
- Gartland Type III
- Gartland Type IV
- Flexion type


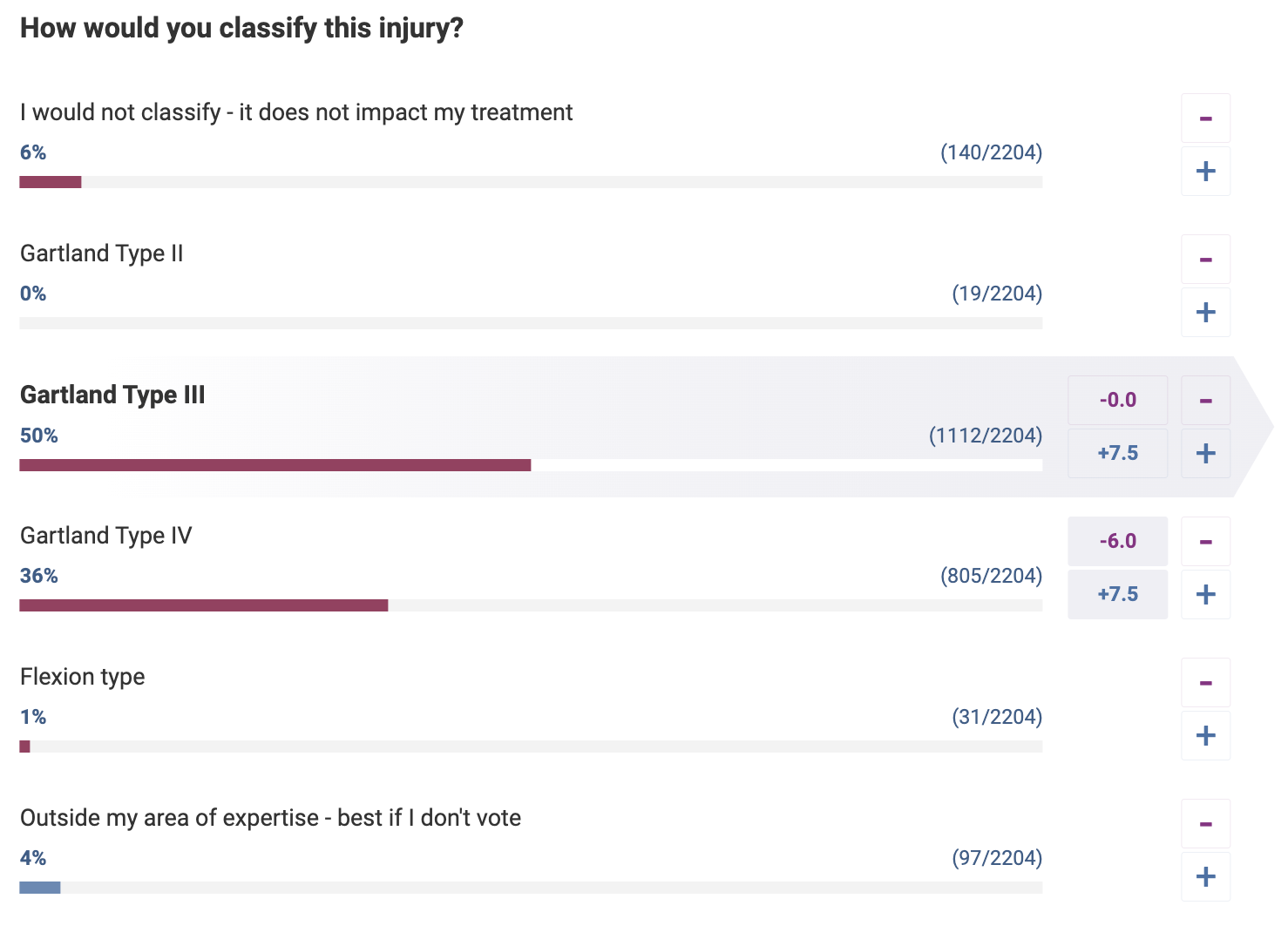


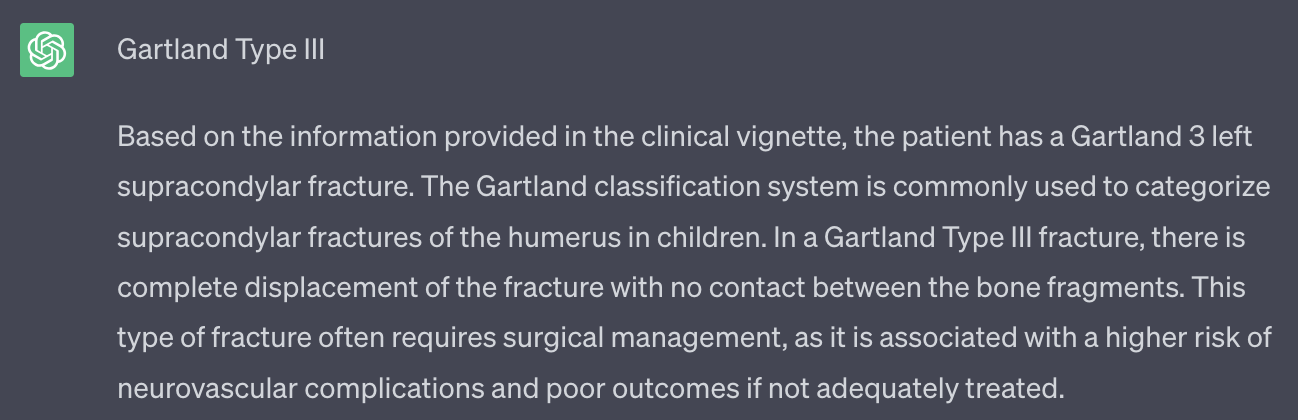


Q3: How would you initially manage this injury?

- Nonoperative (closed reduction, immobilization, any location)
- Operative


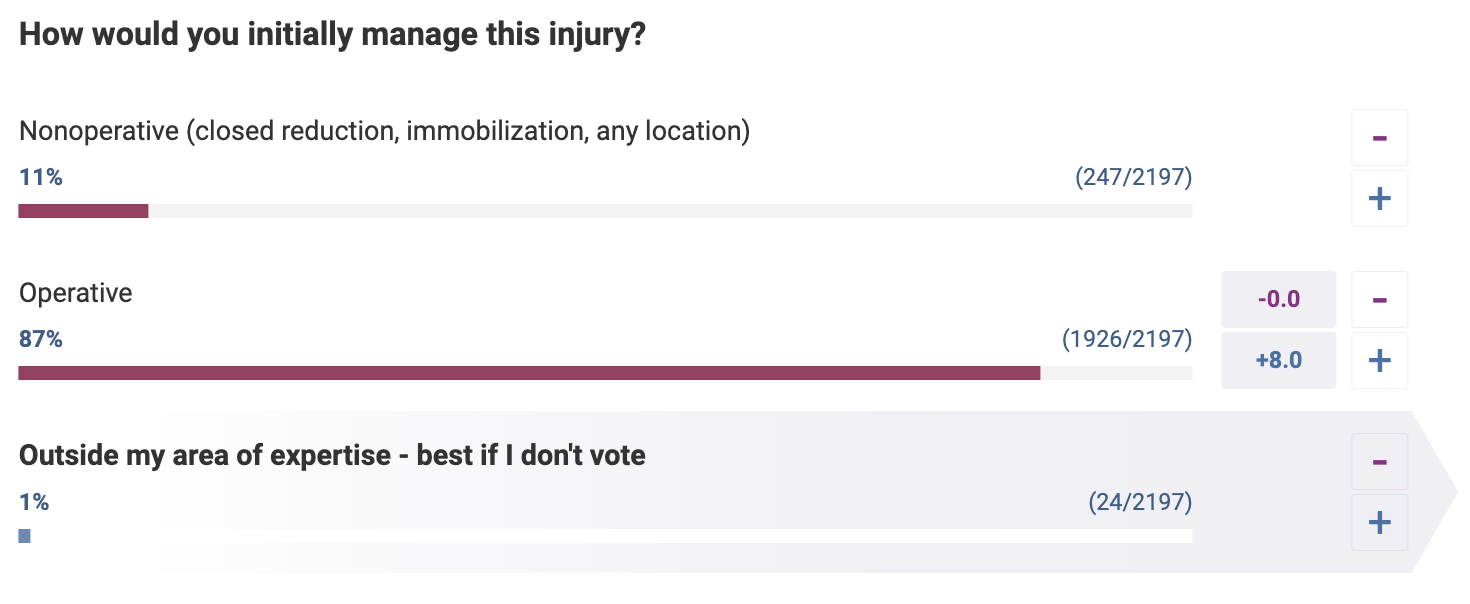


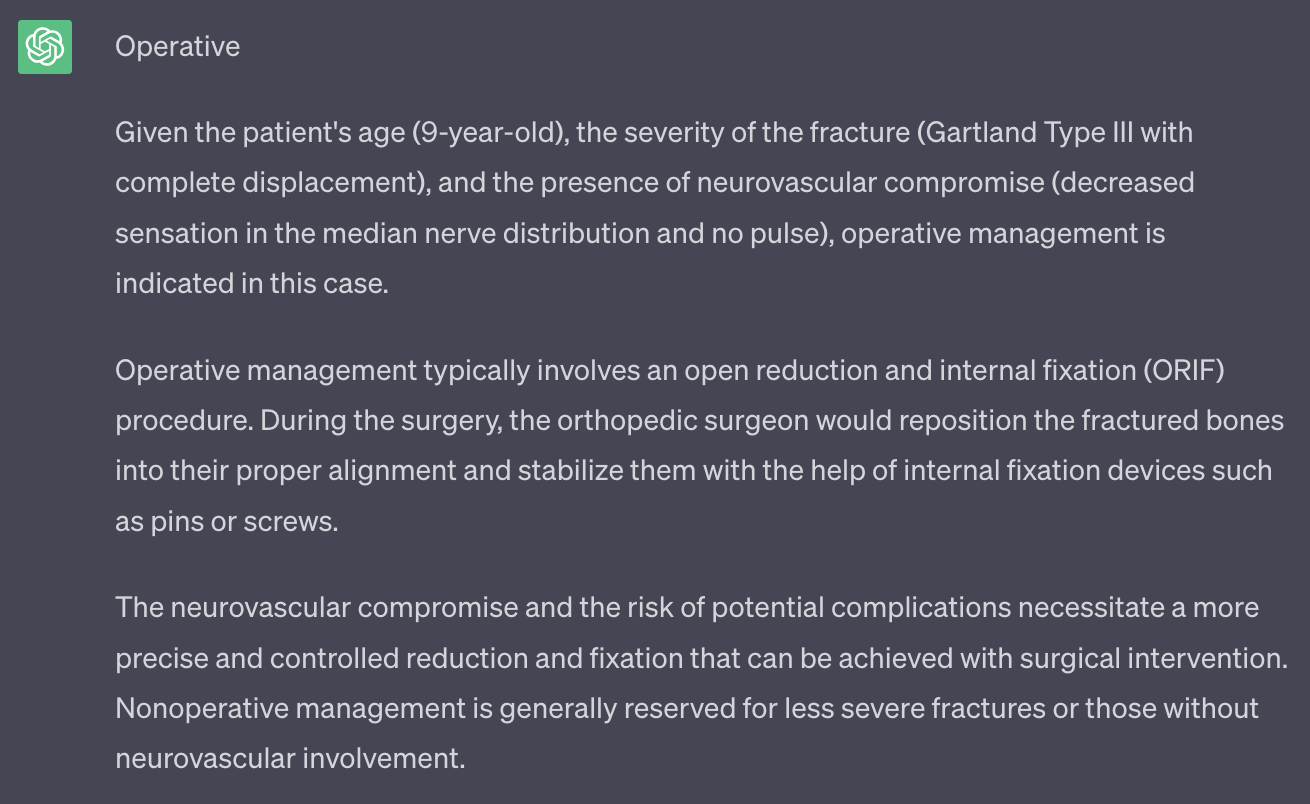


Q4: If you choose Operative management, assuming you saw the patient in the ER at 8 pm, and they ate a full meal at 7 pm, when would you perform surgery?

- I would not choose Operative management
- Emergently (within 2-3 hours)
- Urgently (that evening, I would NOT wait until following morning)
- Semi-urgently (first case following morning)
- Routine (follow-up in clinic to schedule surgery)


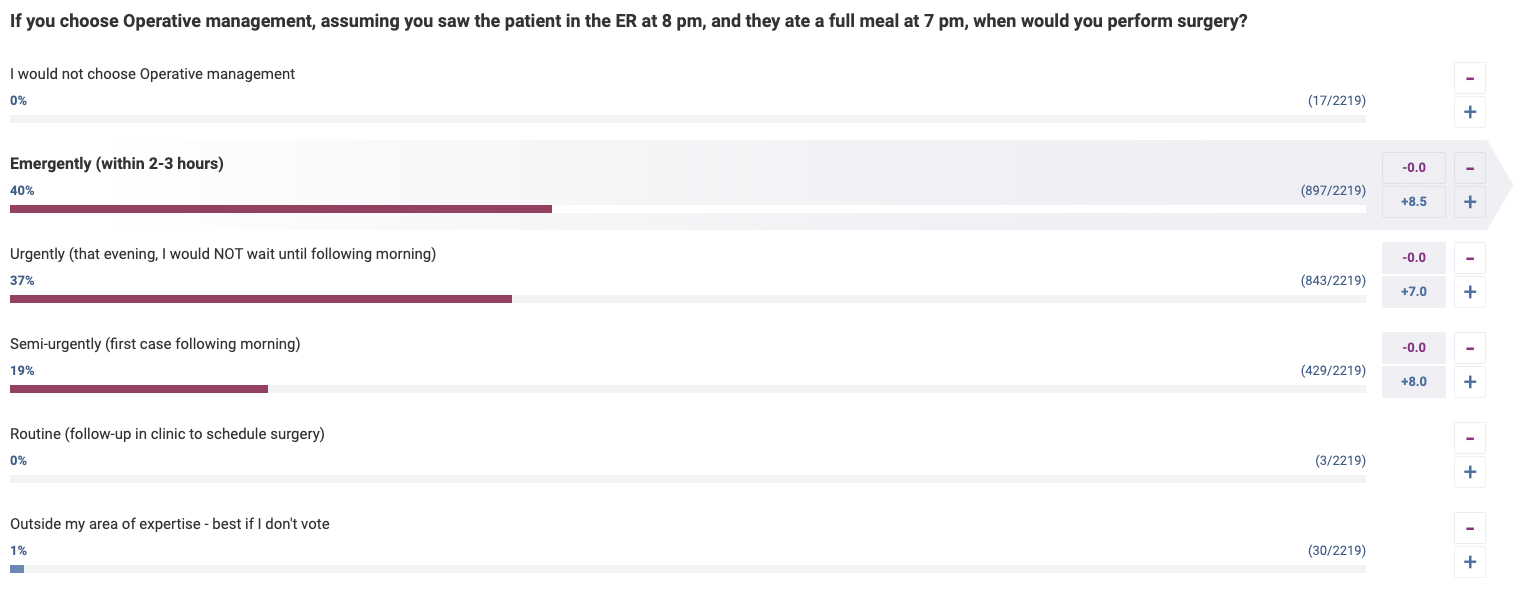


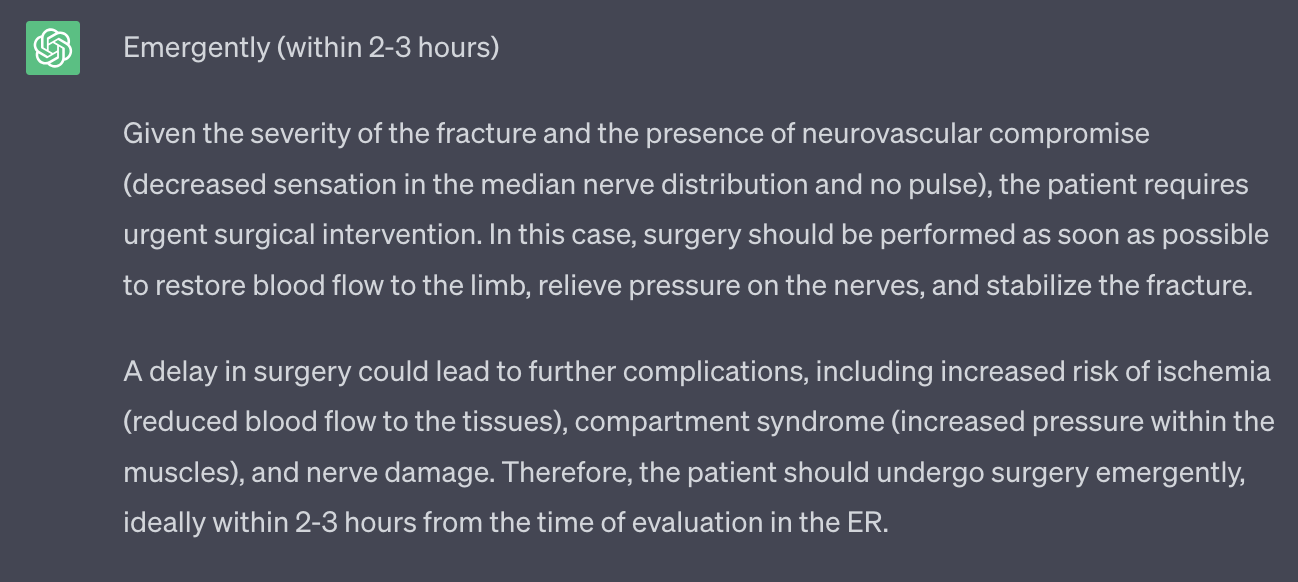


Q5: If you choose Operative management, what would be your plan going into surgery?

- I would not choose Operative management
- Attempt Closed reduction percutaneous pinning (CRPP), convert to open only if needed
- Planned Open reduction percutaneous pinning (ORPP), don't attempt CRPP)


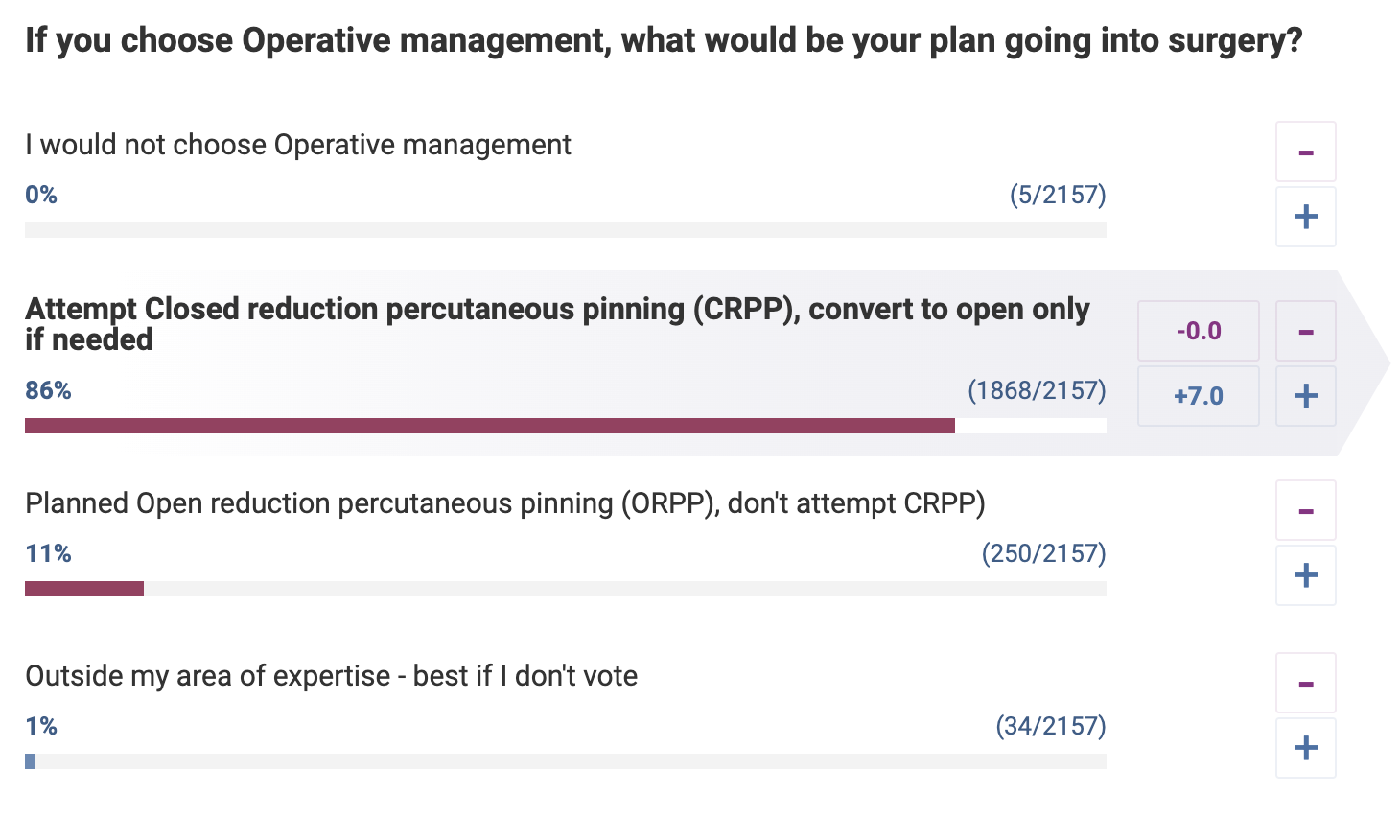


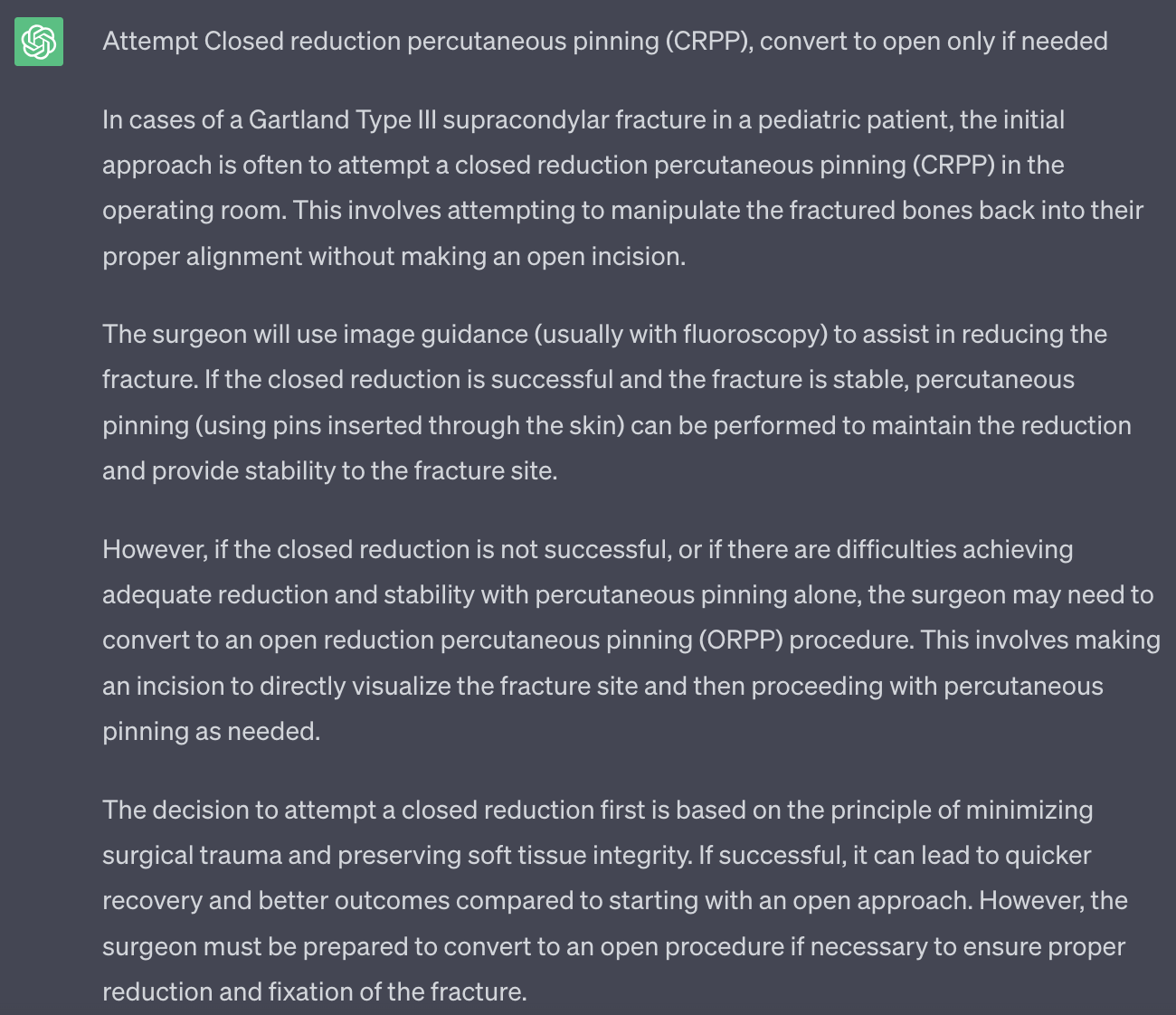


Q6: If you choose Operative management and ended up doing an Open reduction percutaneous pinning (ORPP), what approach would you use?

- I would not choose ORPP
- Anterior
- Lateral
- Medial
- Posterior


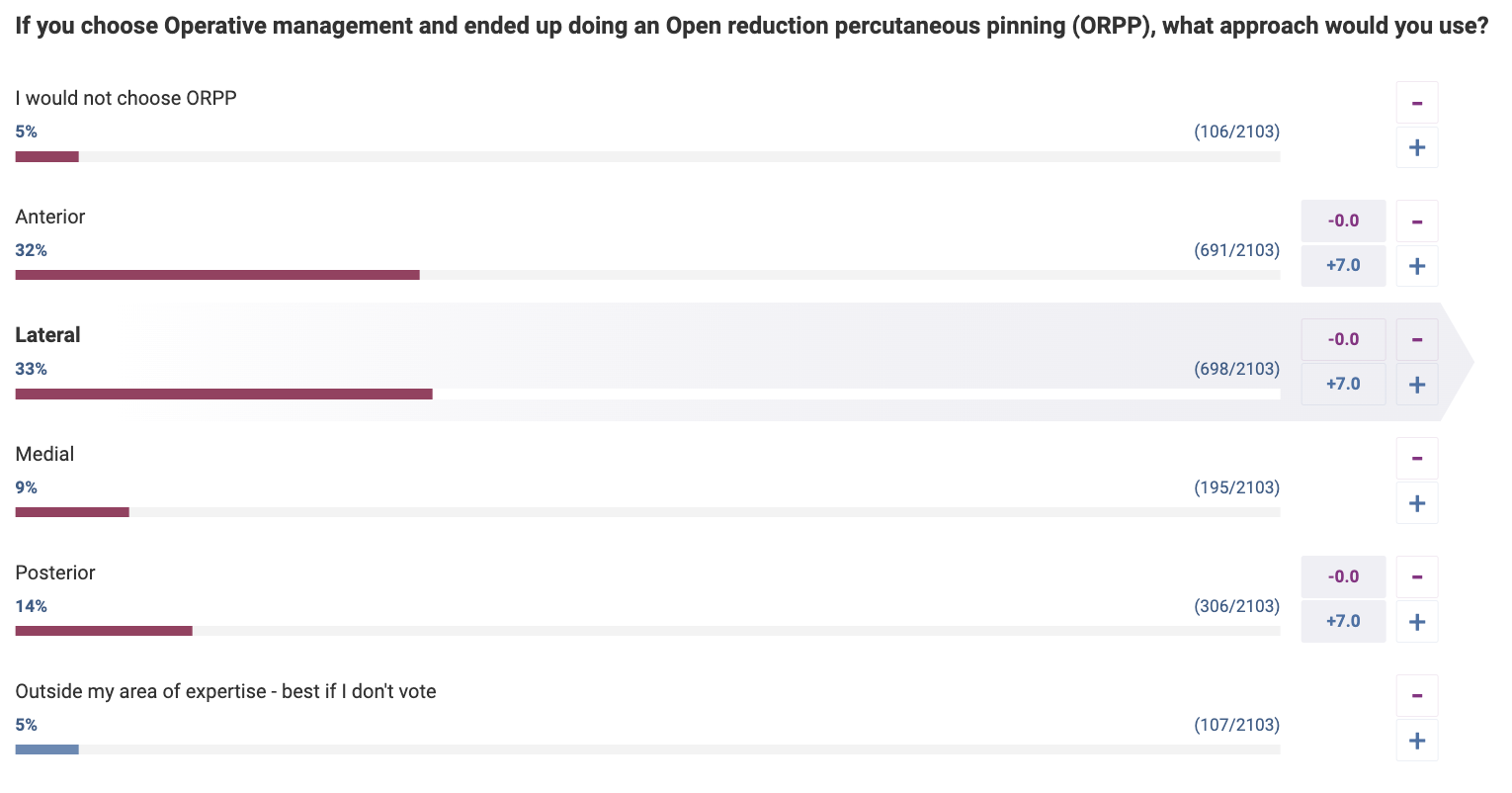


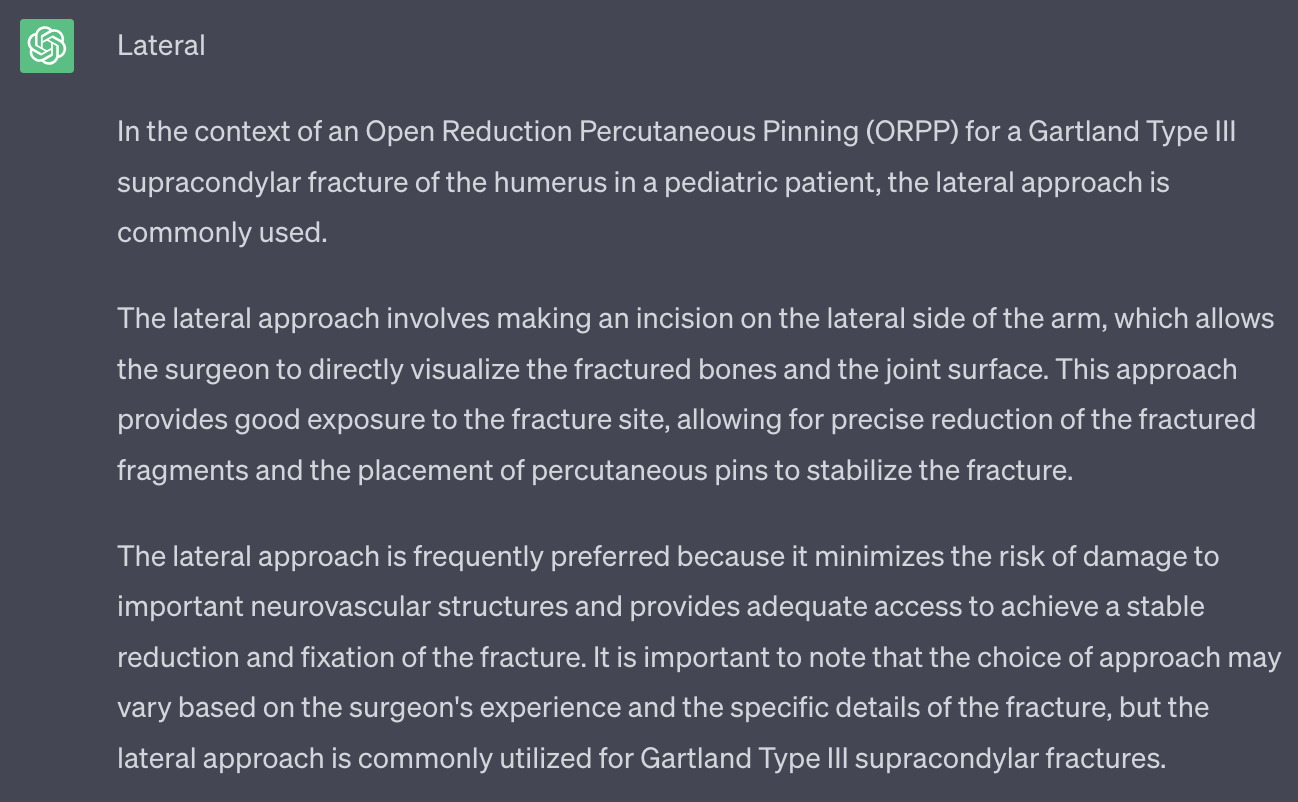


Q7: If you choose Closed reduction percutaneous pinning (CRPP) or Open reduction percutaneous pinning (ORPP), what fixation construct would you use?

- I would not choose CRPP or ORPP
- Lateral only - 2 pins
- Lateral only - 3 pins
- Combined - 1 lateral and 1 medial pin
- Combined - 2 lateral and 1 medial pins
- Combined - 3 lateral and 1 medial pins
- Combined - 1 lateral and 2 medial pins
- Medial only - 2 pins
- Medial only - 3 pins


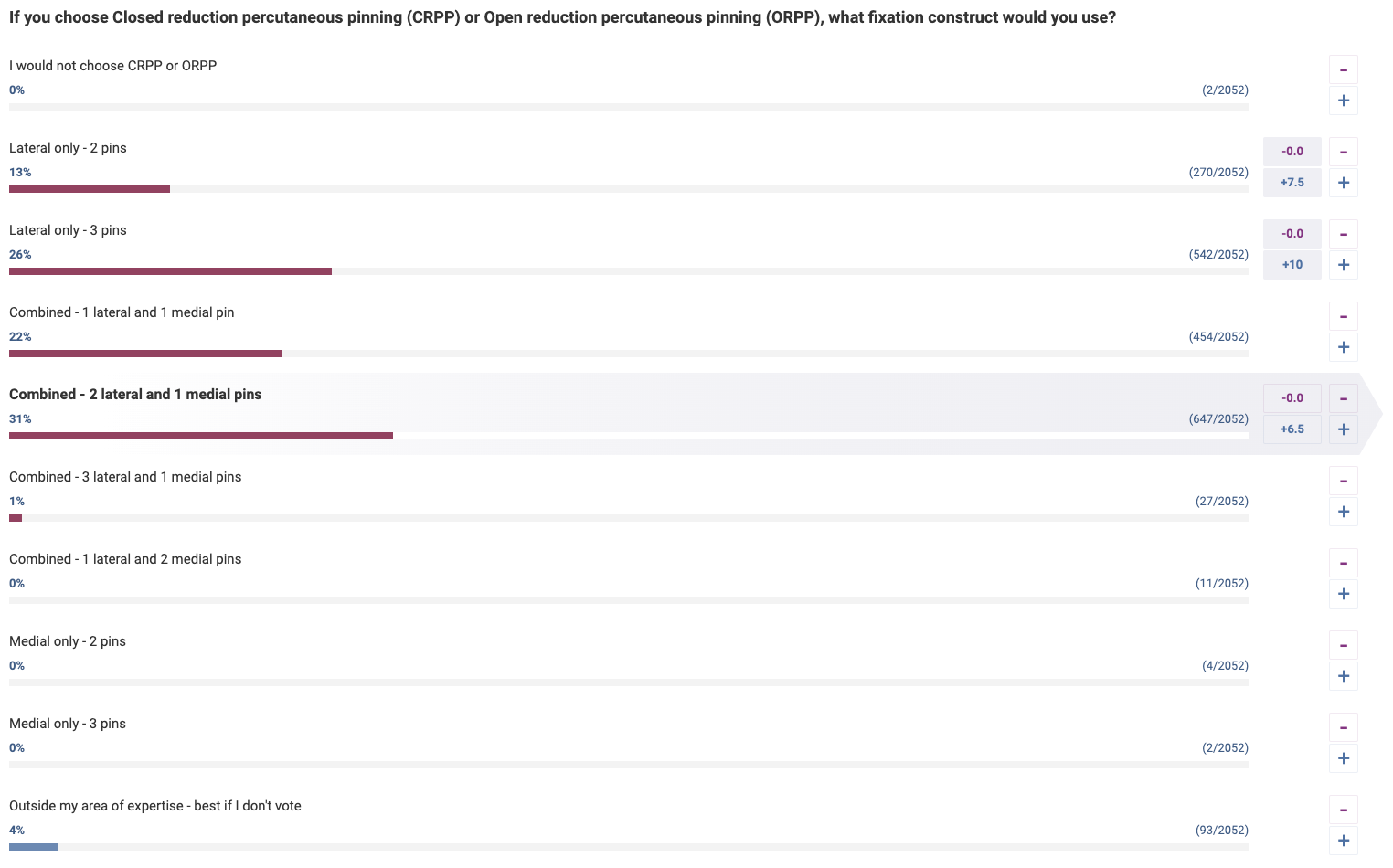


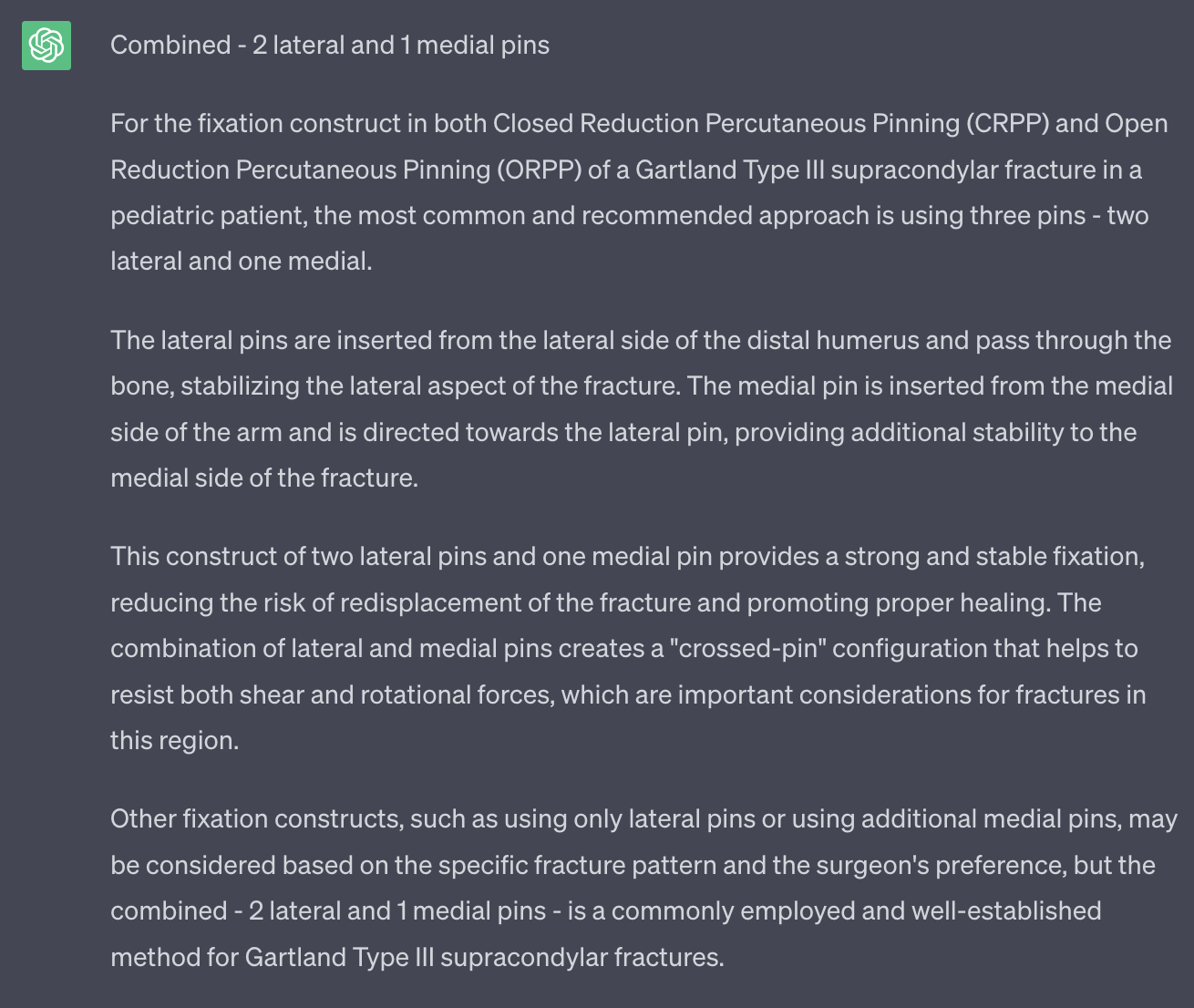


Q8: Assuming you performed Closed reduction percutaneous pinning (CRPP), and the patient has a GOOD PULSE postoperatively, how long would you observe this patient as an inpatient?

- I would not choose Operative management
- Discharge immediately
- Observe 0-12 hours, then discharge
- Observe 13-24 hours, then discharge
- Observe 25-48 hours, then discharge
- Observe > 48 hours, then discharge


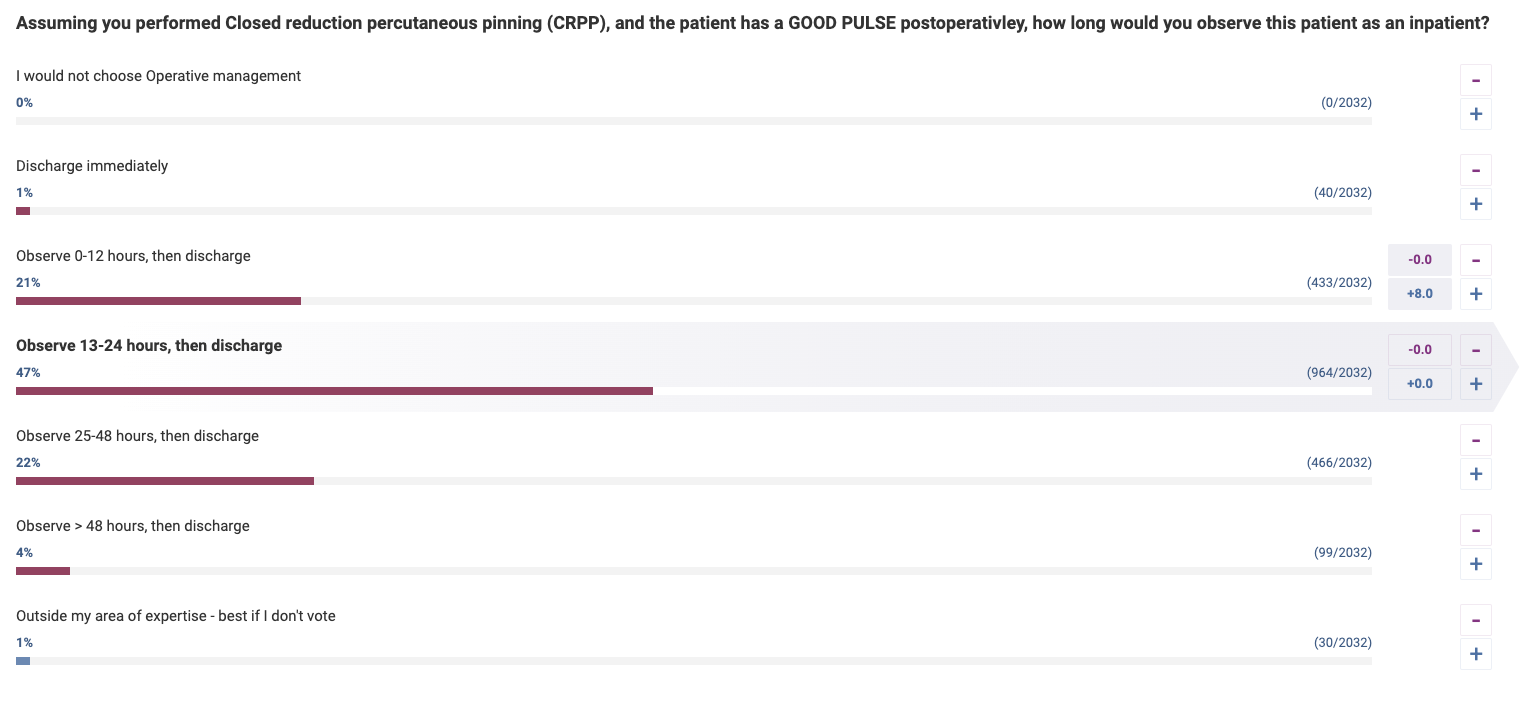


Q9: Assuming you performed Closed reduction percutaneous pinning (CRPP), and the patient had a PULSELESS PINK hand after pins are placed, how would you manage it?

- I would not perform CRPP
- Obtain intraoperative vascular consult
- Conclude case, observe inpatient for at least 24 hours - return to OR with vascular if pulse does not return
- Conclude case, observe inpatient for at least 48 hours - return to OR with vascular if pulse does not return
- Conclude case, observe inpatient for at least 72 hours - return to OR with vascular if pulse does not return
- Conclude case, observe inpatient for at least 24, send home as long as hand remains pink and family is reliable
- Conclude case, observe inpatient for at least 48, send home as long as hand remains pink and family is reliable
- Conclude case, observe inpatient for at least 72, send home as long as hand remains pink and family is reliable

Q10: If you choose Closed reduction percutaneous pinning (CRPP) or Open reduction percutaneous pinning (ORPP) in this 9yoF, when would you remove the pins?

- I would not choose CRPP or ORPP
- at ~ 2 weeks (14 days postop)
- at ~3 weeks (21 days postop)
- at ~ 4 weeks (28 days postop)
- at 5-7 weeks (35 - 49 days postoop)
- > 7 weeks

Q11: If you choose Closed reduction percutaneous pinning (CRPP) with construct seen in xrays below (well reduced, three diverging K-wires inserted from the lateral condyle), assuming radiographic union at the time pins and cast removed, when and how would you begin ROM exercises?

- I would not choose CRPP
- No ROM exercise are needed. Motion will come back on its own.
- I would instruct Family to begin ROM Immediately
- I would prescribe a Physical therapist to begin ROM immediately
- I would instruct Family to begin ROM starting in several weeks
- I would prescribe a Physical therapist to begin ROM starting in several weeks

Q12: If you choose Closed reduction percutaneous pinning (CRPP) with construct seen in xrays below (well reduced, three diverging K-wires inserted from the lateral condyle), assuming radiographic union at the time pins and cast removed, when would you allow the child to return to sports?

- Immediately after the pins and cast removed
- 2 weeks after the pins and cast removed
- 4 weeks after the pins and cast removed
- 8 weeks after the pins and cast removed
- Once they have regained full range of motion

# Title: Periprosthetic Hip Fx in 86F

Category: Reconstruction

Popularity: 1

Date: 20230724

Link: <https://www.orthobullets.com/Site/Cases/View/03fd8484-55e8-42bd-9607-6d81a573a9cc>

Images:

ChatGPT-3.5:

I am going to provide you a clinical vignette. There will be a series of 11 questions to follow. Please choose the single best response for each question.

History of presenting incident:

Patient underwent hybrid right hip replacement in 2014. She slipped and fell at a train station onto her right side and had acute right groin/thigh pain.

Past medical history:

Hypothyroidism

Vertigo

Appendectomy

Total Shoulder Replacement

Physical exam:
Right leg short and externally rotated, pain with logrolling.

Remainder of secondary survey: negative

Neurologically intact

Palpable pulses.

Imaging findings:

Plain AP and lateral x-ray radiographs show a likely Vancouver B2 right hip periprosthetic fracture. Previous cemented right total hip arthroplasty is noted.

Q1: What additional studies, if any, would you get to determine treatment?

- None - current Xrays enough
- CT Scan
- MRI

Q2: Would you obtain the old operative report to identify the type of stem?

- No - it is not important and I dont need it
- Yes - it is somewhat important, and if easily obtained I will get it.
- Yes - it is very important, and I will put significant effort into getting it.

Q3: How would you classify this with the Vancouver Classification System?

- I would not classify it with the Vancouver System - it does not help me
- A1
- A2
- A3
- B1
- B2
- B3
- C1
- C2
- C3

Q4: What would be your preferred treatment plan?

- ORIF Only (retain femoral component)
- ORIF and Revision of Femoral Component
- ORIF and Revision of Femoral and Acetabular Component
- I would not operate on this patient

Q5: If choosing to perform ORIF Only, what would you use as your construct for the proximal femur?

- Cables
- Plate with Screws
- Cables AND Plate with Screws
- I would not perform ORIF of the proximal femur

Q6: If choosing Revising the Femoral Component, what implant would you switch to?

- Long cylindrical stem
- Long Wagner (tapered) stem
- Modular diaphyseal fitting stem
- I would not revise the femoral component

Q7: If choosing Revising the Femoral Component, what prosthesis fixation technique would you use?

- Leave Existing Cement - and cement within (cement within cement)
- Remove old cement - add New Cement
- Remove old cement - Cementless Fixation

Q8: If you choose revision of the femoral component with Cementless Fixation, following the cement removal, how would you ream the distal femur?

- Distract the fracture site, Ream Distally First, then perform ORIF
- Reduce the fracture and place definitive fixation, the ream from the top
- Reduce the fracture and obtain provisional fixation, the ream from the top, then perform definitive fixation

Q9: If using an extensile approach to perform ORIF what would be your first operative step?

- Provisionally clamp/fix the fracture, then enter the hip joint.
- Complete the approach to the hip joint first, then assess the fracture
- Distract open the fracture to assess stem stability, remove cement mantle
- Perform a femoral osteotomy to facilitate stem removal
- Other option

Q10: When you observe a periprosthetic fracture extending into good-quality cement mantle (with no subsidence), what radiographic or stem variable do think is MOST important to lead to stem loosening?

- Whether there is a Collar (composite beam type) or no collar (Exeter type)
- Surface Finish of the stem
- Fracture Displacement >2mm
- No one variable is most important, there are multiple factors

Q11: Assuming you achieve rigid fracture fixation and stem stability intraoperatively with the construct seen in the postop films below (Implant retention, open reduction with internal fixation using 6x metallic cables only), what would be your postoperative weight bearing status?

- Weightbearing as tolerated with assistive device x 6 weeks
- 50% weightbearing with assistive device x 6 weeks
- Toe touch weight bearing for 6 weeks
- Toe touch weight bearing for 3 months
- Non weight-bearing for 6 weeks
- Other option

# Title: Anterior Shoulder Instability with Bipolar Bone Loss in 38F

# Category: Shoulder and elbow

Popularity: 1

Date: 20230723

Link: <https://www.orthobullets.com/Site/Cases/View/ea5628c1-185e-4b32-be92-b4de07519945>

Images:

ChatGPT-3.5 dialogue:

I am going to provide you a clinical vignette. There will be a series of 13 questions to follow. Please choose the single best responses for each question.

History of presenting incident:

A 38 year-old female with epilepsy presents with progressively worsening left shoulder pain and recurrent instability. She sustained her first left shoulder dislocation during a seizure two years prior to presentation. She was treated nonoperatively while initiating anti-epileptic medication. Three months ago, she sustained a traumatic shoulder dislocation during a fall off of a bicycle. Subsequently, she has experienced multiple dislocations per week with minimal-to-no trauma. She has not suffered a seizure in more than two years.

Past medical history:

Epilepsy, hypothyroidism, ADHD

Physical exam:

On physical exam, the patient exhibits significant apprehension in the mid-arc of motion, therefore full range of motion testing is deferred. Rotator cuff strength is fully intact against manual resistance testing. The patient’s Beighton score is measured as 6/9. The neurovascular exam is normal.

Imaging findings:

Plain (axillary lateral, grashey, and outlet) x-ray radiographs of the right shoulder show subtle signs of a hill-sachs lesion. This is confirmed on MRI scan which shows a hill-sachs lesion (max width 24mm) with shoulder effusion. Furthermore, there is mild to moderate bone loss on the glenoid side. Rotator cuffs are intact.

Q1: In addition to AP and LAT radiographs of the shoulder, would you obtain any other imaging to guide management?

- No - AP and LAT radiographs of the Shoulder are sufficient
- Yes - additional radiographic views (aXR)
- Yes - Shoulder CT (CT)
- Yes - Shoulder MRI (MRI)
- Yes - aXR + CT
- Yes - aXR + MRI
- Yes - CT + MRI
- Yes - aXR + CT + MRI
- Yes – Other

Q2: Do you routinely calculate whether a Hill-Sachs lesion is “on-track” or “off-track”? If so, which is your preferred advanced imaging modality for performing this calculation?

- No, I do not perform this calculation routinely
- Yes, I prefer to perform this calculation based on CT measurements
- Yes, I prefer to perform this calculation based on MRI measurements
- Yes, and I have no preference on making measurements on CT or MRI

Q3: How would you manage this patient at this time?

- Nonoperative
- Operative

Q4: If you choose Operative management, how long would you want the patient to be “seizure free” before you operate?

- I would not choose Operative Management
- No minimum timeframe
- >6 weeks
- >3 months
- >6 months
- >1 year

Q5: If you choose Operative Management, what treatment would you perform?

- I would not choose Operative management
- Soft Tissue Procedure (STP) Alone +/- remplissage
- Bony Bankart Repair (BBR) Alone +/- remplissage
- Bone Reconstruction (Recon) Alone (glenoid, humerus, or both)(+/- remplissage)
- STP + BBR +/- remplissage
- STP + Recon (+/- remplissage)
- Other

Q6: If you choose a Bone Reconstruction Procedure (+/- remplissage), would you reconstruct the glenoid, humerus, or both?

- I would not choose a Bone Reconstruction procedure
- Glenoid Alone - (e.g. Latarjet or distal tibial allograft)
- Humerus Alone - (e.g. femoral head allograft, talar allograft)
- Combined Glenoid and Humeral head reconstruction

Q7: If you choose a Bone Reconstruction procedure, would you do it Open or Arthroscopically?

- I would not choose a Bone Reconstruction procedure
- Arthroscopic
- Open

Q8: If you choose an Open Bone Reconstruction procedure, what bone graft would you use on the glenoid?

- I would not choose an Open Bone Reconstruction procedure
- Autograft - Coracoid Transfer (Latarjet)
- Autograft – ICBG
- Autograft - Distal clavicle
- Autograft - Scapular Spine
- Autograft – Other
- Allograft - Distal tibia (DTA)
- Allograft – Other

Q9: If you choose an Open Bone Reconstruction Procedure, how would you obtain fixation of the bone graft on the glenoid?

- I would not choose an Open Bone Reconstruction procedure
- Cortical-button(s)
- Screw(s)
- Plate with screw(s)
- Other

Q10: If you choose an Open Bone Reconstruction Procedure on the glenoid, would you address the patient’s Hill-Sachs deformity?

- I would not choose an Open Bone Reconstruction Procedure on the Glenoid
- No
- Maybe - if the lesion is off-track on intraoperative examination
- Yes

Q11: If you choose and Open Bone Reconstruction procedure on the glenoid and to address the Hill-Sachs lesion, what would you do?

- I would not choose to address the Hills-Sachs lesion
- Remplissage procedure
- Osseous reconstruction – Autograft
- Osseous reconstruction – Allograft
- Rotational osteotomy

Q12: If you choose Osseous reconstruction with an Allograft of the Hill-Sachs lesion, what type of allograft would you use?

- I would not choose Osseous reconstruction with an Allograft of the Hill-Sachs lesion
- Talus
- Humeral head
- Femoral head
- Other

Q13: If you choose to perform the Open Bone Reconstruction Procedure shown (talar allograft fixed to hill-sachs lesion), how would you determine when the patient can return to contact sports?

[images not provided to ChatGPT]

- I would not choose an Open Bone Reconstruction procedure
- Imaging - only when Xray show graft incorporation
- Imaging - only when CT shows graft incorporation
- Time & exam - 2-3 months (don't get CT)
- Time & exam - 4-5 months (don't get CT)
- Time & exam - 8 or more months (don't get CT)

# Title: L2 Burst Fracture with Neurologic Deficits in 45F

Category: Spine

Popularity: 1

Date: 20230723

Link: <https://www.orthobullets.com/Site/Cases/View/05948bd6-348e-4938-bde7-d3d870e97cd0>

Images:

ChatGPT-3.5 dialogue:

I am going to provide you a clinical vignette. There will be a series of 13 questions to follow. Please choose the single best responses for each question.

History of presenting incident:

The patient is a 45-year-old female that fell off her horse yesterday while doing a recreational ride. Immediately after the fall, she had severe pain in her back, making it almost impossible for her to move. She was brought to the ER by EMT. Upon seeing the patient, she complained of pain in her neck, pain in her back, and numbness and tingling primarily in her right leg and bilateral buttocks, and a sensation of "heaviness" in her bilateral lower extremities. She denied abnormal sensations in her perineum.

Past medical history:

Factor V deficiency

Physical exam:

Inspection and palpation of the back shows TTP of TL region. No abrasions or lacerations.

She has decreased sensation in the region of the buttocks bilaterally. She has normal sensation in the perineum.

Rectal exam deferred.

LUE: Normal

RUE:

4 of 5 deltoid

4 of biceps

5 of 5 BR

5 of 5 Triceps

Neg Hoff

Examination of her RIGHT lower extremity shows:

~3-4 HF

4 of 5 KE

4 of 5 ADF

4- of 5 APF

Normal Patellar Reflexes

Neg Bab

Examination of her LEFT lower extremity shows:

~3-4 HF

4 of 5 KE

4 of 5 ADF

4+ of 5 APF

Normal Patellar Reflexes

Neg Bab.

Imaging findings:

- CT and MRI images are provided, showing an L2 burst fracture with retropulsion.

Q1: In addition to a CT chest/abdomen/pelvis ordered by the trauma team, what additional imaging studies would you obtain to dictate management?

- None
- Additional radiographs (aXR) (ie. Lumbar Flex and Ext)
- Fine Cut Lumbar CT (LCT)
- MRI Lumbar
- aXR + LCT
- LCT + MRI
- aXR + MRI
- aXR + LCT + MRI

Q2: Would you use a classification system for this injury to guide management?

- No - a classification system would not help me
- Yes – Dennis
- Yes – TLCIS
- Yes – Other

Q3: How would you manage this fracture?

- Nonoperative
- Operative

Q4: The accident occurred around 5 pm in the afternoon. The MRI was not completed until 10 pm. If you choose Operative management when would you do the case?

- I would not choose Operative management
- Same night (middle of night)
- First case following morning - bump other scheduled cases
- Add case to end of following day schedule (~ 4pm following day)
- Next open OR slot within 48 hours

Q5: If you choose Operative Management, what approach would you use?

- I would not choose Operative management
- Anterior Only (includes direct lateral)
- Posterior Only
- Combined Anterior + Posterior

Q6: If you choose a Posterior Only Procedure, what would you do?

- I would not choose a Posterior Only procedure
- Instrumented Fusion Only (no decompression)
- Decompression Only (no fusion)
- Decompression + Instrumented Fusion

Q7: If you choose a Posterior Decompression + Fusion, how would you perform the decompression?

- I would not perform a Posterior Decompression + Fusion
- L2 Laminectomy Only
- L2 Laminectomy + Retropulsed Fracture Reduction (push anterior)
- L2 Laminectomy + L2 Corpectomy + Cage Placement from Posterior

Q8: If you choose a Posterior Decompression & Fusion with L2 laminectomy +/- fracture reduction, what levels would you instrument to?

- I would not perform a Posterior Decompression & Fusion with L2 laminectomy
- One Above/Below (L1-L3 PSF)(Short Segment)
- Two Above/Below (T12-L4 PSF)
- Three Above/Below (T11-L5 PSF)

Q9: If you choose a Posterior Decompression & Fusion and instrumented Two Above/Below (T12-L4 PSF), what levels would you actually fuse (decorticate bone) vs. instrument?

- I would not perform Posterior Decompression & Fusion
- No fusion, only instrumentation
- "Fuse Short (Decorticate L1-3) Instrumented Long" (T12-L4)
- "Fuse Long" (Decorticate from T12-L4)

Q10: If you choose to Fuse Short (L1-3) Instrumented Long (T12-L4) with Percutaneous Screws, would you remove your hardware?

- I would not Fuse Short (L1-3) Instrumented Long (T12-L4) with Percutaneous Screws
- No - leave all screws in forever unless symptomatic
- Yes - Remove Distal screws Only (T12, L4, cut rods) at 3 months
- Yes - Remove Distal screws Only (T12, L4, cut rods) at 6 months
- Yes - Remove Distal screws Only (T12, L4, cut rods) at 12 months
- Yes - Remove All Screws and Rods at 3 months
- Yes - Remove All Screws and Rods at 6 months
- Yes - Remove All Screws and Rods at 12 months

Q11: If performing Posterior instrumentation 2 levels above and below, what pedicle screw technique would you use?

- I would not instrument posterior 2 levels above and below
- Percutaneous Screws - Using Fluoro
- Percutaneous Screws - Using Intraoperative CT Navigation (no robot)
- Percutaneous Screws - Using Navigated Robot
- Open Screws - Using Anatomic Technique
- Open Screws - Using Fluoro
- Open Screws - Using Intraoperative CT Navigation (no robot)
- Open Screws - Using Navigated Robot

Q12: If you choose an Anterior-Posterior Procedure with an Anterior L2 Corpectomy and Cage, what levels would you instrument to posteriorly?

- I would not perform an Anterior-Posterior Procedure with an Anterior L2 Corpectomy and Cage
- One Above/Below (L1-L3 PSF)
- Two Above/Below (T12-L4 PSF)
- Three Above/Below (T11-L5 PSF)

Q13: If you performed a Posterior L2 corpectomy and expandable cage, would you be willing to sacrifice the L2 nerve root to get your cage in?

- I would not perform a Posterior corpectomy and cage
- No - I would absolutely not sacrifice the L2 nerve root
- Yes - if I had to (try to place cage without taking it first)
- Yes - I would plan on sacrificing to place cage

Title: Femoral Shaft Fracture in 27M

Category: Trauma

Popularity: 1

Date: 20230723

Link: <https://www.orthobullets.com/Site/Cases/View/96c03428-4554-497d-b45e-f9d79c4e45a4>

Images:

ChatGPT-3.5 dialogue:

I am going to provide you a clinical vignette. There will be a series of 14 questions to follow. Please choose the single best response for each question.

History of presenting incident:

A 27-year-old male is involved in MVC at 50 mph.

Past medical history:

N/A PMH

Social ETOH

Physical exam:

RLE

- inspection shows thigh swelling and deformity with ecchymosis

- 2+ DP and PT pulse

- 5/5 ankle DF/PF and EHL

Imaging findings:

- Plain AP and lateral x-ray radiographs show a left comminuted femoral shaft fracture. Plain AP pelvis and AP left knee do not reveal additional injuries.

Q1: Would you get additional imaging of the Femoral FX to determine treatment?

- No – current x-rays are sufficient.
- Yes – additional x-ray views.
- Yes – CT scan.
- Yes – additional x-ray views + CT scan.

Q2: Would you get additional imaging to rule out an Ipsilateral Femoral Neck FX?

- No - Current Xrays are sufficient
- Yes - preop Additional Xray views ONLY
- Yes - preop Fine-cut CT ONLY
- Yes - preop Additional Xray views + Fine-cut CT
- Yes - intraop Fluoroscopic views ONLY
- Yes - preop Additional Xray views + intraop Fluoroscopic views
- Yes - preop Additional Xray views + Fine-cut CT + intraop Fluoroscopic views

Q3: Would you use a classification system to guide your treatment of the femur fx?

- No - a classification system does not help me determine treatment
- Yes - Winquist classification system
- Yes - OTA classification system

Q4: How would you manage this patient?

- Nonoperative - Skeletal traction until union
- Operative

Q5: If you chose Operative management, would you temporize this patient prior to definitive fixation?

- I would not choose operative management
- No - I would operate acutely (within 12 hours)
- Yes - temporize short-term with Buck's external traction
- Yes - temporize short-term with Skeletal traction
- Yes - temporize short/long term with External Fixation

Q6: If you choose to temporize with Skeletal Traction, what type would you use?

- I would not temporize with Skeletal Traction
- Distal Femoral Traction Pin
- Proximal Tibial Traction Pin
- Calcaneal Traction Pin

Q7: If you choose Operative management, what Definitive Fixation technique would you use?

- I would not choose operative management
- External fixation
- Intramedullary nail fixation (IM Nail fixation)
- ORIF (plate, screws, wires)
- IM Nail fixation + ORIF (plate, screws, wires)

Q8: If you choose IM Nail fixation or External fixation, would you perform Open Reduction of the fracture site?

- I would not choose IM Nail fixation or External Fixation
- No
- Yes

Q9: If you choose IM Nail fixation, what approach would you use?

- I would not choose IM Nail fixation
- Antegrade - Trochanteric entry
- Antegrade - Piriformis entry
- Antegrade - Lateral entry
- Retrograde

Q10: If you choose IM Nail fixation, how would you position the patient and what table would you use?

- I would not choose IM Nail fixation
- Lateral patient position - Fracture table
- Lateral patient position - Radiolucent flattop table
- Supine patient position - Fracture table
- Supine patient position - Radiolucent flattop table

Q11: If you choose IM Nail fixation, what would be your PRIMARY method to get your femoral LENGTH correct?

- I would not choose IM Nail fixation
- Preop Templating - Nail length using contralateral femur
- Intraop Open - Direct visualization of Fx reduction
- Intraop Fluoro - Imaging of Fx reduction
- Intraop Flouro - Radiopaque ruler measurement vs. contralateral femur
- Clinical inspection - Palpate plantar surface of foot/ankle vs. contralateral side
- Clinical inspection - Measure length of leg vs contralateral side with string or ruler (Greater troch. to Lateral mal.)

Q12: If you choose IM Nail fixation, what would be your PRIMARY (most valuable) method to get your femoral ROTATION correct?

- I would not choose IM Nail fixation
- Preop Templating - Nail-screw angle vs. Contralateral
- Intraop Fluoro - Cortical Thickness
- Intraop Fluoro - Lesser trochanter profile (LTP)
- Intraop Fluoro - Femoral neck horizontal angle (NHA)
- Intraop Fluoro - True lateral technique (TLT)
- Clinical Inspection - Look at direction foot is pointing vs. contralateral side
- Clinical Inspection - Look at skin wrinkling

Q13: In this patient, if you choose IM Nail fixation, when would you begin FULL Weight Bearing as Tolerated?

- I would not choose IM Nail fixation
- Immediately
- Wait 3 Days
- Wait 1 Week
- Wait 2 Weeks
- Wait 3 Weeks
- Wait 4 Weeks
- Wait 6 Weeks
- Wait 8 Weeks
- Wait until Fx healing seen on xrays - then begin

Q14: In this patient, if you choose IM Nail fixation, how long would you prescribe postoperative DVT prophylaxis?

- I would not choose IM Nail fixation
- I would NOT prescribe DVT prophylaxis
- Until the patient is ambulatory
- 1 week postop
- 2 - 4 weeks postop
- 5 - 7 weeks postop
- 8 - 10 weeks postop
- 11 - 12 weeks postop
